# Rapid and Energetic Solid-State Metathesis Reactions for Iron, Cobalt, and Nickel Boride Formation and Their Investigation as Bifunctional Water Splitting Electrocatalysts

**DOI:** 10.1021/acsmaterialsau.1c00079

**Published:** 2022-04-21

**Authors:** Janaka P. Abeysinghe, Anna F. Kölln, Edward G. Gillan

**Affiliations:** Department of Chemistry, University of Iowa, Iowa City, Iowa 52242, United States

**Keywords:** crystalline metal borides, solid-state metathesis, exothermic, thermochemistry, cobalt boride, nickel boride, hydrogen evolution electrocatalysis, oxygen evolution electrocatalysis

## Abstract

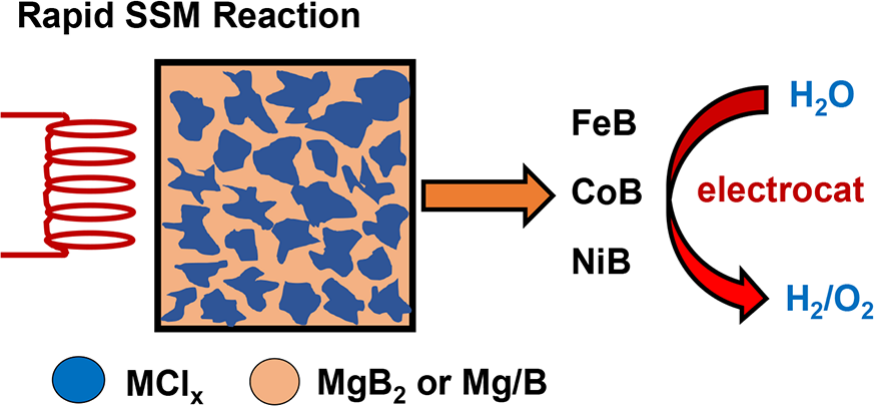

Metal borides have long-standing uses due to their desirable chemical and physical properties such as high melting points, hardness, electrical conductivity, and chemical stability. Typical metal boride preparations utilize high-energy and/or slow thermal heating processes. This report details a facile, solvent-free single-step synthesis of several crystalline metal monoborides containing earth-abundant transition metals. Rapid and exothermic self-propagating solid-state metathesis (SSM) reactions between metal halides and MgB_2_ form crystalline FeB, CoB, and NiB in seconds without sustained external heating and with high isolated product yields (∼80%). The metal borides are formed using a well-studied MgB_2_ precursor and compared to reactions using separate Mg and B reactants, which also produce self-propagating reactions and form crystalline metal borides. These SSM reactions are sufficiently exothermic to theoretically raise reaction temperatures to the boiling point of the MgCl_2_ byproduct (1412 °C). The chemically robust monoborides were examined for their ability to perform electrocatalytic water oxidation and reduction. Crystalline CoB and NiB embedded on carbon wax electrodes exhibit moderate and stable bifunctional electrocatalytic water splitting activity, while FeB only shows appreciable hydrogen evolution activity. Analysis of catalyst particles after extended electrocatalytic experiments shows that the bulk crystalline metal borides remain intact during electrochemical water-splitting reactions though surface oxygen species may impact electrocatalytic activity.

## Introduction

Hydrogen is an important fuel source alternative to fossil fuels due to its high gravimetric energy density (∼120 kJ/g) and low environmental footprint.^1,2^ Typical hydrogen production uses fossil fuels as starting materials, so there is strong interest in electrochemically splitting water into hydrogen and oxygen for sustainable energy uses. Sluggish kinetics for electrochemical water splitting, particularly the oxygen evolution reaction (OER), remains a challenging issue that limits large scale hydrogen production.^3^ Expensive and precious-metal catalysts, such as Pt for the hydrogen evolution reaction (HER)^4,5^ and RuO_2_ and IrO_2_ for OER,^6,7^ show high electrocatalytic water splitting activity, though in some cases limited long-term stability. Over the last several decades, emphasis has been placed on the synthesis of chemically stable electrocatalysts formed using relatively abundant transition-metals. A wide range of transition-metal phosphides, nitrides, sulfides, oxides, carbides, and borides have been reported as promising electrocatalysts for different aspects of the electrochemical water splitting reaction.^8−14^ Recent reports on P-rich metal phosphides (e.g., CoP_3_, NiP_2_)^15−17^ and B-rich metal borides (e.g., VB_2_, MoB_2_)^18−21^ show appreciable activity in HER electrocatalysis, indicating that the nonmetal components of HER electrocatalysts may play an important role in solution-surface bonding interactions and oxidation–reduction processes that take place during electrocatalysis.

Metal borides (MBs) are an intriguing class of materials that are less studied for water splitting electrocatalysis. Some borides have refractory properties such as high melting points, hardness, thermal stability, wear resistance, corrosion resistance, and chemical stability that may be useful in harsh electrochemical environments.^22−30^ There are fewer synthetic routes to crystalline metal borides than related oxides, sulfides, or phosphides, possibly due to the relative stability of elemental boron. Boron can function as an electron-poor Lewis acid in molecular structures, but in borides it may have electron-rich anionic character in extended boron–boron bonding environments. Boron-rich metal borides show good chemical resistance due to the presence of strong covalent boron–boron networks.^31^ Recent studies indicate that the electrocatalytic water splitting activity of boron-rich metal borides may be higher than that of metal-rich borides.^18,19^ In order to better assess electrocatalytic activities of metal borides, a facile and flexible approach to crystalline well-structured metal boride synthesis is desirable.

Metal boride syntheses often rely on mechanochemical methods^32−35^ and high-temperature or high-energy syntheses.^36−38^ Solution-phase reduction in aqueous^39,40^ or organic^41,42^ solvents generally yields amorphous products that require high-temperature annealing to form crystalline metal borides. Boron-rich metal borides containing earth-abundant iron, cobalt, and nickel have been synthesized by solution chemical reduction^40,41^ and high temperature heating.^43−46^ There are several examples of combustion-like reactions between elemental magnesium and boron with metal oxides that form crystalline metal borides.^47^ Metal borides have been examined as HER or OER electrocatalysts, though they are often studied as amorphous or disordered metal boride nanostructures.^48−53^ There are a few examples of crystalline metal-rich 3d metal borides with activity as electrocatalysts (e.g., Fe_2_B,^18^ Co_2_B,^35,54^ Co_3_B,^55^ Ni_3_B.,^56^ and AlFe_2_B_2_^57^).

A rapid and solvent-free synthetic alternative to conventional solution reduction or direct elemental reactions is the solid-state metathesis (SSM) reaction.^58,59^ SSM reactions take advantage of highly exothermic and thermochemically driven exchange reactions that provide a rapid high yield route to *crystalline*, nanometer or micrometer-sized products, with little external energy. SSM reactions between precursors can rapidly (∼3 s) achieve high temperatures (>1000 °C) followed by fast cooling (∼30 s), which is often sufficient to facilitate product nucleation and particle growth and crystallization in a molten byproduct salt flux.^59−62^ While high-temperature syntheses often produce thermodynamically stable phases, the rapid cooling of SSM reactions can produce metastable phases.^63,64^ Self-sustaining SSM reactions may be initiated by local heating (e.g., hot filament or flame) or by full volume reactant heating using an external furnace. In either mode, a rapidly formed molten alkali or alkaline-earth halide byproduct salt can transiently reach temperatures at or above ∼1200 °C for a brief period and aid in reactant diffusion, redox processes, and product crystallization. The SSM reaction strategy has been successful for the synthesis of a wide range of crystalline metal/nonmetal compounds including crystalline metal nitrides,^65−67^ metal phosphides,^68^ metal oxides,^61^ metal sulfides,^69^ and several transition-metal and rare-earth borides.^20,27,64,70^

Much of the developmental survey work performed in the mid 1990s on *rapid* SSM reactions focused primarily on early and midtransition metals and surprisingly do not report the synthesis and characterization of earth-abundant borides of Fe, Co, or Ni.^58,59,64^ SSM synthesis of early transition-metal diborides (*e.g.*, TiB_2_, ZrB_2_, HfB_2_, VB_2_, CrB_2_, NbB_2_) and recent MoB_2_ synthesis demonstrate that MgB_2_ functions as a boron source in SSM reactions and provide guidance for the syntheses described here.^20,64,70^ One study briefly mentioned FeB formation by an ignition SSM reaction, but no characterization is provided.^64^ Our work here demonstrates that solvent-free SSM reactions can be utilized for the rapid synthesis of crystalline metal (Fe, Co, and Ni) monoboride microparticles in seconds. The successful use of an existing MgB_2_ SSM reactant for these metal boride reactions is compared to a more tunable SSM reaction strategy using mixed Mg/B powder reactants. These SSM reaction methodologies access late-transition metal monoborides using reactants that do not contain either hydrogen or oxygen and may open doors to other rapid thermochemically driven metal boride synthesis and related reactions with other nonvolatile elemental reactants. This work also examines the water splitting activity of these crystalline metal boride particles on conducting carbon wax electrodes used in our recent metal phosphide studies.^16^ These earth-abundant borides may find use as chemically robust electrocatalyst alternatives to precious metals. Several of these metal monoborides show moderate activity in HER and OER electrocatalysts. The carbon wax electrodes allow post reaction direct examination of surface and bulk catalyst structures by microscopy and X-ray diffraction.

## Experimental Procedures

### Starting Materials and Reagents

Anhydrous commercial reagents were stored in an inert atmosphere glovebox and utilized as purchased: NiCl_2_ (Alfa-Aesar, 99%), CoCl_2_ (Alfa-Aesar, 99.7%), FeCl_3_ (Alfa-Aesar, 98%), FeCl_2_ (Aldrich Chemicals, 98%), MgB_2_ (Alfa-Aesar, 99%), Mg (Sigma-Aldrich, 99.5%, powder, −325 mesh), amorphous B (Alfa-Aesar, 95–97%, powder (APS < 1 μm)), and MgCl_2_ (Cerac, 99.9%, powder, 100 mesh). HCl at 0.1 M (Fisher Scientific, 12.4 M diluted with DI H_2_O) was used for the product washing. ICP calibration standards were prepared by diluting Co (Alfa-Aesar, 999 ± 5 μg/mL), Ni (Alfa-Aesar, 1003 ± 6 μg/mL), Fe (Alfa-Aesar, 1003 ± 6 μg/mL), and B (Inorganic Ventures, 9968 ± 52 μg/mL) in 5 vol % HNO_3_ (from Sigma-Aldrich, 14 M diluted in 18 MΩ ultrapure water) and by dissolving Mg powder (Sigma-Aldrich, 99.5%, −325 mesh powder) in 5 mL of conc. HNO_3_ and then diluting to 100 mL with water. Materials used for electrochemical studies: synthetic graphite powder (Sigma-Aldrich, <20 μm), paraffin wax (Sigma-Aldrich, mp ≥ 65 °C), 0.1 and 1.0 M KOH (Sigma-Aldrich, KOH pellets dissolved with 18 MΩ ultrapure water), 0.5 M H_2_SO_4_ (Fisher Scientific, 95–98%, 18 M diluted with 18 MΩ ultrapure water), 10% Pt on Vulcan XC-72 carbon (C1- 10 fuel cell grade, E-Tek), and RuO_2_ (Alfa-Aesar, 99.9%).

### Reaction Safety Considerations

The reactants for highly exothermic SSM reactions may initiate with explosive action and a rapid evolution of the gas or volatilization of reactants. Grinding-induced reaction initiation can occur with some SSM reactions, but it was not observed with the late transition MB synthesis reactions performed here. It is strongly advised to evaluate proposed SSM reactions for exothermicity and reactant phase change behavior before performing a reaction. Several milligram amounts should be carefully ground together with a mortar/pestle to examine friction-induced reaction initiation. It is best practice to perform rapid and exothermic SSM reactions in a contained environment (steel reactor or thick glass ampules or vented containment systems) and examine new reactions on a scale of less than 1 g of reactants. Postsynthesis product manipulations (e.g., opening of the reactor, grinding, and washing the product) should be carried out inside the fume hood to prevent inhalation of hazardous volatiles or nanosized particles.

### SSM Synthesis of Metal Borides: MB (M = Fe, Co, Ni)

**Figure 1:**
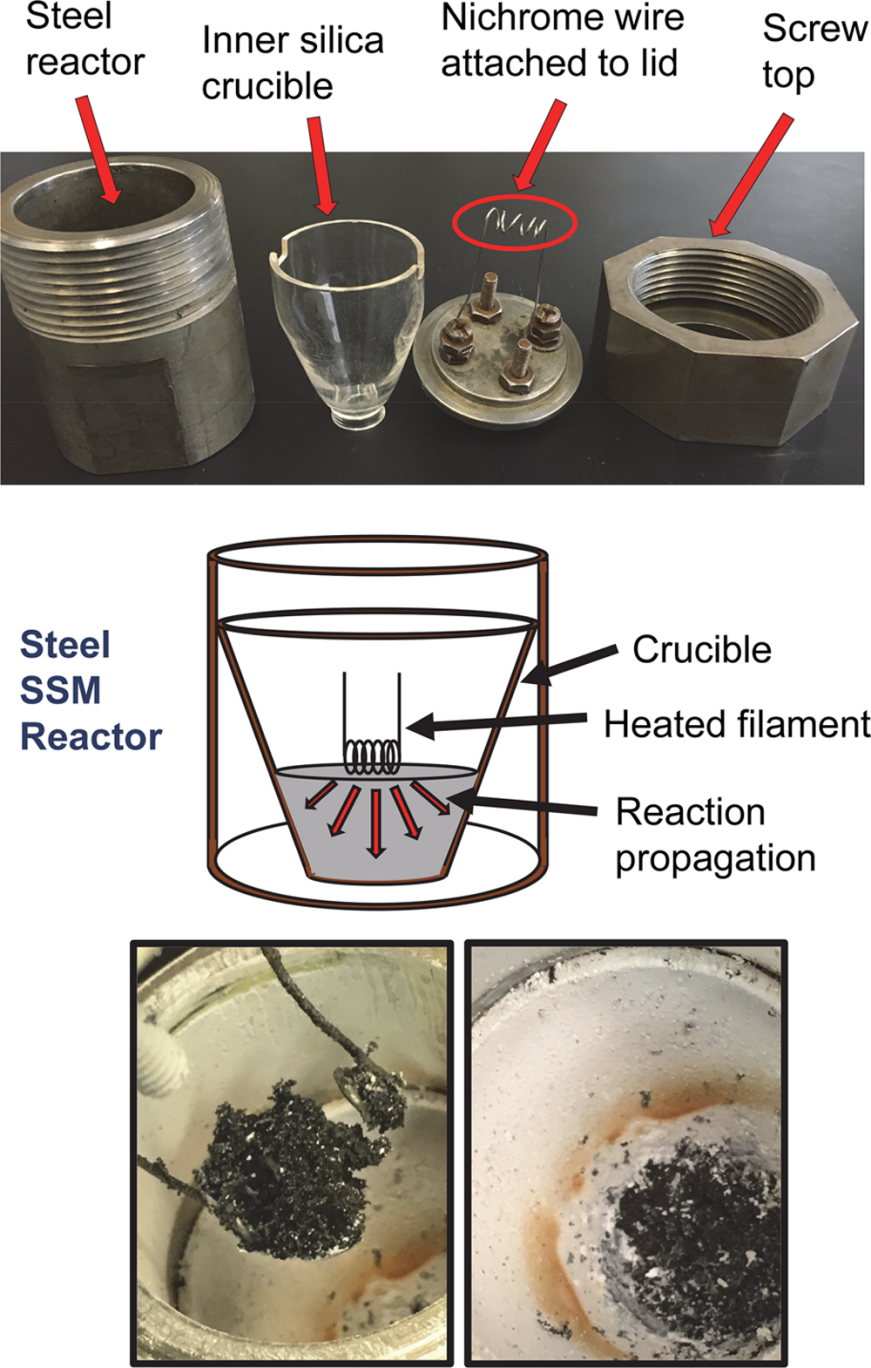
Components of the ignition-SSM reactor (top). Schematic diagram of SSM reactor and reaction propagation (middle). Images of black metal borides formed around the ignition wire at bottom of the cup with white MgCl_2_ deposits on crucible walls and combined with the metal boride (bottom).

The formation of three metal borides was investigated using two different SSM reaction routes, a double displacement metathesis reaction between anhydrous metal halides (MCl_*x*_) and MgB_2_ or a three-component displacement and redox reaction between MCl_*x*_ and Mg/B mixtures. Reaction stoichiometries were chosen to produce a balanced amount of MgCl_2_ salt byproduct based on the amount of metal halide used. Typical reactant amounts used in these SSM reactions were 6 mmol MCl_*x*_ (FeCl_2_, CoCl_2_, or NiCl_2_) with 6 mmol MgB_2_ (or 6 mmol Mg and 12 mmol B) and for the FeCl_3_ reactions, 5 mmol FeCl_3_ were reacted with 7.5 mmol MgB_2_ (or 7.5 mmol Mg and 5 or 10 mmol B). The combined mass of reactants used was approximately one gram. Several reactant modifications were examined, specifically FeCl_3_ versus FeCl_2_ and variations in boron amounts for Mg/B reactions. Since rapid SSM reactions are complete in seconds, intimate mixing of reactants is important to promote complete and homogeneous reactions. In an argon-filled glovebox, the MCl_*x*_ powders were ground separately (∼30 s) in an agate mortar and then combined with either MgB_2_ or Mg/B mixtures (Mg and B added sequentially to MCl_*x*_ and ground together) and finally all reactants were ground together (∼1 min) to obtain a homogeneous powder mixture. The reactant mixture was transferred to a cone-shaped quartz crucible (top OD = 3.6 cm, bottom OD = 1.3 cm, height = 4.9 cm), which was placed in a cylindrical shaped stainless-steel reactor (ID = 3.8 cm, OD = 5.0 cm, height = 6.2 cm) with a loose screwcap lid (Figure 1).^59,61,64^ A coiled nichrome wire with five loops to increase the contact with the reactant powder mixture was attached to two electrical feedthroughs on the lid. The closed reactor was transferred from the glovebox to a fume hood.

A Variac transformer set to 15.5 V was connected to the nichrome filament and turned on for about 6 s to resistively heat the filament. Within ∼3 s, resistive heating causes the temperature of the nichrome wire to rise to ∼750–800 °C. Reaction initiation is typically detected by wisps of white smoke exiting from the edges of the reactor lid. After the SSM reactions were cooled to near room temperature, the reactor was opened and its contents, a dark product mass and white salt byproduct (*cf.*, Figure 1), were ground to a fine powder. Unreacted starting materials and byproduct MgCl_2_ were removed by washing with 50 mL of stirred 0.1 M HCl for 30 min, followed by washing with 75–100 mL of distilled water for 30 min. After each washing step, the product powders were isolated by centrifugation. The products were oven-dried for 20 min at 130 °C, and mass yields are calculated using the recovered product weight and theoretical masses of metal boride and boron products.

### Examination of SSM Intermediates in Metal Boride Synthesis

Filament initiated SSM reactions between MCl_2_ and magnesium powder were investigated in the stainless-steel reactor. In a glovebox, anhydrous MCl_2_ was ground to a fine powder in an agate mortar for 1 min and then Mg powder was added, and the two reactants were ground for ∼1 min to form a homogeneous powder mixture. This mixture was initiated and worked up in a similar manner used for metal boride SSM reactions described above. The product was oven-dried for 20 min at 130 °C. The isolated metal powders from these reactions were ground with amorphous boron in air, vacuum-dried for ∼5 min with a heat gun, and then flame and sealed in an evacuated Pyrex ampule. The ampule was placed in the horizontal clamshell tube furnace and heated at 100 °C/h and held at 500 °C for 3–4 days. The solid samples were washed with 0.1 M HCl and then DI water, centrifuged and oven-dried for 20 min at 130 °C.

### Sample Characterization

Powder X-ray diffraction (XRD) was performed to characterize the structure and crystallinity of boride products using a Bruker D8 DaVinci diffractometer with nickel-filtered Cu Kα X-ray irradiation (40 kV, 40 mA) from 5 to 80° 2θ (0.05° step size). Ground metal boride powders were placed on a vacuum greased glass slide. Reference XRD patterns and crystal structure representations were generated using the Crystal Maker software (http://www.crystalmaker.com/index.html) and literature data for orthorhombic FeB, CoB, and NiB.^71−73^ The morphologies and particle sizes of the products were identified via scanning electron microscopy (SEM) using a Hitachi S-4800 field emission scanning electron microscope at 5 kV and transmission electron microscopy (TEM) using a Hitachi S-7800 transmission electron microscope with an accelerating voltage of 80 kV. Ground samples were adhered to carbon tape on aluminum stubs for SEM analysis and were sonicated in methanol and drop cast on carbon coated Ni mesh grids for TEM analysis. Qualitative element specific mapping was performed by energy dispersive spectroscopy (EDS) with SEM on bulk powders and metal borides affixed to carbon wax electrode tips. Surface areas were obtained from Brunauer–Emmett–Teller (BET) measurements on a Quantachrome Nova 1200 nitrogen surface area analyzer using vacuum-dried samples (120 °C for ≥2 h). Inductively coupled plasma-optical emission spectroscopy (ICP-OES) measurements for bulk elemental analysis were made using a PerkinElmer Optima 7000 DV ICP-OES spectrometer. The metal boride samples were dissolved in heated 5 mL of concentrated HNO_3_ and diluted to 100 mL with 18 MΩ ultrapure water. Linear elemental calibration curves were produced from commercial ICP standards (Fe, Co, Ni, and B) diluted in 5% HNO_3_ or dried Mg powder dissolved in 5% HNO_3_. X-ray photoelectron spectroscopy (XPS) measurements were performed on powders pressed on indium foil with a Kratos AXIS Ultra DLD X-ray photoelectron spectrometer using monochromated Al Kα X-rays and charge neutralization. Peak positions were referenced to adventitious carbon at 285 eV. XPS peak analysis was performed using CasaXPS software (www.casaxps.com).

### Working Electrode Preparation

Working electrodes for electrocatalytic measurements were prepared using graphite/paraffin wax mixture (45% graphite: 55% wax) inside a PTFE tube (C_wax_ electrode) as previously reported by our group.^16^ This type of conducting carbon with an adherent (sticky) surface has shown utility in several prior electrochemical studies.^74,75^ Working electrode tips were 1.4 cm long, 3.2 mm ID, and 6.4 mm OD, with an 0.080 cm^2^ geometrical surface area (Figure S1). The wax was softened in a 55 °C water bath for 30 min, and then ground metal boride powders were gently pressed onto the softened C_wax_ electrode surface. Sample mass loadings on the electrode typically ranged from ∼0.5–1.5 mg. An Al connecting rod in a Teflon tube was embedded into the back of the wax for connection to the potentiostat.

### Electrochemical Measurements

Electrochemical measurements were performed using a three-electrode cell with a C_wax_ working electrode, Hg/HgO reference electrode (20% KOH) for 0.1 and 1.0 M KOH electrolytes or Hg/Hg_2_Cl_2_ (SCE) reference electrode for 0.5 M H_2_SO_4_ electrolyte, and a platinum mesh or Pt wire counter electrode (Figure S1). Comparison studies were made using a graphite rod counter electrode (Alfa Aesar, 6.2 mm diam., SPK grade, 99.9995%). Electrochemical measurements were carried out primarily in 0.1 M KOH electrolyte solutions, but comparison measurements were made in 0.5 M H_2_SO_4_ and 1.0 M KOH. The Hg/HgO electrode potential values were converted to standard hydrogen electrode potentials using the equation *E*_RHE_ = *E*_Hg/HgO_ + 0.059pH + *E*_0,Hg/HgO_, with pH = 13 (0.1 M KOH) or 14 (1.0 M KOH) and *E*_0,Hg/HgO_ = 0.098 V. The SCE electrode potential values were converted to standard hydrogen electrode potentials using the equation *E*_RHE_ = *E*_SCE_ + 0.059pH + *E*_0,SCE_, with pH = 0.3 (0.5 M H_2_SO_4_) and *E*_0,SCE_ = 0.241 V. Reference electrodes were verified as working correctly using hydrogen calibration methods with a platinum wire,^76^ and electrolyte pH values were measured with a pH meter. These measurements confirmed the standard RHE conversions, and pH values are reliable to within ∼10 mV (summary data in Table S1). The electrodes were attached to a Bioanalytical Systems BASi 100b potentiostat and placed in a single compartment cell, which consists of a Pyrex beaker and a PTFE lid, similar to the setup used in our prior work.^16^ A magnetic cross stir bar was placed under the working electrode (∼6 mm away) at ∼400 rpm during the electrochemical measurements to remove gas bubbles (H_2_ or O_2_) that form on the electrode surface. The electrolyte solutions were purged with O_2_ or H_2_ gases that were prehumidified by passing them through a water bubbler. The gas purging began 30 min before electrochemical measurements and continued throughout the experiment. All potentials are referenced to RHE values unless indicated. Metal boride OER and HER activities and stability were evaluated using 50 linear sweep voltammograms (LSVs) in O_2_ (99.5% purity, Praxair) purged electrolyte for OER or in H_2_ (ultrahigh purity 99.999%, Praxair) purged electrolyte for HER. Reported current densities are scaled relative to the geometric area of the C_wax_ electrode (0.080 cm^2^). The LSV data were obtained without *iR* compensation, as recent studies caution that *iR* compensation in electrocatalysis may obscure differences in catalyst activity, such as undesirably compensating for differences in catalyst charge transfer interactions.^77,78^ Data using the potentiostat’s 85% *iR* compensation was obtained on Mg/B reaction products in different electrolytes for comparison. The measured cell resistances in different electrolytes for catalysts on carbon wax electrodes show that 0.1 M KOH (∼120–150 Ω) is larger than 1.0 M KOH (∼40–60 Ω) and 0.5 M H_2_SO_4_ (35–55 Ω). Standard data for RuO_2_ OER and 10%Pt/C HER are provided for comparison purposes and to show their performance when affixed to the C_wax_ electrode surface.

The electrochemical surface areas (ECSAs) of each metal boride were determined by measuring the double-layer capacitance (*C*_dl_) in the non-Faradaic region with N_2_ gas purging.^16,78^ ECSA analysis was performed using cyclic voltammetry (CV) data at scan rates of 5, 10, 25, 50, and 75 mV/s after five conditioning CV runs at 5 mV/s in the potential range of 175–475 mV before 18 h chronoamperometry runs. Calculated capacitance values were converted to approximate areas using a 35 μF/cm^2^ relationship.^79^ The long-term activity of metal borides in both HER and OER was investigated using 18 h time base chronoamperometry studies (CA) at constant potentials in 0.1 M KOH with chosen applied potentials targeting approximately a current density of ∼10 mA/cm^2^.

### Postelectrochemistry Metal Boride Analysis

After 18 h chronoamperometry experiments, the crystallinity of FeB, CoB, and NiB catalysts was investigated using powder XRD (27–60° 2θ, 0.02° step size). XRD samples were made from postelectrochemical electrodes by cutting ∼1–2 mm slices from the end of the C_wax_ electrode containing a thin surface coating of embedded metal boride powders and placing them in the well of an XRD sample holder for XRD analysis (Figure S1). The height of the C_wax_ electrode surface was adjusted to ensure that the particles on the wax electrode surface were in the correct diffraction position for analysis. EDS mapping was also obtained for the catalysts after chronoamperometry. For comparison purposes, XRD and EDS mapping data were also obtained for metal boride powders embedded on C_wax_ electrode tips to approximate a pre-electrochemistry electrode structure.

## Results and Discussion

### Metal Boride Synthesis via SSM Reactions

Rapid SSM reactions rely on thermochemical instability between reactants that favors self-sustaining exchange reactions to produce crystalline products. New SSM reaction methodologies may provide easy access to crystalline structures with earth-abundant elements. Solvent-free exothermic SSM reactions are developed here for the rapid synthesis of *crystalline* metal monoborides of Fe, Co, and Ni. Prior SSM synthesis demonstrated that MgB_2_ can serve as a boron source and provide guidance for the current work.^20,64,70^ One important decision for SSM reactants is choosing those that lead to a significant release of energy (∼200 kJ/mol) during the reaction. Since these reactions initiate via diffusion-limited solid–solid particle surface reactions, it is important to use intimately mixed powder reactants to promote SSM reaction propagation and completion. Once initiated, SSM reactions can include melting and vaporization of reactants and products. The exothermic metal boride SSM reactions shown in eqs 1 and 2 are salt-balanced and react MgB_2_ with metal chlorides (MCl_2_ or MCl_3_) to produce MgCl_2_ and a monoboride product with excess boron. They rapidly ignite to a self-propagating state, yielding black products and a white MgCl_2_ transport (Figure 1).
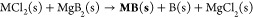
1
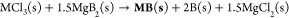
2

**Figure 2:**
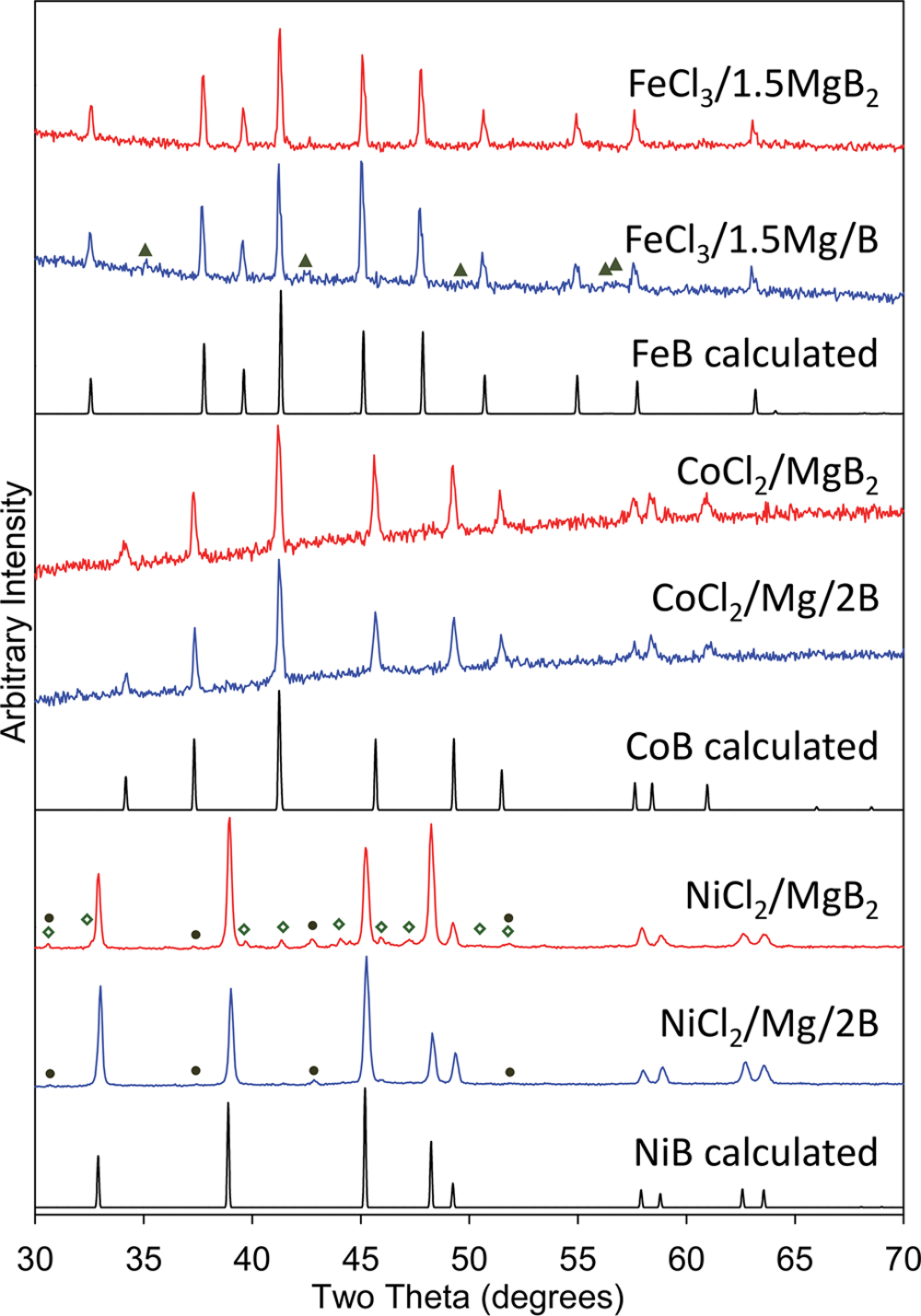
Powder XRD results for orthorhombic metal monoborides. The stack plot is ordered as the product from the MgB_2_ reaction (red) and Mg/B reaction (blue) and the calculated MB pattern (black). Small impurity peaks were identified as *m*-Ni_4_B_3_ (green open tilted square), MgNi_3_B_2_ (black solid circle), Fe_2_B (dark green solid up triangle).

Crystalline single-phase powders of FeB, CoB, and NiB were synthesized with high yields (∼80%) from the MCl_*x*_/MgB_2_ reactions using NiCl_2_, CoCl_2_, or FeCl_3_ as indicated by powder XRD (Figure 2). The NiB pattern shows small peaks consistent with *m*-Ni_4_B_3_ and/or MgNi_3_B_2_ side products. The atomic ratios of NiB and Ni_4_B_3_ are similar and the formation of one phase in the absence of the other is difficult.^43^ Reactions with FeCl_2_ produce FeB but with lower yields (∼65%). An analogous SSM reaction of CuCl_2_/MgB_2_ produced Cu instead of CuB_*x*_, consistent with the Cu–B phase diagram shows that no stable CuB_*x*_ phases exist.^80^ The reaction stoichiometries in eqs 1 and 2 provide sufficient boron for MB_2_ compositions. In the thermodynamic phase diagrams of Fe, Co, or Ni with boron, the monoborides are the highest boron content stable phases.^80^ In other synthetic routes, the formation of metal-rich borides readily occurs, and excess boron is required to form boron-rich target phases instead of metal-rich borides.^45,81,82^

Given the success with MCl_*x*_/MgB_2_ reactions, analogous SSM reactions were developed using powdered Mg and amorphous boron (Mg/B) reactants instead of MgB_2_. In theory, these Mg/B reactions are more exothermic than MgB_2_ reactions due to the stable heat of formation of MgB_2_ (Δ*H*_f_ = −60.7 kJ/mol). One potential disadvantage is that three reactants must interact effectively in the very short time frame of a rapid exothermic SSM reaction. SSM reactions involving three reactants can be salt-balanced similar to the MgB_2_ reactions shown above. This modified SSM approach shown in eqs 3 and 4 will theoretically obtain similar products MB and MgCl_2_ to the MgB_2_ reactions with a potential advantage of improved reactant compositional tuning through Mg/B variations that are not available using the MgB_2_ reactant.
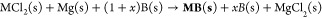
3
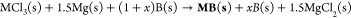
4

Reactions with stoichiometric boron (*x* = 0) were unsuccessful for CoCl_2_ and NiCl_2_ as the cobalt reaction produced CoB with elemental Co and the Ni reaction produced mainly MgNi_3_B_2_ with Ni_2_B, NiB, and *m*-Ni_4_B_3_ as minor products (Table S2). High temperature/pressure reactions are known to form various phases of Mg–Ni–B and Ni–B from Mg/Ni/NaBH_4_ reactions.^83^ The stoichiometric FeCl_3_ reaction (eq 4, *x* = 0) was successful in producing FeB, and CoB and NiB were successfully produced from CoCl_2_ and NiCl_2_ reactions with Mg/2B (*x* = 1) reactant amounts (Figure 2). The MCl_*x*_ reactions with Mg/B reactants are rapid and self-sustaining after hot wire initiation and produce crystalline FeB, CoB, and NiB with good yields (>70%). The MCl_2_/Mg/2B stoichiometry forms MB+B, similar to the MCl_2_/MgB_2_ reactions (eq 1 vs 3 for *x* = 1). The XRD results for crystalline monoborides from MgB_2_ and Mg/B SSM reactions are similar ,and their average XRD crystallite sizes are about 40–50 nm (Table S3).

The FeB XRD data in Figure 2 from the stoichiometric reaction (FeCl_3_/1.5Mg/B) contains possible trace peaks for Fe_2_B. Single-phase crystalline FeB was obtained using the stoichiometry in eq 4 with excess boron (*x* = 1). In contrast, the FeCl_2_/Mg/B reaction resulted in a mixture of FeB and Fe_2_B, and excess boron (FeCl_2_/Mg/2B) was required to obtain single-phase FeB (Table S4 and Figure S2). These results indicate that MCl_2_ reactions with Mg need excess boron to form monoboride products. There are various factors influencing these reactions such as reaction thermochemistry differences, reactant volatility/stability, and M/B diffusion limitations in the MgCl_2_ molten salt. Thermochemical analyses and mechanistic studies are described later to help understand reaction progression in these Mg/B systems.

The three monoborides synthesized from layered MgB_2_ or amorphous boron have similar orthorhombic structures that consist of boron zigzag chains interspersed with metals. The shortest bonds are in the boron chains (B–B of ∼1.73–1.92 Å) and metal–boron bonds (M–B of ∼2.05–2.18 Å), with longer M–M distances near 2.6 Å. Several structural representations of FeB, CoB, and NiB are shown in Figure S3.^71−73^ In these structures, M–B and B–B bonding interactions would present themselves on different low index crystal planes of solid particles and influence surface bonding that occurs during catalytic solution reactions.

### Chemical and Physical Properties of SSM Synthesized Metal Borides

Bulk chemical analysis by ICP-OES shows that the metal boride products generally have compositions expected based on their SSM reaction stoichiometries and observed crystalline XRD phases (Table 1). These compositional results are consistent with excess boron based on the balanced SSM reactions. The low residual magnesium content indicates that the wash process is effective at removing the MgCl_2_ byproduct or remaining Mg reactants. For the FeB reaction with MgB_2_, higher Mg and B levels indicate the presence of some MgB_2_, a noncrystalline Mg–Fe–B phase, or MgCl_2_. The M:B molar ratio for the NiCl_2_/MgB_2_ reaction product is lower than its ideal 1:2 ratio, consistent with the observed trace formation of metal-rich Ni and Ni/Mg phases by XRD. The stoichiometric FeB reaction from Mg/B produces primarily FeB with an Fe:B ratio near 1:1.

**Table 1:** Analytical Results for FeB, CoB, and NiB from SSM Reactions Using MgB_2_ or Mg/B

SSM reaction molar ratios	target phase(s)	% yield (target)	XRD (major phase bolded)	ICP-OES M/B/Mg molar ratio	BET (m^2^/g)
FeCl_3_/1.5MgB_2_	FeB+2B	82	**FeB**	1.00/3.26/0.146	8
FeCl_3_/1.5Mg/B	FeB	73	**FeB**, trace Fe_2_B	1.00/1.14/0.012	18
CoCl_2_/MgB_2_	CoB+B	80	**CoB**	1.00/1.88/0.035	3
CoCl_2_/Mg/2B	CoB+B	85	**CoB**	1.00/1.72/0.020	2
NiCl_2_/MgB_2_	NiB+B	81	**NiB**, Ni_4_B_3_, MgNi_3_B_2_	1.00/1.57/0.070	2
NiCl_2_/Mg/2B	NiB+B	86	**NiB**, MgNi_3_B_2_	1.00/2.06/0.034	3

One negative aspect of the three-component Mg/B reactions is that, for incomplete reactions, the amorphous boron reactant will remain with the product after washing. This contrasts with MgB_2_ reactions where the unused reactant is removed by washing (but balanced reactions may produce excess boron). We attempted postreaction removal of excess boron from several reaction products, but the acidic forcing conditions required for boron dissolution (heated nitric acid) also dissolve the monoborides. All characterization and catalysis reported here are for products shown in Table 1, containing additional amorphous boron except for FeB from the Mg/B reaction.

The metal boride powders from these rapid SSM reactions have relatively low to moderate external surface areas (Table 1). These surface areas indicate that the boride products are nonporous solids and the short nucleation/crystal growth times produce aggregates of large nanoparticles or submicrometer particles in molten MgCl_2_. The highest surface areas are observed for FeB formed via both MgB_2_ and Mg/B elemental reactions.

**Figure 3:**
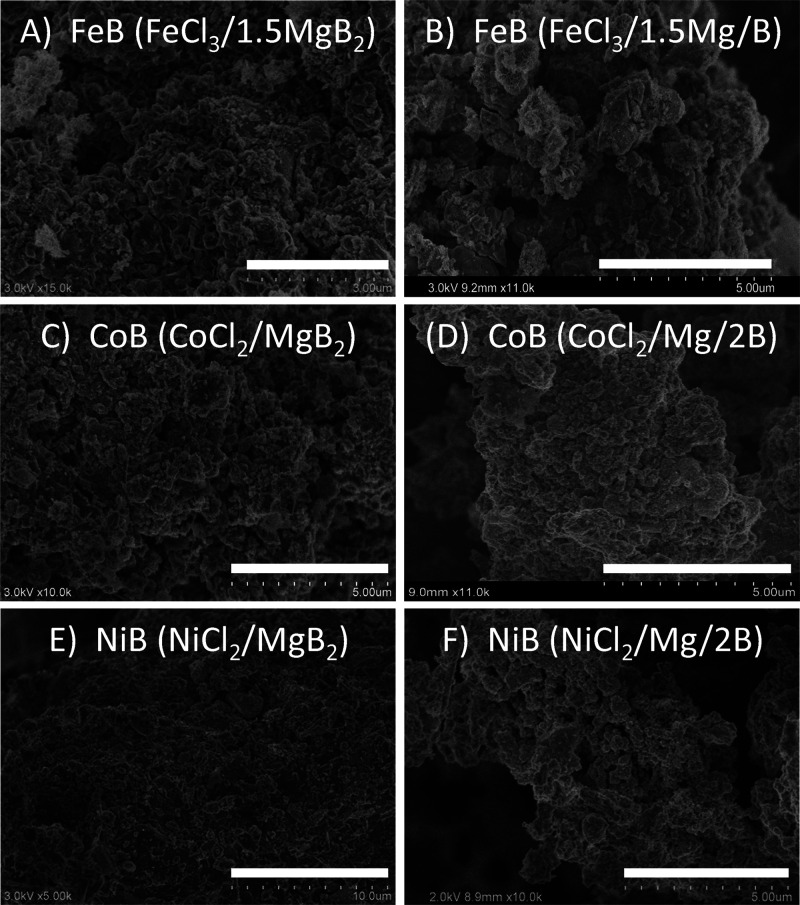
Comparison of particle morphologies obtained from MCl_*x*_ SSM reactions with MgB_2_ (left column) or Mg/B (right column). Reactions are (scale bar length noted in parentheses): (A) FeCl_3_/1.5MgB_2_ (3 μm), (B) FeCl_3_/1.5Mg/B (5 μm), (C) CoCl_2_/MgB_2_ (5 μm), (D) CoCl_2_/Mg/2B (5 μm), (E) NiCl_2_/MgB_2_ (10 μm), and (F) NiCl_2_/Mg/2B (5 μm).

Several SEM images of FeB, CoB, and NiB products are shown in Figure 3 (additional images in Figures S4 and S5). Generally, the morphologies of these three borides synthesized from MCl_*x*_ reacted with MgB_2_ or Mg/B are observed as large irregular multimicrometer sized polydisperse fused particles aggregates (∼5–80 μm) that are composed of smaller irregular plate-like or round particles with a wide size range of about 100–500 nm. The smooth surfaces of some smaller boride particles may be a result of their growth in molten MgCl_2_ and observed holes between some fused particles may be voids left by dissolved MgCl_2_ salt crystallites.

**Figure 4:**
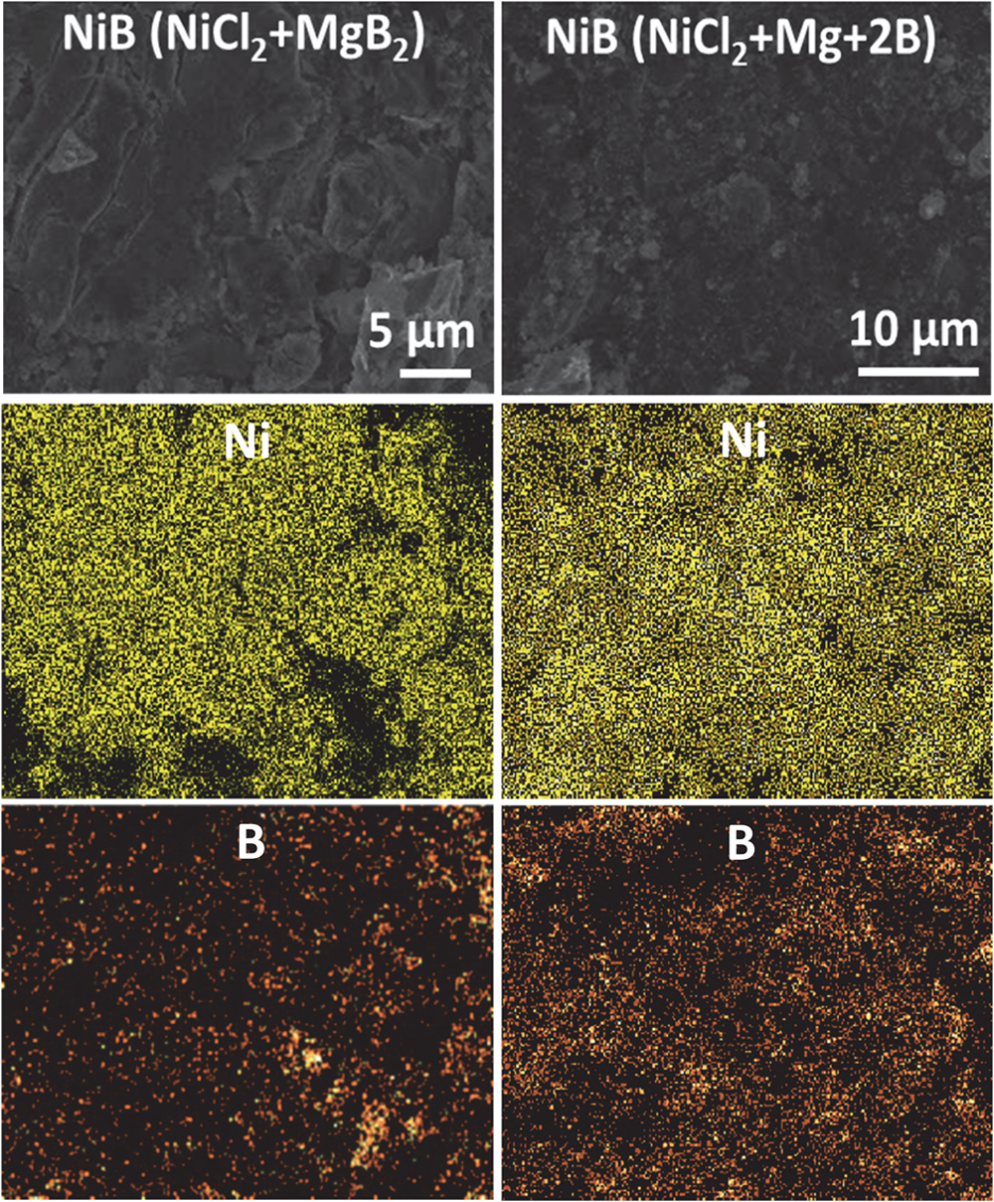
EDS maps of NiB formed from NiCl_2_ SSM reactions with MgB_2_ (left) and Mg/B (right).

The distribution of metal and boron in FeB, CoB, and NiB samples was examined using SEM-EDS mapping (Figures 4 and S6–S11). From the EDS maps, there is good overlap of metal and boron regions with some small regions where metal distribution is lower and boron content appears higher. These may be regions of excess amorphous boron in samples consistent with ICP analysis (Table 1). Boron may also be present in noncrystalline MgB_*x*_ or MgNi_3_B_2_ regions in nickel products. Magnesium is observed in most samples, consistent with low levels from ICP, and mapping places it generally in boron-rich areas (Figures S6–S11). Aside from metal, boron, and magnesium, trace amounts of oxygen and sometimes chlorine are detected that may be from the HCl wash or MgCl_2_.

**Figure 5:**
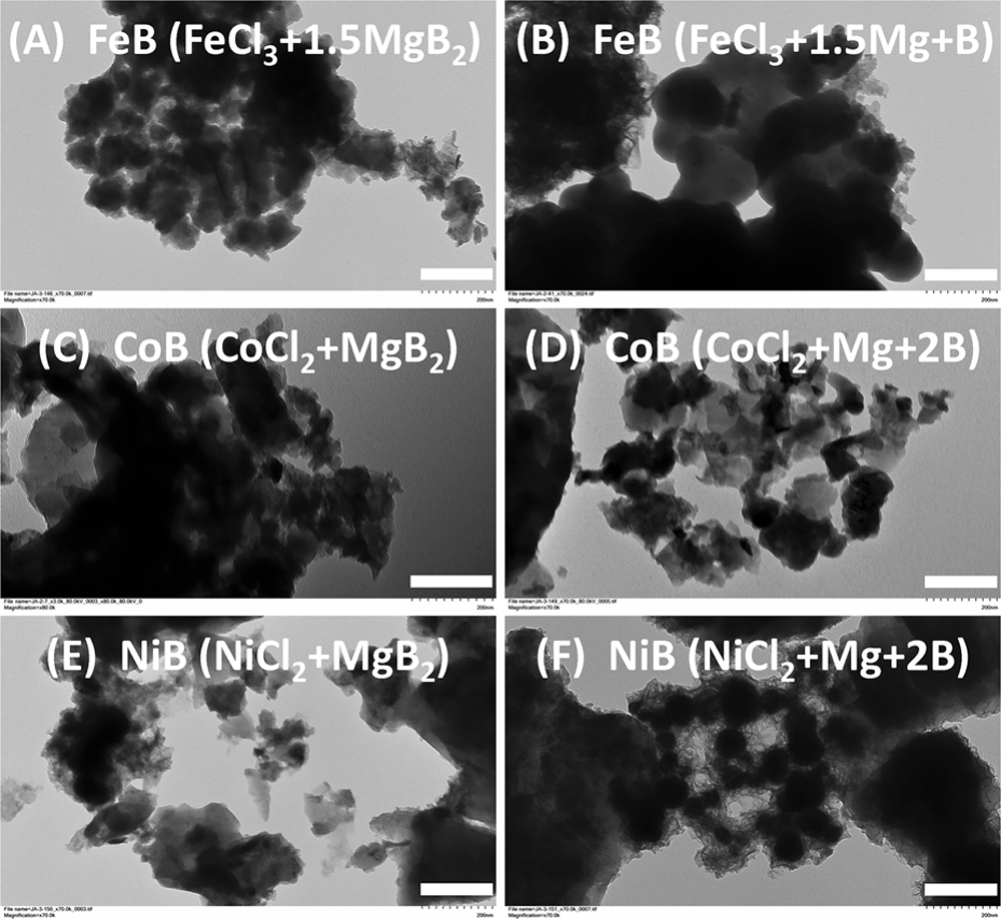
TEM images of MBs obtained from MCl_*x*_ SSM reactions with MgB_2_ (left column) and Mg/B (right column). Reactions are (A) FeCl_3_/1.5MgB_2_, (B) FeCl_3_/1.5Mg/B, (C) CoCl_2_/MgB_2_, (D) CoCl_2_/Mg/2B, (E) NiCl_2_/MgB_2_, and (F) NiCl_2_/Mg/2B. Scale bar length for images in (A)–(F) is 200 nm.

The TEM images of the smaller particle components of metal boride products show irregular agglomerated nanoparticles with sizes that are scattered over a wide range of ∼20–160 nm along with larger >200 nm particles (Figures 5 and S12). The TEM particle sizes are similar to the calculated XRD crystallite sizes of about 50 nm (Table S3). The alcohol dispersed particles sampled by TEM represent the smaller components of larger fused particle aggregates identified by SEM (Figures 3, S4, and S5). In addition to the discrete darker particles, there are semitransparent and irregular wrinkled layer-like particles in TEM images of FeB, CoB, and NiB. TEM images of amorphous boron show similar semitransparent particle shapes. Some of the thin layer-like particles may represent amorphous boron that is intermixed with the metal boride particles (Figure S12). The excess boron present in most metal boride samples is likely distributed throughout the sample in a physically separate manner and may also coat some boride particles. The TEM images include some regions where a darker particle appears partly embedded in or on the wispier weblike materials (Figures 5 and S12). Given that these metal borides form via rapid SSM reactions in molten salts, it seems less likely that conformal boron coatings grow around the borides versus producing intimate mixtures of metal boride and boron particles.

X-ray photoelectron spectroscopy (XPS) was performed on several samples to examine surface boron and metal chemical environments of washed metal boride products. Metal, B, and O regional scans for washed MgB_2_ products show that the boride surfaces have significant M–O(H) and B–O(H) bonds (Table S5 and Figure S13). Lower binding energies are expected for boron in its elemental state or bonded to metals and higher binding energies when it is partially oxidized and bonded to oxygen. The spectrum of FeB from the Mg/B reaction without excess boron is dominated by surface oxygen species for boron and iron. In addition to surface oxygen species in boron regional scans, NiB and CoB with excess boron have small surface peaks for metal borides and amorphous boron.^84^ The NiB sample also shows evidence of surface MgCl_2_ and possibly Mg–Ni–B. This XPS data shows that metal boride particles undergo surface reactions with water during the wash process and that amorphous boron may be present in most samples. This XPS surface analysis is consistent with related metal boride powder surface analysis.^37,85−87^

### Thermochemical Exothermicity and Temperature Predictions for Metal Boride SSM Reactions

Based on standard heat of formation reference data,^88^ the calculated heats of reaction for the MgB_2_ or Mg/B SSM reactions are all significantly exothermic and release sufficient reaction enthalpy (Δ*H*_rxn_) for rapid self-sustaining reactions (Tables 2 and S6). If the released enthalpy is used to heat up the products (MB, B, and MgCl_2_) without heat loss to the surroundings (adiabatic assumption), then standard phase change enthalpies and heat capacities can be used to estimate the theoretical maximum reaction temperature (*T*_ad_).^88−91^ Several reactants and products may melt, vaporize, and/or decompose during these hot SSM reactions; for example, MgB_2_ (decomp. 800 °C), Mg (mp 650 °C), and MgCl_2_ (mp 707 °C, bp 1412 °C) all undergo a phase transition before 900 °C.^88,89^ In addition, each metal halide reactant melts at low to moderate temperatures: FeCl_3_ (mp 304 °C), CoCl_2_ (mp 721 °C), and NiCl_2_ (mp 1001 °C).^88^ Elemental boron (mp 2077 °C) and the monoboride products have higher melting points than the reactants and MgCl_2_, specifically FeB (mp 1590 °C), CoB (1460 °C), and NiB (1035 °C).^88,89^

**Table 2:** Calculated Thermochemical Results for SSM Metal Boride Reactions Using MgB_2_ and Mg/B Reactants

SSM reactants	ideal products	Δ*H*_rxn_ per mol MB (kJ/mol)	adiabatic temp. (*T*_ad_)	MgCl_2_ vaporized (%)
FeCl_3_/1.5MgB_2_	FeB+2 B+1.5 MgCl_2_	–549	1412 °C	74
FeCl_3_/1.5Mg/B	FeB+1.5 MgCl_2_	–640	1590 °C	100
CoCl_2_/MgB_2_	CoB+B+MgCl_2_	–365	1412 °C	71
CoCl_2_/Mg/2B	CoB+B+MgCl_2_	–426	1412 °C	100
NiCl_2_/MgB_2_	NiB+B+MgCl_2_	–325	1412 °C	17
NiCl_2_/Mg/2B	NiB+B+MgCl_2_	–385	1412 °C	46

Regardless of the Mg or B reactant source, these SSM reactions rely heavily on the exothermic formation of the MgCl_2_ salt byproduct (Δ*H*_f_ = −644 kJ/mol) rather than the metal borides, which have Δ*H*_f_ values (kJ/mol) of −73, −94, and −46 for FeB, CoB, and NiB, respectively.^88^ The major energy difference between the comparable MgB_2_ and Mg/B reactions is that the enthalpy of reaction increases by ∼60 kJ per mol of MgB_2_ because of its heat of formation and leads to more predicted MgCl_2_ evaporation and possibly higher theoretical T_ad_ values (Table 2). In reality, the crucible absorbs some reaction heat and much of the byproduct salt remains in and around the reaction center as shown in the Figure 1 images.

**Figure 6:**
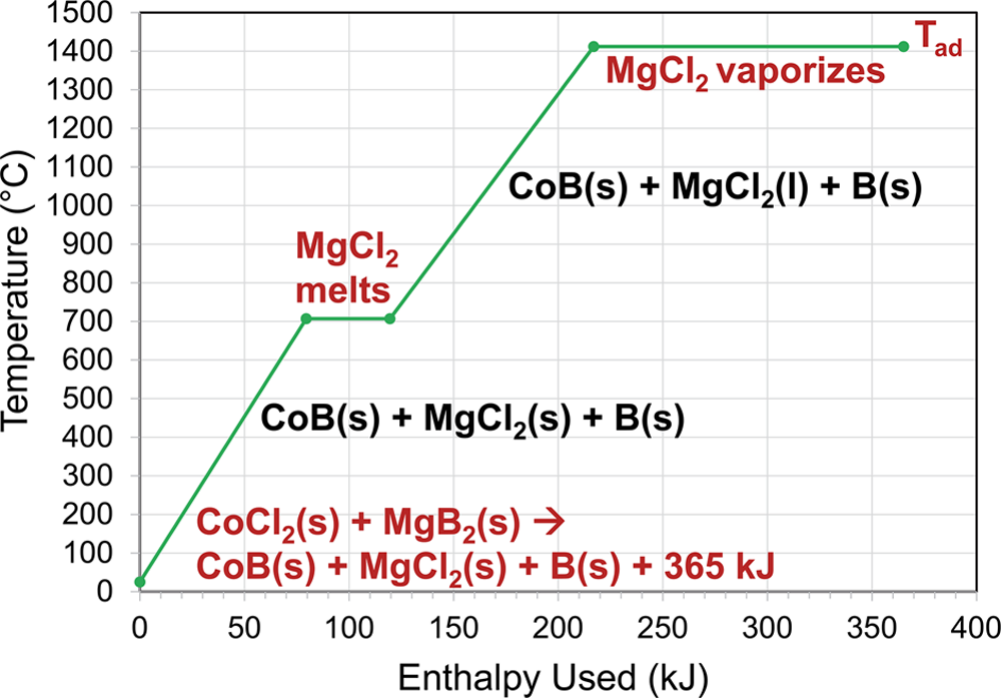
Plot of calculated adiabatic temperature data for the SSM reaction between CoCl_2_ and MgB_2_. Data points were calculated using the heat of reaction, standard heat capacities, and phase change energies. At the final data point, 71% of the MgCl_2_ is predicted to vaporize when the 365 kJ is expended.

The formation of byproduct alkaline or alkaline-earth metal halides in SSM reactions (*e.g.*, LiCl, NaCl, MgCl_2_) frequently accounts for ∼80% of the reaction exothermicity.^58^ The byproduct salt also serves as a heat sink that moderates the reaction’s maximum temperature by absorbing energy through its melting and vaporization.^59,64^ The individual enthalpy absorbing steps in an SSM reaction that are used to calculate *T*_ad_ include melting/vaporization of several products along with their heat capacities. Figure 6 shows an example of the magnitude of different enthalpy absorbing steps in the CoCl_2_/MgB_2_ SSM reaction and highlights the significance of MgCl_2_ salt phase changes (the isothermal regions) taking up enthalpy released in the reaction and limiting reaction temperatures. Additional enthalpy/temperature graphs for FeB and NiB reactions are shown in Figures S14 and S15. In most cases, the enthalpy absorbed at a given temperature for Mg/B reactions overlays with that shown for MgB_2_. This analysis makes predictions that are consistent with measured reaction temperatures in previous rapidly propagating SSM reactions.

Unlike FeB and CoB, NiB melts before MgCl_2_ boils so liquid NiB may be present in these SSM reactions and react with MgB_2_ or Mg to form the observed MgNi_3_B_2_ trace side product. The NiCl_2_/MgB_2_ SSM reaction was studied with a slight excess and deficiency (∼20%) of MgB_2_ than required for a stoichiometrically salt-balanced reaction (eq 1). With substoichiometric MgB_2_, NiB is the major phase with metal-rich minor phases (Ni_4_B_3_, Ni_2_B) and MgNi_3_B_2_ content increased slightly with excess MgB_2_ (Figure S16). These findings suggest that MgB_2_ acts as a reducing agent in addition to a boron source forming metal-rich MgNi_3_B_2_. Additional results for NiCl_2_ or CoCl_2_ SSM reactions with different Mg/B stoichiometries can be found in Table S2 and generally lower boron content leads to reduced metals and metal-rich borides.

FeB reactions with FeCl_3_ or FeCl_2_ and MgB_2_ are both highly exothermic, but the FeCl_3_ reaction has a ∼ 234 kJ/mol energetic advantage mainly due to the formation of an additional 0.5 mol of MgCl_2_ (eq 2 vs 1). Both FeCl_*x*_ rapid SSM reactions with excess boron yield single-phase and crystalline FeB (Figure S2). *T*_ad_ calculations show that FeCl_3_ and FeCl_2_ reactions with MgB_2_ both reach the boiling point of MgCl_2_ but the FeCl_3_ reaction can evaporate 31% more MgCl_2_. More information on FeCl_3_ versus FeCl_2_ SSM reactions with MgB_2_ and Mg/B can be found in Tables S4 and S6. The ability of FeCl_3_ to produce FeB using stoichiometric amounts of Mg/B may be due to its higher exothermicity leading to a longer-lived molten reaction zone, which can improve reactant/intermediate diffusion and product crystallization processes. Product crystallization and growth in these metal boride systems is complex since the FeCl_3_/MgB_2_ and CoB and NiB formation reactions have the same *T*_ad_ (1412 °C) (Table 2) but have different surface areas and particle morphologies. SEM and BET of FeB show primarily fused aggregates with moderate surface areas, while CoB and NiB contain larger monolithic blocks in addition to fused aggregates with lower surface areas. Different intermediate reactions may also be operative for the Mg/B versus MgB_2_ SSM exchange reactions as will be described later.

### Salt Dilution Effects on SSM Reactions

Salt-flux additions in SSM reactions have shown some utility for a reduction in particle size or crystallinity through dilution and lowering T_ad_ reaction temperatures. The temperature decrease and reactant dilution may be detrimental to the length of time a reaction is in a molten state and lead to incomplete or nonpropagating reactions. We investigated the effect of MgCl_2_ additions to CoB and NiB SSM reactions as shown in eqs 5 and 6 for *x* = 0.5, 1, and 2.
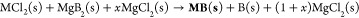
5
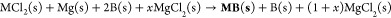
6

The XRD data shows that increasing salt dilution results in the formation of metal-rich phases and mixtures of M_*x*_B phases. The addition of MgCl_2_ to NiB reactions causes Ni_2_B to become the dominant phase, indicating that dilution interferes with Ni and B reactions in the salt flux (Table S7 and Figure S17). When sufficient MgCl_2_ is added to decrease *T*_ad_ near to its melting point (707 °C), the reaction becomes nonpropagating. The CoB formation reactions appear more tolerant to MgCl_2_ addition than NiB reactions, but Co_2_B forms at higher additions in MgB_2_ reactions, and yields are lower as *T*_ad_ drops below the MgCl_2_ boiling point (Table S8 and Figure S18). In contrast, the more exothermic CoCl_2_/Mg/2B reactions produce single-phase CoB at higher MgCl_2_ salt dilution levels. The surface areas of NiB and CoB increase significantly by about ∼4× with salt addition, likely due to the additional MgCl_2_ salt preventing particle aggregation and growth. XRD crystallite size analysis shows little or no change in the crystallite sizes upon salt addition (Table S9).

### Reaction Mechanisms and Intermediates in Metal Boride SSM Reactions

Rapid SSM reactions are expected to proceed through ionic exchange or reduction/oxidation steps that produce transient elemental intermediates.^59^ Due to the rapid self-propagation and reaction cooling, a direct study of SSM reaction mechanisms and identification of reaction intermediates is difficult.^58,92^ Prior studies suggest that transition-metal halides prefer reactions via elemental intermediates or ionic metathesis pathways, whereas lanthanides may react by ionic metathesis.^58^ SSM reactions can propagate through at least two formation energy barriers: first byproduct salt formation (*E*_a1_) followed by product formation (*E*_a2_).^58,64^ Heated filament initiation is usually sufficient to overcome the *E*_a1_ barrier to form a molten byproduct salt, and then intermediate elements or ions can diffuse and grow products (*E*_a2_).^58,59,64^ Recent SSM kinetic reaction studies show that salt byproduct formation occurs first, followed by product formation.^93−97^ The overall reaction thermochemistry influences *T*_ad_ and byproduct salt formation, and its phase transitions are important for SSM reaction propagation and product formation. SSM reactions are usually not self-propagating if the salt does not transition to a molten phase despite the overall reaction being exothermic.^59,67^

Metal boride SSM reactions with MgB_2_ (layered structure) may lead to rapid intercalation/deintercalation of Mg and metal ions as the reaction progresses. Metal boride SSM reactions that proceed via elemental (eq 7) or ionic (eq 8) intermediates are shown below. Differences in atomically mixed MgB_2_ versus macroscopically mixed Mg/B reactants may favor concerted ionic exchange with MgB_2_ and redox and elemental intermediate formation for Mg/B.
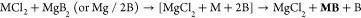
7
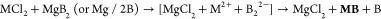
8

**Figure 7:**
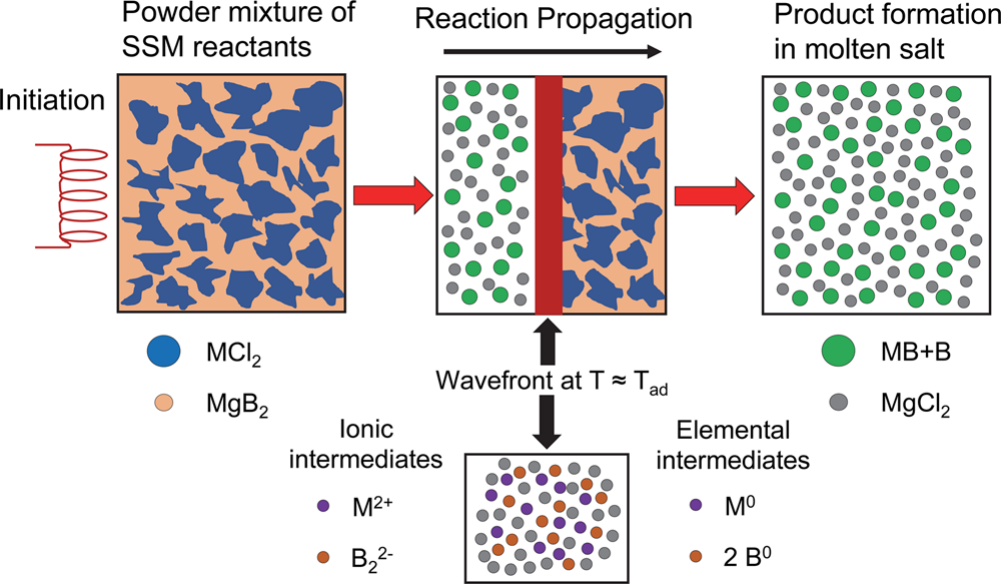
Schematic diagram of exothermic and self-propagating SSM reactions between metal halides and MgB_2_. Possible ionic or elemental intermediates and the reaction wavefront are shown. The MgB_2_ reactant can be considered as a single compound or mixed Mg/B combinations.

A schematic of the self-propagating SSM reaction of MCl_2_/MgB_2_ is shown in Figure 7 with possible transient ionic or elemental intermediates in the hot (near T_ad_) reaction zone. In the Mg/B SSM reactions described here, the hot molten reaction zone in Figure 7 likely contains metal and boron particles that can combine to form metal borides on short ∼30 s time scales.

To identify possible intermediates in the Mg/B SSM reactions, a series of exothermic “half-reactions” were conducted with MCl_2_ (M = Fe, Co, Ni) and Mg powder (eq 9). These reactions may represent the first step of metal halide reduction/salt formation prior to the reaction of the elements in the molten salt.
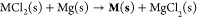
9

These reactions are very exothermic due to MgCl_2_ formation, specifically, the Δ*H*_rxn_ (kJ/mol M) values are −303, −332, and −339 for Fe, Co, and Ni formation, respectively. In contrast, subsequent M + B → MB formation reactions have much smaller Δ*H*_rxn_ values equal to the metal boride Δ*H*_f_ (−46 to −94 kJ/mol).

Hot filament initiated SSM reactions were successful in producing nanoscale powders of elemental Fe, Co, and Ni from MCl_2_ and Mg powder with ∼60% mass yields (Table S10 and Figure S19). Crystalline MgCl_2_ was identified in unwashed products of metal halide/Mg ignition reactions. These washed metal powders, which likely contain surface oxides, were vacuum-dried, combined with amorphous boron, and heated in evacuated ampules at 500 °C for several days. This yielded several metal-rich borides (*e.g.*, Co_2_B, Ni_3_B) as irregular particle aggregates with low ∼3 m^2^/g surface areas (Table S11 and Figure S20). The ∼1400 °C molten MgCl_2_ flux produced in rapid SSM reactions is likely important in overcoming solid–solid diffusion barriers to favor metal monoboride formation despite its very short time span. Metal boride formation has been reported from a furnace heated flux reaction using a combination of MCl_*x*_/Sn/B in ampules at high temperatures (700–900 °C for 4–8 h).^21,82^ In this extended heating reaction, tin serves as both a reducing agent and molten flux to facilitate the growth of metal borides. This has some parallels to the Mg/B rapid SSM reactions, where Mg reduces MCl_*x*_ and forms a molten MgCl_2_ flux for subsequent MB formation likely from elemental intermediates.

### Examination of Electrocatalytic Water Splitting with Metal Borides

#### OER Electrocatalysis Using Metal Borides

**Figure 8:**
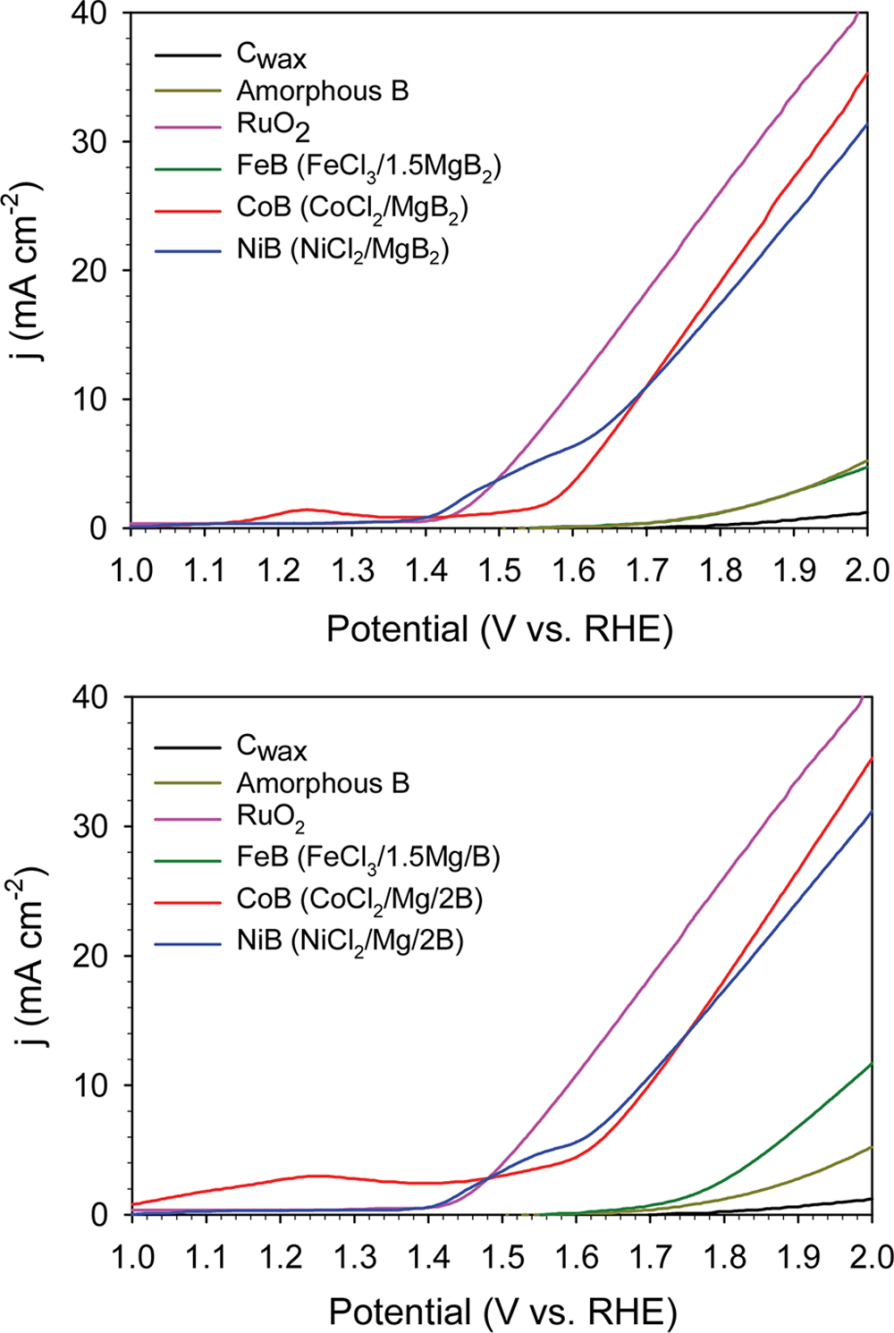
Representative OER LSV results of FeB, CoB, and NiB from SSM reactions of MCl_*x*_/MgB_2_ (top) and MCl_*x*_/Mg/B (bottom). Plots for the C_wax_ and boron are shown for comparison. Data was obtained using 0.1 M KOH in a three-electrode cell at a 5 mV/s scan rate with Hg/HgO reference and Pt wire counter electrodes. Current densities are scaled using the geometric electrode area (0.08 cm^2^).

The OER activity of FeB, CoB, and NiB formed by MgB_2_ and Mg/B SSM reactions was examined in O_2_ saturated 0.1 M KOH solutions using linear sweep voltammetry (LSV) measurements in a three electrode cell (Figure S1).^16^ The polarization data shows the bare C_wax_ electrode has negligible OER activity and amorphous boron and FeB both show low activity (Figure 8). The NiB and CoB samples from either SSM synthetic route (MgB_2_ or Mg/2B) show OER onsets and current densities that are similar to each other and at slightly higher potentials than a RuO_2_ standard. Both materials exhibit characteristic M^2+^ to M^3+^ surface oxidation peaks in the 1.1–1.6 V region prior to major current flow, which are reportedly due to formation of catalytically active MOOH surface species.^46,54,56,98,99^

A summary of averaged OER activities of metal borides from 50 LSV data sets is shown in Table 3, and LSV overlay data is shown in Figure S22. The averaged data is obtained after an initial 20 conditioning runs as initial LSVs show transient events that may be metal boride to hydroxide surface oxidation (Figure S21).^54^ The LSV data shows good overlap after the conditioning runs, with low deviations from average values. The overall OER activity of SSM synthesized metal borides is in order of FeB < NiB ≈ CoB at 10 mA/cm^2^ current density. The *iR* compensated data shows the same 10 mA/cm^2^ activity trend with values that are about 120–150 mV lower, which accounts for carbon wax and catalyst/surface resistances (Table 3). Surface charge measurements indicate that the FeB samples have larger electrochemically active surface areas (ECSA), despite showing low overall OER activity. Similar OER activities were observed for CoB and NiB using a graphite rod instead of a Pt wire counter electrode (Figure S23). The Tafel slopes for NiB are approximate and include some contribution from the overlapping surface preoxidation (NiOOH) peaks near 1.4 eV. The Tafel slopes for the three metal borides range from ∼43 to 209 mV/decade. FeB has the highest Tafel slope, consistent with its very low OER activity, which may reflect surface oxidation and surface particle adhesion to the electrode. Literature data in Tables S12 and S13 for crystalline and amorphous Fe–B, Co–B, and Ni–B show Tafel slopes over a wide range from ∼50 to 200 mV/decade. As noted above, the as-synthesized MB materials show significant evidence of surface M–O and B–O species in the washed products. These coatings may influence their surface electrocatalytic activity. The analysis of the kinetics of water oxidation catalysis may be influenced by the electrolyte used and the level of *iR* compensation.^100^

**Table 3:** Summary of OER Electrocatalysis with SSM Synthesized Metal Borides

sample	applied potential @ 10mA/cm^2^ (mV)^a^	applied potential @ 20mA/cm^2^ (mV)^a^	Tafel (mV/decade)	ECSA (cm^2^)
RuO_2_	1591 ± 2 (1521 ± 2)	1725 ± 4 (1563 ± 3)	51	223
FeB (FeCl_3_/1.5MgB_2_)	n/a	n/a	209	41
FeB (FeCl_3_/1.5Mg/B)	1964 ± 2	n/a	171	55
CoB (CoCl_2_/MgB_2_)	1687 ± 2	1811 ± 2	63	3
CoB (CoCl_2_/Mg/2B)	1699 ± 2 (1570 ± 2)	1823 ± 2 (1606 ± 3)	65	3
NiB (NiCl_2_/MgB_2_)	1685 ± 2	1840 ± 3	43^b^	3
NiB (NiCl_2_/Mg/2B)	1688 ± 2 (1521 ± 2)	1838 ± 2 (1598 ± 1)	49^b^	1

a Applied potentials reported versus RHE in 0.1 M KOH and current densities normalized to geometric electrode area of 0.08 cm^2^ (85% *iR* compensation results in parentheses). Deviations are for 50 LSV runs or 10 LSV runs at 85% *iR* compensation. n/a means current density not achieved or not analyzed.

b Approximate values due to preoxidation peak overlap with OER onset.

A similar OER activity trend is observed when these metal borides were examined in a more strongly basic 1.0 M KOH environment, with CoB and NiB showing similar activity and all samples showing 10 mA/cm^2^ current densities at nearly 100 mV lower applied potentials versus in 0.1 M KOH (Table S14). As above, 20 conditioning runs were performed in 1.0 M KOH before running the 50 LSVs used to calculate averages (Figure S24). Graphical representations of current densities achieved for subsequent LSV runs show some samples may have materials stability issues at these higher base concentrations with slow movement to lower or higher applied potentials upon subsequent scans (Figure S25).

The extended 18 h OER activity of CoB and NiB samples was examined in 0.1 M KOH using constant potential chronoamperometry with a set potential to maintain ∼10 mA/cm^2^ current density and they show very good overall stability (Figure S26). The powders embedded on carbon wax tips can be cut off and analyzed after this 18 h experiment. Powder XRD of NiB and CoB on C_wax_ electrode tips before and after the 18 h chronoamperometry experiment shows clear evidence for retention of crystalline CoB and NiB on the electrode tip after extended oxidizing electrochemistry (Figure S27). Post-chronoamperometry EDS elemental maps of CoB and NiB on the carbon wax tip show that the metal and B distribution on the catalyst surface is still present after electrochemistry measurements (Figures S28–S31). The embedded particles contain metal and boron as well as oxygen and trace magnesium. While the bulk metal boride structures survive the catalytic reactions, it is likely that the surface of these catalysts contains some of its initial M–OH, B–OH, and M–B–OH species. NiB and CoB samples include excess boron that on its own shows no OER activity, but there are reports of enhanced OER activity from metal borides with boron components that provide stability and performance enhancements from borate surface modification.^56,101^

When considering the OER activities relative to an ideal 1.23 V potential, CoB and NiB show 10 mA/cm^2^ current density in the overpotential range (85% *iR* compensation) of 290–350 mV in 0.1 M KOH and 1.0 M KOH. The SSM synthesized crystalline FeB, CoB, and NiB show OER activities that are similar to those from prior studies with related crystalline metal borides (Tables S12 and S13). It is challenging to compare results for our crystalline micrometer-sized metal borides to prior reports on amorphous borides or nanoscale structures grown on supports, but amorphous materials generally require lower overpotentials to achieve higher current densities. Amorphous FeB^49,53,102^ and other Fe–B compositions^18^ report moderate electrocatalytic OER activity in 1.0 M KOH (∼300–400 mV overpotential), which is similar to our crystalline FeB in the same electrolyte. Supported metal borides, such as CoB on carbon paper,^35^ require overpotentials near 340 mV for 10 mA/cm^2^ current densities in 1.0 M KOH that are similar to our CoB results (347 mV).

#### HER Electrocatalysis with Metal Borides

**Figure 9:**
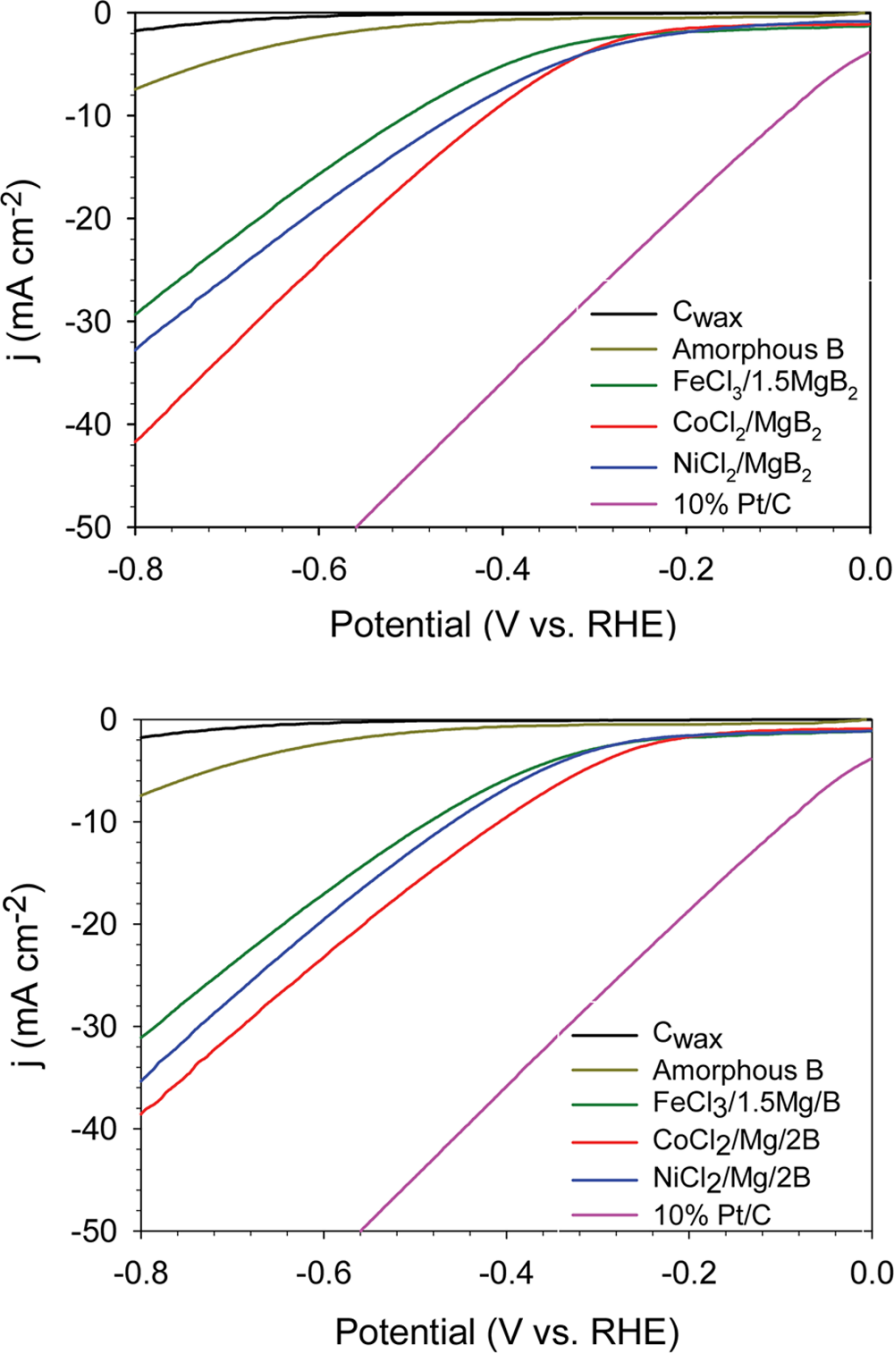
Representative HER LSV results of FeB, CoB, and NiB from SSM reactions of MCl_*x*_/MgB_2_ (top) and MCl_*x*_/Mg/B (bottom). Plots for the C_wax_ and amorphous B are shown for comparison. Data was obtained using 0.1 M KOH in a three-electrode cell at 5 mV/s scan rate with Hg/HgO reference and Pt wire counter electrodes. Current densities are scaled using the geometric electrode area (0.08 cm^2^).

The HER activities of FeB, CoB, and NiB formed by MgB_2_ and Mg/B SSM reactions were examined in H_2_ saturated 0.1 M KOH. All three crystalline borides produced by either SSM reaction showed reproducible and stable hydrogen evolution activity as summarized in Table 4 (see Figure 9 for overlay LSVs and full LSV data sets in Figure S32). The M/B samples were also examined with *iR* compensation that lowers the net applied potential for 10 mA/cm^2^ current densities by ∼120 mV. The overall HER activity of these monoborides is ordered as FeB < NiB < CoB and is in a potential range of −300 to −400 mV (*iR* compensated) to achieve 10 mA/cm^2^ current densities. Similar HER activities were observed for three metal borides when measurements were taken with graphite counter electrodes instead of a Pt counter electrode (Figure S23). The Tafel slopes for metal borides are in 130–215 mV/decade range and are larger than that for the Pt/C standard (114 mV/decade) and may reflect some surface oxides or carbon wax interface issues that are the subject of our ongoing work.

**Table 4:** Summary of HER Electrocatalysis with SSM Synthesized Metal Borides in 0.1 M KOH

sample	(η_**10**_) (mV)^a^	(η_20_) (mV)	Tafel (mV/decade)
10%Pt/C	–93 ± 6 (−37 ± 1)	–215 ± 6 (−92 ± 2)	114
FeB (FeCl_3_/1.5MgB_2_)	–507 ± 7	–669 ± 6	215
FeB (FeCl_3_/1.5Mg/B)	–487 ± 10 (−375 ± 7)	–645 ± 9 (−435 ± 2)	204
CoB (CoCl_2_/MgB_2_)	–416 ± 2	–548 ± 2	132
CoB (CoCl_2_/Mg/2B)	–410 ± 4 (−315 ± 5)	–558 ± 4 (−376 ± 5)	150
NiB (NiCl_2_/MgB_2_)	–448 ± 14	–614 ± 11	174
NiB (NiCl_2_/Mg/2B)	–457 ± 9 (−329 ± 1)	–604 ± 10 (−389 ± 2)	166

a overpotentials reported versus RHE in 0.1 M KOH and current densities normalized to geometric electrode area of 0.08 cm^2^ (85% *iR* compensation results in parentheses). Deviations are for 50 LSV runs or 10 LSV runs at 85% *iR* compensation. n/a means not analyzed.

The extended HER activity of FeB, CoB, and NiB in 0.1 M KOH was examined using constant potential chronoamperometry over an 18 h period with a potential required to maintain ∼10 mA/cm^2^ current density. All samples showed relatively stable activity (Figure S33), though a small improvement in current density was observed for FeB. A similar improvement in FeB activity and stability in 0.1 M KOH was observed when a graphite counter electrode was used instead of the platinum wire, so these activity changes are most likely due to FeB partial decomposition (Figure 34). All six samples after the 18 h HER chronoamperometry experiment still show XRD peaks for crystalline CoB, NiB, and (less) FeB embedded on the carbon wax electrode tip (Figure S35). These XRD results support the retention of bulk crystalline borides in a reducing electrochemical environment. The lower crystallinity of FeB is consistent with some degradation of this boride during catalysis. EDS elemental mapping of FeB, CoB, and NiB shows that metal and boron are still distributed uniformly on the catalyst particle surface overlaid with oxygen and some carbon wax is visible after the 18-h experiment (Figures S36–S40).

When changing electrolytes to either 1.0 M KOH or 0.5 M H_2_SO_4_, current densities were achieved at ∼130 mV lower overpotentials (Tables S15 and S16, Figures S41 and S42), but some samples exhibited a larger spread in the LSV curves, indicating some material instability. The 85% *iR* compensated 10 mA/cm^2^ current densities for these metal borides are in the −240 to −280 mV range in 1.0 M KOH and −260 to −325 mV range in 0.5 M H_2_SO_4_. The ECSA values in acid are similar to those in base (Table 3 and S16). The SSM synthesized metal borides have Tafel slopes that are in a similar range (∼100–150 mV/decade) as literature reports on a variety of other metal borides (∼70–190 mV/decade), but as noted earlier, variations in these slopes may be due to both electrolyte, carbon wax interface, and *iR* compensation differences. A comparison of LSV data for the metal borides in three electrolytes (0.1 M KOH, 1.0 M KOH, and 0.5 M H_2_SO_4_) with and without *iR* compensation are shown in Figure S43 to demonstrate how corrections for carbon wax and interfacial resistance influences the applied potentials. While *iR* compensation does improve the data, there are recent articles detailing *iR* compensation considerations.^77,78^ We continue to investigate if the carbon wax electrode design may be improved to further limit *iR* compensation issues.

**Figure 10:**
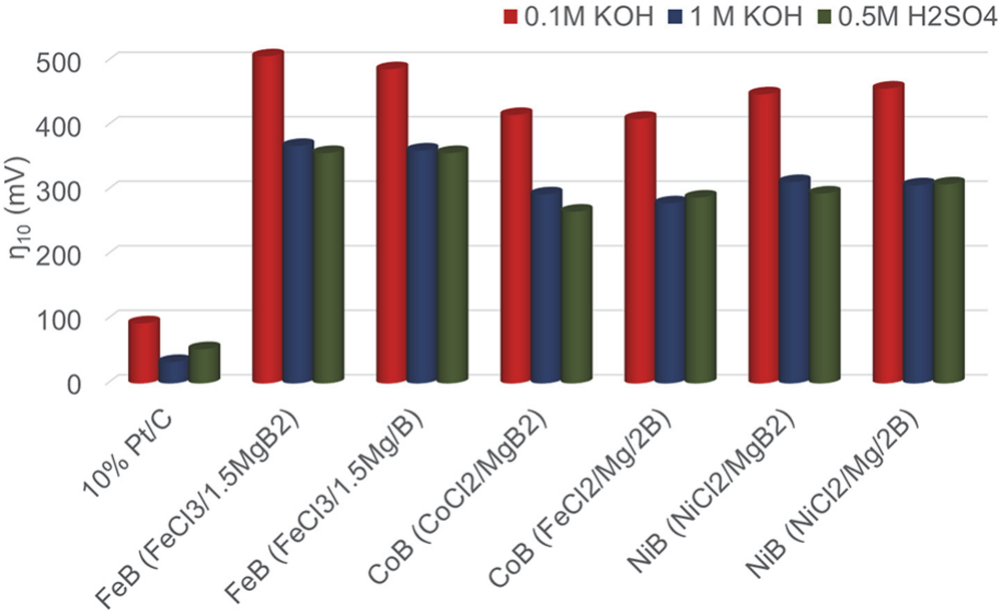
Comparison of HER applied potentials (*iR* uncompensated) needed for 10 mA/cm^2^ current density in 0.1 M KOH, 1.0 M KOH, and 0.5 M H_2_SO_4_ for FeB, CoB, and NiB catalysts synthesized with either MgB_2_ or Mg/B and embedded on the surface of carbon wax electrodes. 85% *iR* compensation reduces applied potentials by ∼20–120 mV depending on electrolyte (see text).

The HER results from the crystalline FeB, CoB, and NiB are comparable to or less active than those of related amorphous or crystalline metal borides previously reported as electrocatalysts (Tables S12 and S13). We did not find previous literature reports for HER activity of crystalline free-standing powders of FeB CoB, or NiB to compare to the HER activity of the SSM synthesized metal borides. Previous HER studies of *amorphous* CoB and NiB (some grown on supports) report 10 mA/cm^2^ current densities from −100 to −200 mV applied potentials in different electrolytes.^40,48,103,104^ A bar chart comparison of applied potentials to achieve 10 mA/cm^2^ current density for the SSM synthesized metal borides and analyzed on carbon wax electrodes is shown in Figure 10.

This chart highlights both similarities in applied potentials for different preparations of the same boride (MgB_2_ versus Mg/B reactants) and shows higher base or acid electrolyte concentration leading to lower applied potentials to achieve 10 mA/cm^2^ current density, though, as noted above, the higher concentration acid or base electrolytes appear to impact the stability of the materials. From XPS surface analysis, the formation of M–O and B–O surface bonds are present in as-synthesized metal borides. Gas phase bond energies (kJ/mol) indicate that M–O (∼400) and B–O (∼800) bonds are much stronger than M–H or B–H bonds (∼300).^105^ In reducing acidic environments, BH_3_ formation and loss from the boride surface may occur for some borides. Previous catalytic and theoretical studies on MoB_2_^21^ and other boron-rich diborides^70^ show intriguing theoretical predictions for the free energy of hydrogen adsorption energy for different metal boride crystal planes and surface atoms on the particle surface (M versus B–B chains). For MoB_2_, theory indicates that boron-rich surfaces are more favorable for weaker B–H bonding and important to its HER activity. In water oxidation reactions, the surface metals and their bonding to OH_2_ is a key intermediate step. In the case of the metal monoborides studied here, somewhat larger Tafel slopes than the MB_2_ electrocatalysts may indicate sluggish kinetic steps with the surface structures of the monoborides, possibly due to surface-bound intermediates or M/B–O oxidation layers. There may also be some influence of the powder to carbon wax interface that we are examining in more detail. Additional spectroscopic analysis of metal boride catalyst particle surfaces after HER and OER is also being pursued to better identify changes to surface bonding after electrocatalysis.

## Conclusions

Rapid and exothermic SSM reactions were successfully utilized to synthesize thermodynamically stable polycrystalline FeB, CoB, and NiB in seconds. Both single (MgB_2_) and double (Mg and B) reactants were used with anhydrous metal chlorides to produce these metal borides with good yields. These rapid SSM reactions add to the metal boride materials synthesis toolbox. Thermochemical analyses identified characteristics that favor rapid ignition-based SSM metal boride syntheses, such as precursor combinations with high reaction exothermicity (∼300 kJ/mol) and the ability to sustain a molten magnesium chloride salt environment near 1400 °C. The salt addition/dilution of these SSM reactions interferes with product formation despite some improvement in surface area. Evidence shows that intermediate metal particle formation can occur in the reaction zone of these rapid SSM reactions. These metal monoborides show moderate activity in HER and OER electrocatalysis similar to related crystalline and amorphous borides. NiB and CoB show appreciable activity in both reactions, while FeB shows primarily HER activity. The overall OER activities of metal borides are in order of FeB ≪ NiB ≈ CoB, while relative HER activities are FeB < NiB < CoB. In 0.1 M KOH, the crystalline metal borides show relatively stable activity under sustained HER and OER over 18 h time periods and post-electrochemistry XRD structure analysis reveals clear retention of crystalline bulk metal boride structures. This study demonstrates a new flexibility and tunability for rapid SSM reactions producing chemically robust metal monoborides using Mg/B mixed reactants. This SSM reaction strategy may find useful extensions to other metal boride syntheses and to other metal compounds with nonvolatile main group elements such as using Mg/X reactant combinations where X is C, Al, or Si.

## Supporting Information Available

Additional XRD results for SSM products and half reactions, tables summarizing results for different SSM reaction stoichiometries, additional product SEM and TEM images, thermochemical reaction calculations and graphs for FeB and NiB, graphical and tabular results for electrocatalytic HER and OER studies, and tables of literature comparison data, images of electrochemical cell, EDS maps of borides before and after 18-h electrocatalysis, XPS regional scans of select metal borides, additional LSV overlay graphs and summary tables for OER and HER in different electrolytes, XRD data on borides on carbon wax tips after 18-h electrocatalysis, selected comparisons using graphite counter electrodes and *iR* compensation (PDF)
