# Supplementary material for: Rapid and Energetic Solid-State Metathesis Reactions for Iron, Cobalt, and Nickel Boride Formation and Their Investigation as Bifunctional Water Splitting Electrocatalysts
Source: ACS Mater Au. 2022 Apr 21;2(4):489–504. doi: 10.1021/acsmaterialsau.1c00079 (PMC9295309; doi:10.1021/acsmaterialsau.1c00079)
Supplement: Supplementary file 1 — mg1c00079_si_001.pdf [file mg1c00079_si_001.pdf]

Supporting Information for

Rapid and energetic solid-state metathesis reactions for iron, cobalt, and nickel boride formation and their investigation as bifunctional water splitting electrocatalysts

Janaka P. Abeysinghe, Anna F. Kölln, Edward G. Gillan*
Department of Chemistry, University of Iowa, Iowa City, Iowa 52242
E-mail: edward-gillan@uiowa.edu

Tables and Figures are ordered as they are first referenced in the main text of the paper.

List of Experimental, Tabular, and Graphical Supporting Information

Figure S1. Images of electrochemical cell, electrode tips, and tip XRD geometries.
Table S1. Summary table of reference electrode calibration and electrolyte pH results.
Table S2. Co-B and Ni-B product formation from SSM reactions with different stoichiometries.
Table S3. XRD crystallite size results for metal borides.
Figure S2. XRD results for FeB formation from different stoichiometries of $FeCl_3$ and $FeCl_2$ reactions.
Table S4. Fe-B product formation from SSM reactions for different reactants and stoichiometries.
Figure S3. Structural comparisons of FeB, CoB, and NiB showing M-M and B-B frameworks.
Figure S4. Additional SEM images of MCl_x/MgB_2 reaction products.
Figure S5. Additional SEM images of MCl_x/Mg/B reaction products.
Figure S6. EDS maps of FeB powder formed from $FeCl_3$+1.5MgB_2 reaction.
Figure S7. EDS maps of FeB powder formed from $FeCl_3$+1.5Mg+B reaction.
Figure S8. EDS maps of CoB powder formed from $CoCl_2$+MgB_2 reaction.
Figure S9. EDS maps of CoB powder formed from $CoCl_2$+Mg+2B reaction.
Figure S10. EDS maps of NiB powder formed from $NiCl_2$+MgB_2 reaction.
Figure S11. EDS maps of NiB powder formed from $NiCl_2$+Mg+2B reaction.
Figure S12. Additional TEM images of metal borides and amorphous B.
Table S5. Summary of XPS data on select metal borides
Figure S13. X-ray photoelectron spectroscopy (XPS) regional scans for FeB, CoB, and NiB.
Table S6. Thermochemical data of the SSM reactions for the formation of FeB, CoB, and NiB.
Figure S14. T_{ad} graph for FeB SSM reaction between $FeCl_3$ and MgB_2.
Figure S15. T_{ad} graph for NiB SSM reaction between $NiCl_2$ and MgB_2.
Figure S16. XRD results for $NiCl_2/MgB_2$ reactions run above and below salt balanced stoichiometries.
Table S7. Salt dilution results of $NiCl_2$+MgB_2 and $NiCl_2$+Mg+2B reactions.
Figure S17. XRD results of $NiCl_2/MgB_2/MgCl_2$ and $NiCl_2$/Mg/2B/$MgCl_2$ salt dilution reactions.
Table S8. Salt dilution results of $CoCl_2$+MgB_2 and $CoCl_2$+Mg+2B reactions.
Figure S18. XRD results of $CoCl_2/MgB_2/MgCl_2$ and $CoCl_2$/Mg/2B/$MgCl_2$ salt dilution reactions.
Table S9. The crystallite size decrease of NiB and CoB with salt dilution.
Table S10. Results from MCl_2/Mg SSM reactions.
Figure S19. Powder XRD results of the MCl_2/Mg reactions.
Table S11. Results from M/B ampoule reactions.
Figure S20. SEM images of M/B ampoule reaction products.
Figure S21. Overlays of first 20 conditioning LSV runs for OER experiments in 0.1 M KOH.
Figure S22. OER LSV overlay and average plots for FeB, CoB, and NiB in 0.1 M KOH.
Figure S23. Comparison of OER and HER activity of MBs using a Pt and a graphite counter electrode.
Table S12. Literature comparison table for the OER and HER activities of crystalline MB.
Table S13. Literature comparison table for the OER and HER activities of amorphous MB.
Table S14. OER summary for FeB, CoB, and NiB in 1.0 M KOH.

Figure S24. Overlays of first 20 conditioning LSV runs for OER experiments in 1.0 M KOH.
Figure S25. OER LSV overlay and average plots for FeB, CoB, and NiB in 1.0 M KOH.
Figure S26. Applied positive potential chronoamperometry OER measurements of MBs in 0.1 M KOH.
Figure S27. Powder XRD results of pre- and post-positive potential OER chronoamperometry of CoB and NiB samples formed by MCl_2+MgB_2 and MCl_2+Mg+2B reactions.
Figure S28. Post-positive potential OER chronoamperometry EDS maps of CoB formed from $CoCl_2$+MgB_2 reaction.
Figure S29. Post-positive potential OER chronoamperometry EDS maps of CoB formed from $CoCl_2$+Mg+2B reaction.
Figure S30. Post-positive potential OER chronoamperometry EDS maps of NiB formed from $NiCl_2$+MgB_2 reaction.
Figure S31. Post-positive potential OER chronoamperometry EDS maps of NiB formed from $NiCl_2$+Mg+2B reaction.
Figure S32. Overlay and average plots of 50 LSV scans for metal boride HER in 0.1 M KOH.
Figure S33. Applied negative potential chronoamperometry HER measurements of MBs in 0.1 M KOH.
Figure S34. HER activity and stability of FeB in 0.1 M KOH with graphite versus platinum wire counter electrode.
Figure S35. Post-negative potential HER chronoamperometry XRD results on electrode tips.
Figure S36. Post-negative potential HER chronoamperometry EDS maps of FeB formed from $FeCl_3$+1.5MgB_2 reaction.
Figure S37. Post-negative potential HER chronoamperometry EDS maps of FeB formed from $FeCl_3$+1.5Mg+B reaction.
Figure S38. Post-negative potential HER chronoamperometry EDS maps of CoB formed from $CoCl_2$+MgB_2 reaction.
Figure S39. Post-negative potential HER chronoamperometry EDS maps of CoB formed from $CoCl_2$+Mg+2B reaction.
Figure S40. Post-negative potential HER chronoamperometry EDS maps of NiB formed from $NiCl_2$+Mg+2B reaction.
Table S15. Summary of HER results for FeB, CoB, and NiB in 1.0 M KOH.
Figure S41. HER LSV overlay and average plots for FeB, CoB, and NiB in 1.0 M KOH.
Table S16. Summary of HER results for FeB, CoB, and NiB in 0.5 M H_2SO_4.
Figure S42. HER LSV overlay and average plots for FeB, CoB, and NiB in 0.5 M H_2SO_4.
Figure S43. HER and OER LSV overlay plots for metal borides from Mg/B reactions with and without 85% iR compensation.

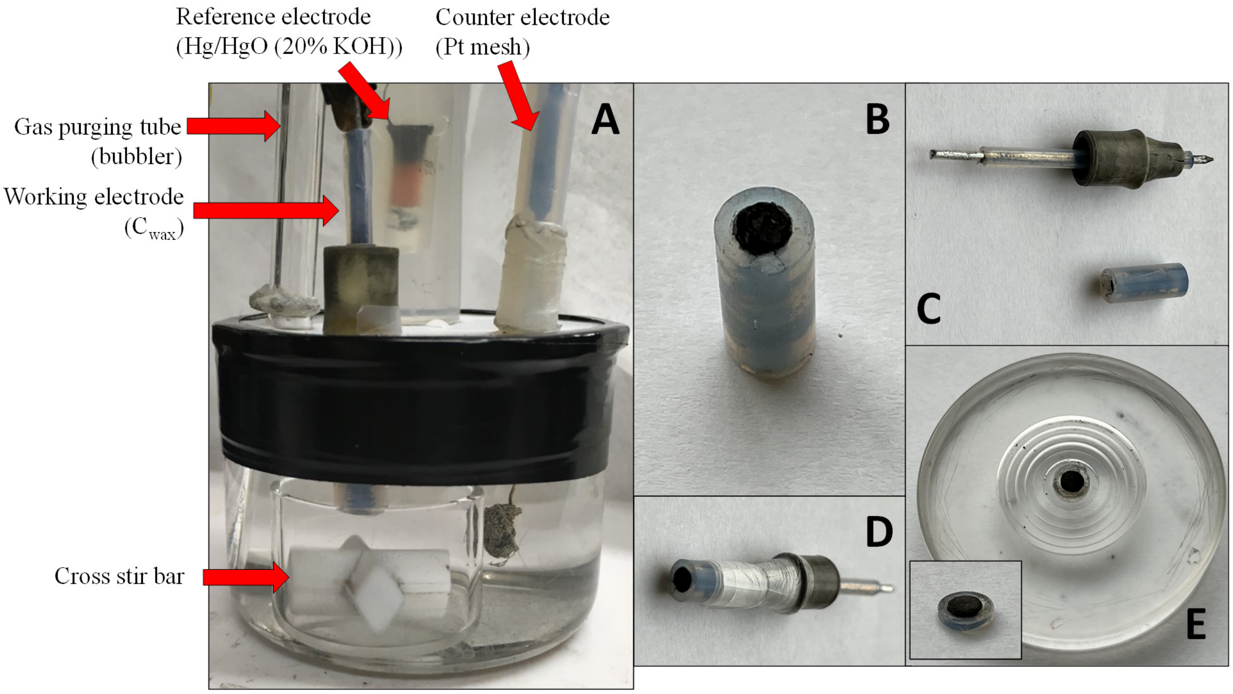

Figure S1. Images of electrochemical cell and electrode tip. (A) Three electrode electrochemical cell (working electrode-C_{wax}, counter electrode-Pt mesh, reference electrode- Hg/HgO (20% KOH), bubbler, and cross stir bar, (B) C_{wax} electrode tip with MB powder on it, (C) disassembled C_{wax} electrode tip+Al rod connector, (D) assembled C_{wax} electrode tip+Al rod connector, (E) cut tip slide that was used for post-electrochemistry EDS analysis (inset image) and cut tip slice on XRD sample holder for post-electrochemistry XRD analysis.

Table S1. Summary table of reference electrode calibration and electrolyte pH results.

Electrolyte	Theoretical pH	Measured pH	Theoretical potential (V)	Measured potential (V)[a]	Theoretical Potential for measured pH value
0.1 M KOH	13	13.0	-0.865	-0.855	-0.862
1.0 M KOH	14	13.9	-0.924	-0.918	-0.918
0.5 M H_2SO_4	0.3	0.4[b]	-0.259	-0.258	-0.262

a) The reference calibration was conducted in standard three-electrode system with Pt wire as the working and Pt mesh as counter electrodes, and the Hg/HgO or Hg/Hg_2Cl_2 electrode as the reference electrode. The electrolytes were purged and saturated with H_2 (for 0.5 M H_2SO_4, 0.1 M, and 1.0 M KOH) for 30 min before calibration and CVs were obtained at a scan rate of 1 mV/s. Measured potentials are obtained from reduction current onsets at working electrode. b) The 0.5 M H_2SO_4 solution was diluted to 0.05 M H_2SO_4 (theoretical pH of 1.23 using K_a for HSO_4^- of 0.012) and measured using a calibrated pH meter. The measured pH of 1.28 corresponds to 0.044 M H_2SO_4. The extrapolated pH for a 0.44 M H_2SO_4 solution was then calculated as 0.35.

Table S2. Co-B and Ni-B product formation from SSM reactions for different reactants and stoichiometries.

Reaction	Target	% yield based MB_x/B product	XRD (major phase in bold)
$CoCl_2/MgB_2$	Co:2B	80	**CoB**
$CoCl_2$/Mg/2B	Co:2B	85	**CoB**
$CoCl_2$/Mg/B	Co:B	46	**CoB,** Co
$NiCl_2/MgB_2$	Ni:2B	81	**NiB,** *m*-Ni_4B_3, $MgNi_3B_2$
$NiCl_2$/Mg/2B	Ni:2B	86	**NiB,** $MgNi_3B_2$
$NiCl_2$/Mg/B	Ni:B	78	**$MgNi_3B_2$,** Ni_2B, NiB, *m*-Ni_4B_3
$2NiCl_2$/2Mg/B	Ni:0.5B	64	**$MgNi_3B_2$,** Ni_2B, NiB, *m*-Ni_4B_3

Table S3. The crystallite sizes of metal borides determined by X-ray line broadening.

**Metal boride**	$FeCl_3$+ MgB_2	$FeCl_3$+ Mg+B	$CoCl_2$+ MgB_2	$CoCl_2$+ Mg+2B	$NiCl_2$+ MgB_2	$NiCl_2$+ Mg+2B
**Crystallite size (nm)**	51	53	39	41	38	38

The (220) XRD peak at 47.3° 2θ of Si powder was used for instrument broadening and the (111) peaks of metal borides (FeB 41.3°, CoB 41.2°, and NiB 45.2°) were used for the calculations. Values are the average of two sample scan results calculated using Scherrer and Warren equations.

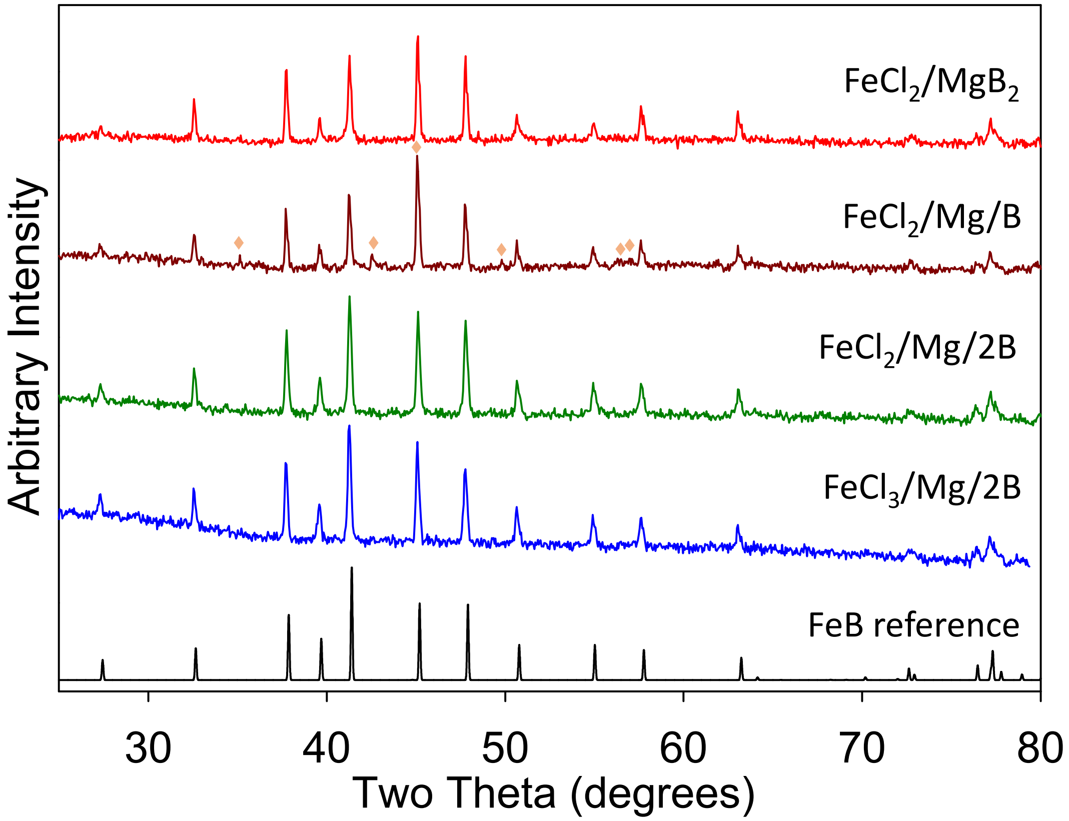

Figure S2. XRD results for FeB formation from different reactions of $FeCl_3$ or $FeCl_2$ with MgB_2 or Mg/B. The diamond symbols identify peaks for tetragonal Fe_2B.

Table S4. Fe-B product formation from SSM reactions for different precursors and reactant stoichiometries.

Reaction	M:B ratio	% yield based on MB_x/B product	XRD (major phase in bold)
$FeCl_3$/1.5MgB_2	Fe:2B	82	**FeB**
$FeCl_3$/1.5Mg/2B	Fe:2B	82	**FeB**
$FeCl_3$/1.5Mg/B	FeB	73	**FeB**, Fe_2B
$FeCl_2$/MgB_2	Fe:2B	65	**FeB**
$FeCl_2$/Mg/2B	Fe:2B	91	**FeB**
$FeCl_2$/Mg/B	Fe:B	97	**FeB,** Fe_2B
4$FeCl_2$/MgB_2	Fe:0.5B	11	**unidentified**
$FeCl_3$/1.5Mg/4B	Fe:4B	118 (Mg in product)	**FeB,** unidentified

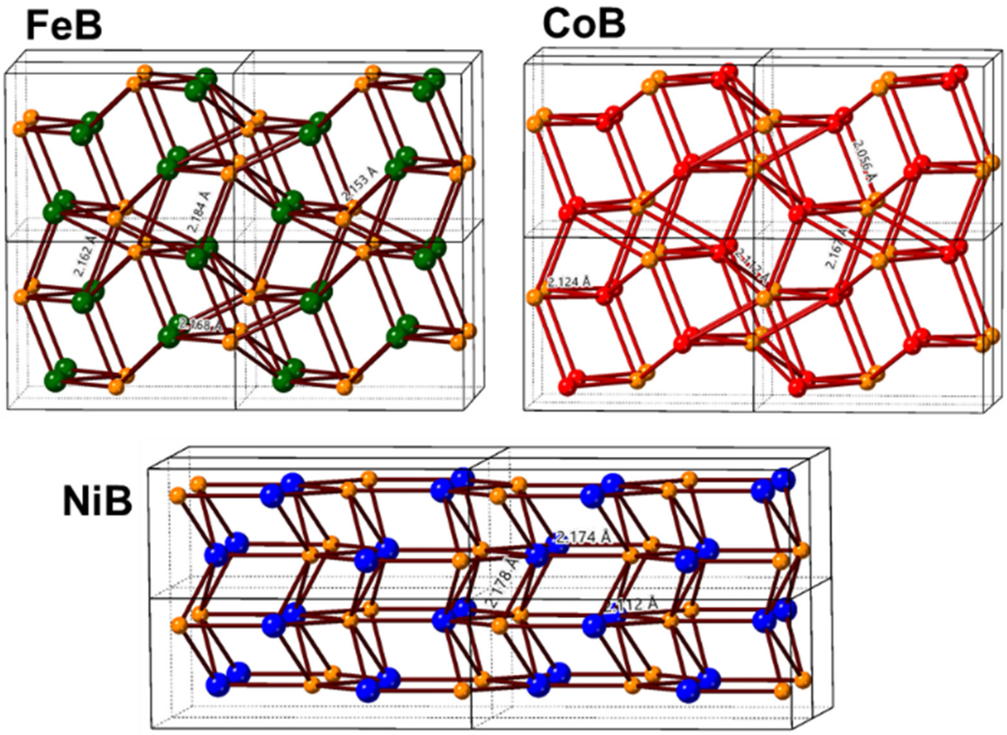

Figure S3A. Structural representations of orthorhombic FeB, CoB, and NiB showing M-B coordination in each structure (Fe-green, Co-red, Ni-blue, and B-orange). These metal borides are all orthorhombic but crystallize in several different space groups [FeB - Pnma (62), CoB - Pbnm (62), NiB- Cmcm (63)].

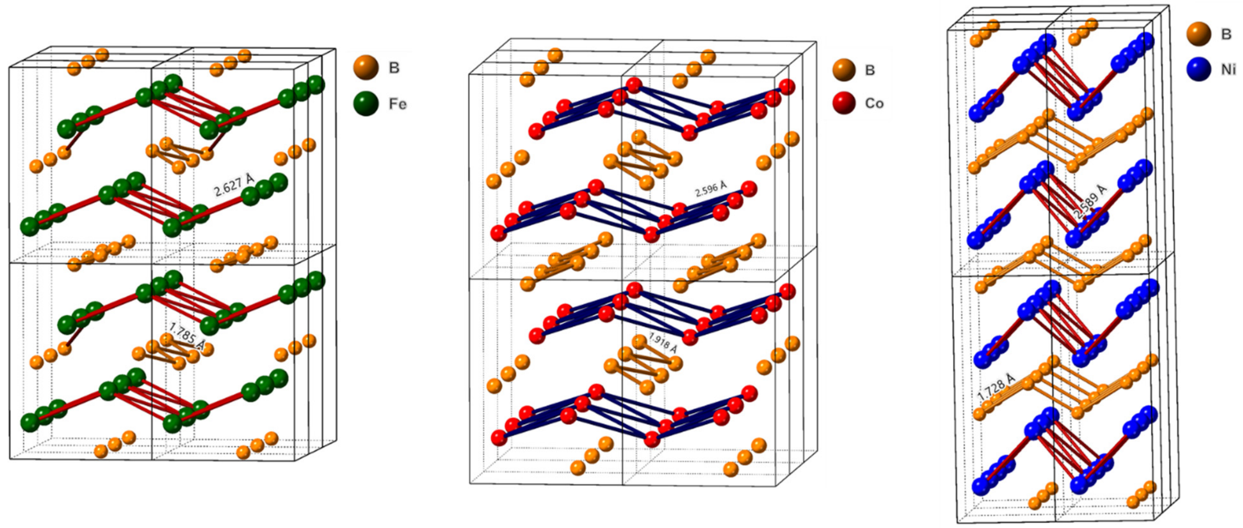

Figure S3B. Structural comparisons of FeB, CoB, and NiB showing M-M and B-B framework distances. In each structure, the B-B bonding is shown as zigzag chains either perpendicular or parallel to the page.

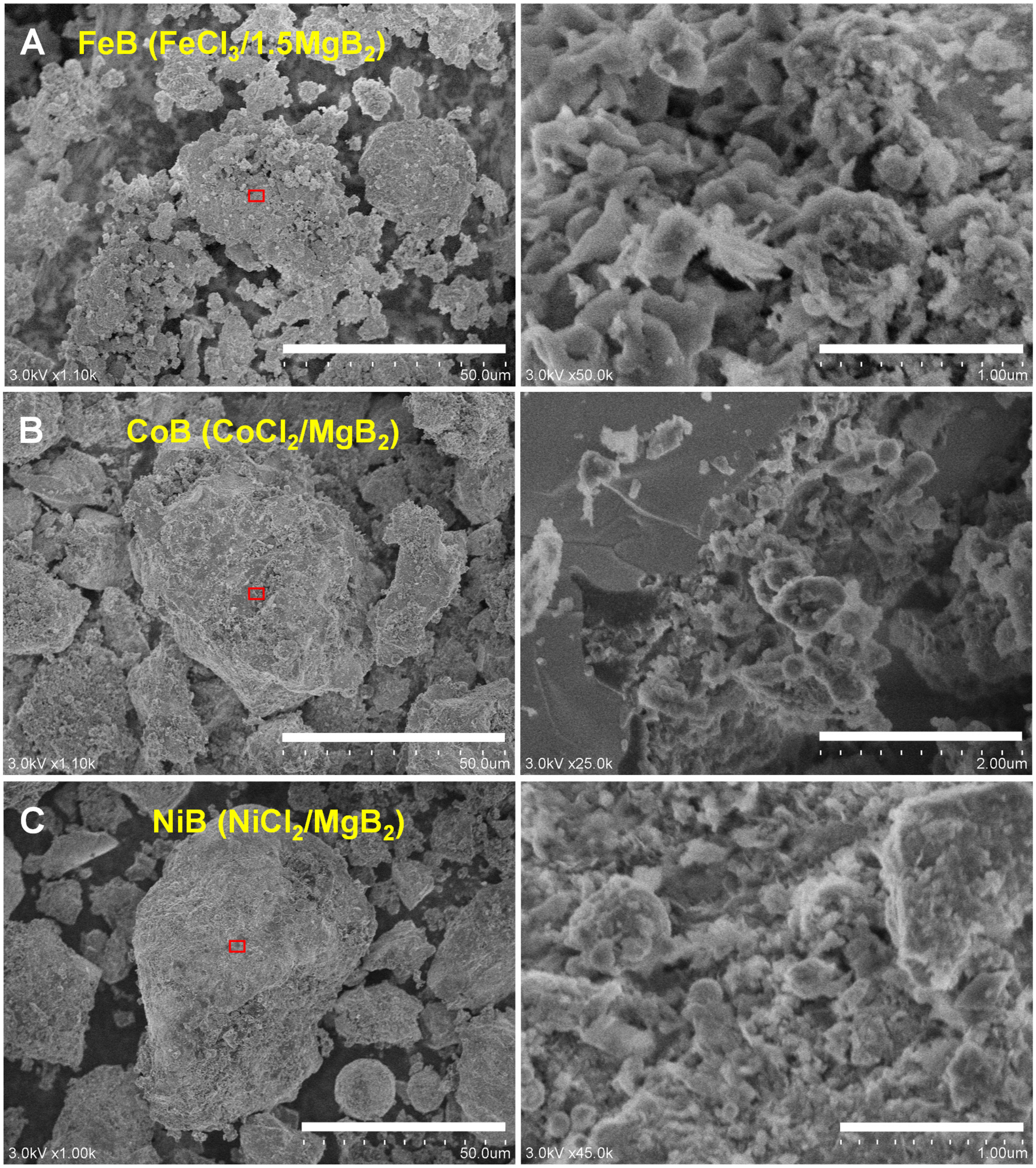

Figure S4. Scanning electron microscopy (SEM) comparison of particle morphologies obtained from SSM reactions of MCl_x with MgB_2 producing (A) FeB, (B) CoB, and (C) NiB (left column-low magnification images and right column-high magnification images). Zoomed areas highlighted by red boxes in the low magnification images on the left produced high magnification images on the right. The lengths of the scale bars are (left, right): (A) 50 μm and 1 μm, (B) 50 μm and 2 μm, (C) 50 μm and 1 μm.

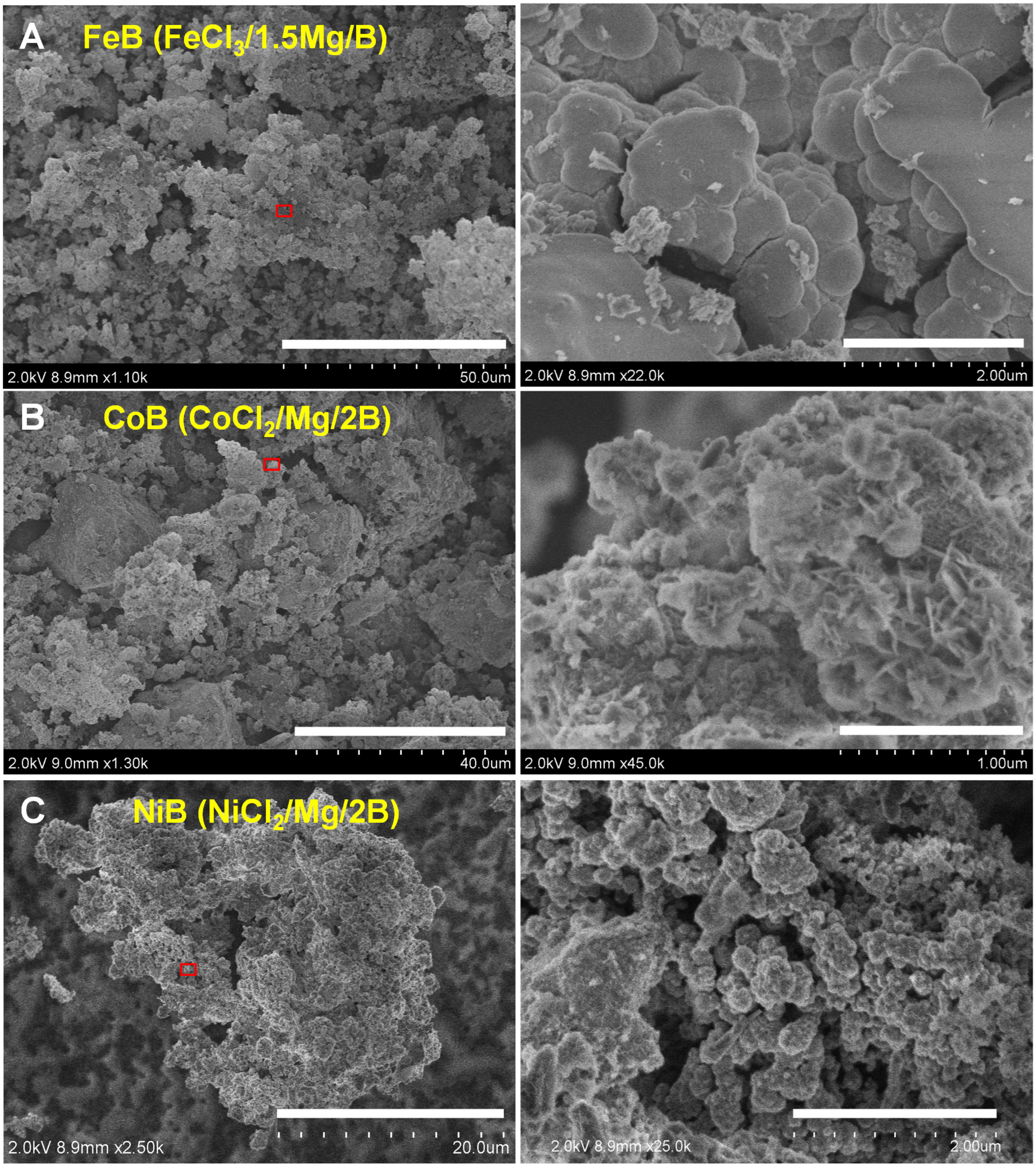

Figure S5. Scanning electron microscopy (SEM) comparison of particle morphologies obtained from MCl_x SSM reactions with Mg/xB producing (A) FeB, (B) CoB, and (C) NiB (left column-low magnification images and right column-high magnification images). Zoomed areas highlighted by red boxes in the low magnification images on the left produced high magnification images on the right. The lengths of the scale bars are (left, right): (A) 50 μm and 2 μm, (B) 40 μm and 1 μm, (C) 20 μm and 2 μm.

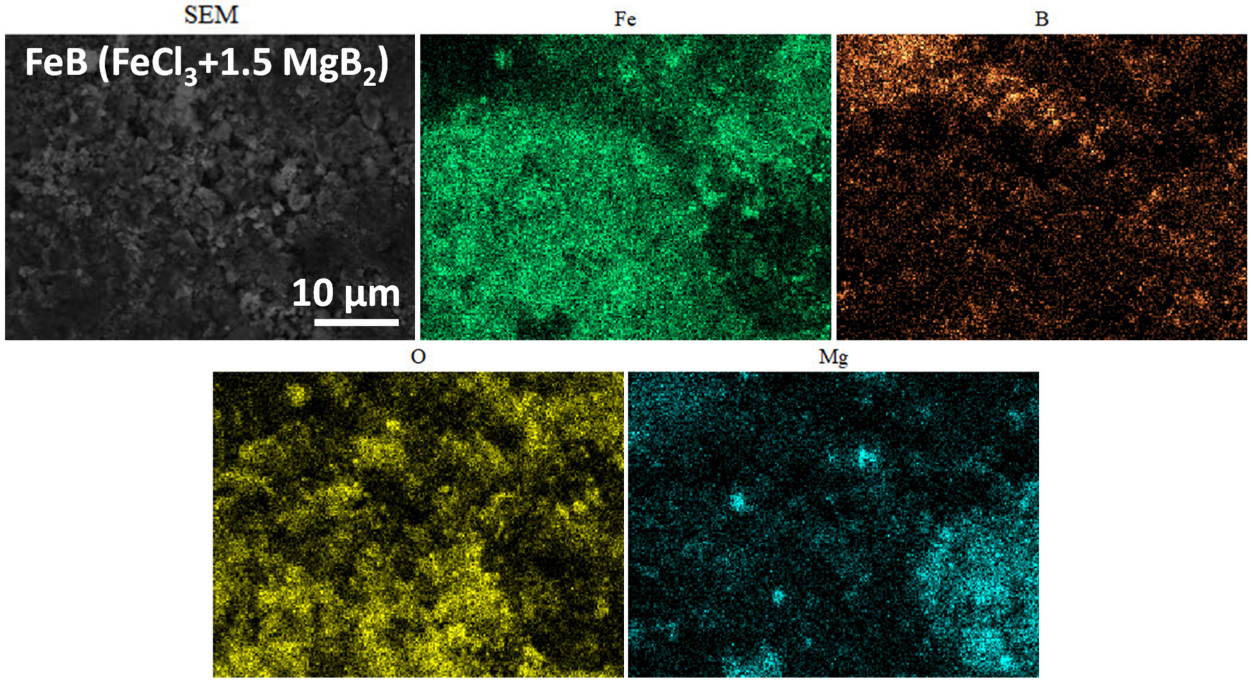

Figure S6. EDS maps of FeB formed from $FeCl_3$+1.5MgB_2 reaction before electrochemistry measurements. A thin (~1 mm height) pelletized sample was used for EDS analysis.

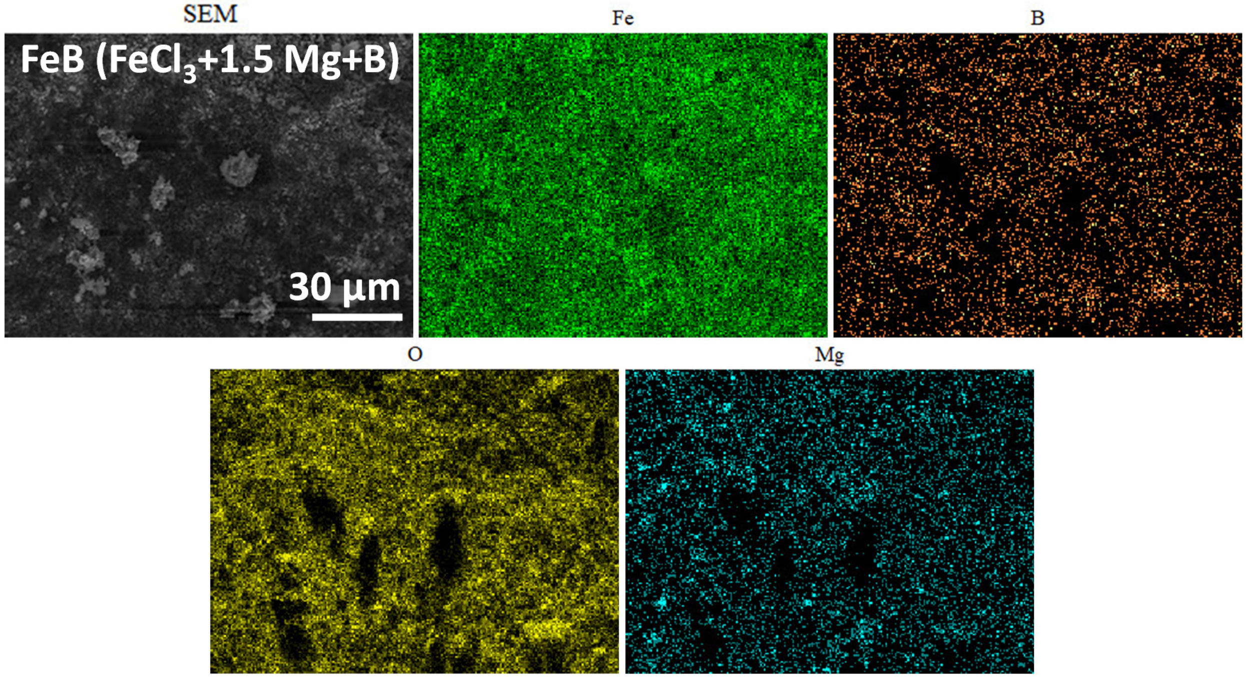

Figure S7. EDS maps of FeB formed from $FeCl_3$+1.5Mg+B reaction before electrochemistry measurements. A thin (~1 mm height) pelletized sample was used for EDS analysis.

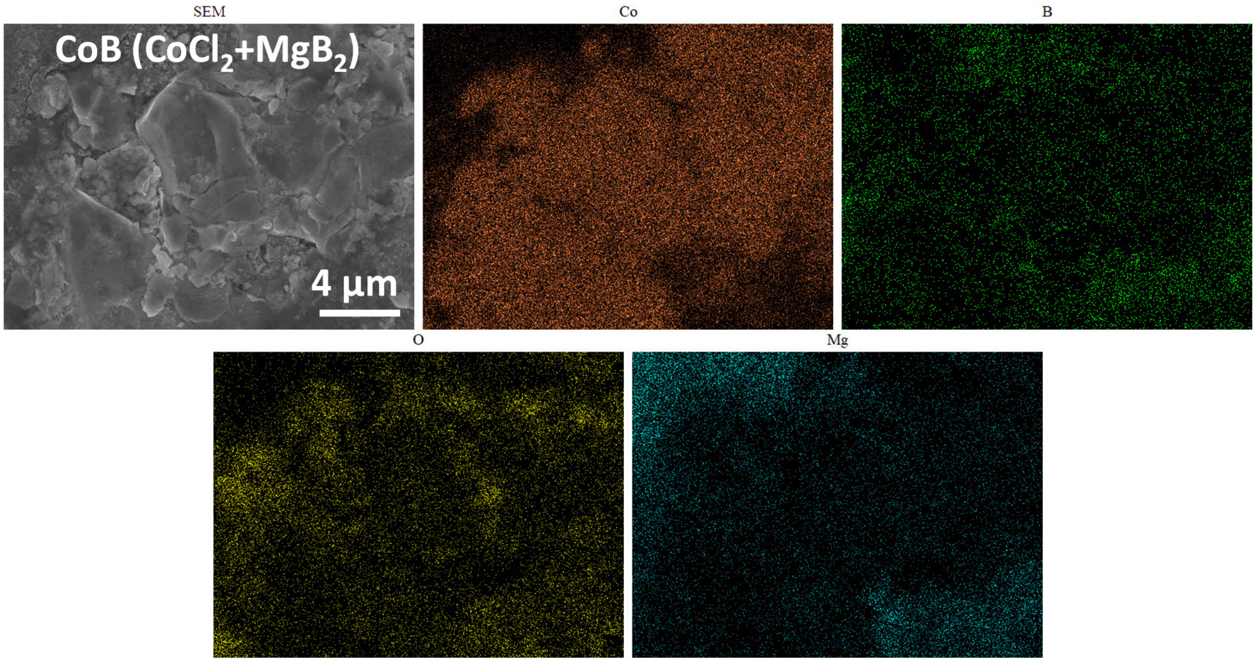

Figure S8. EDS maps of CoB formed from $CoCl_2$+MgB_2 reaction before electrochemistry measurements. A thin (~1 mm height) pelletized sample was used for EDS analysis.

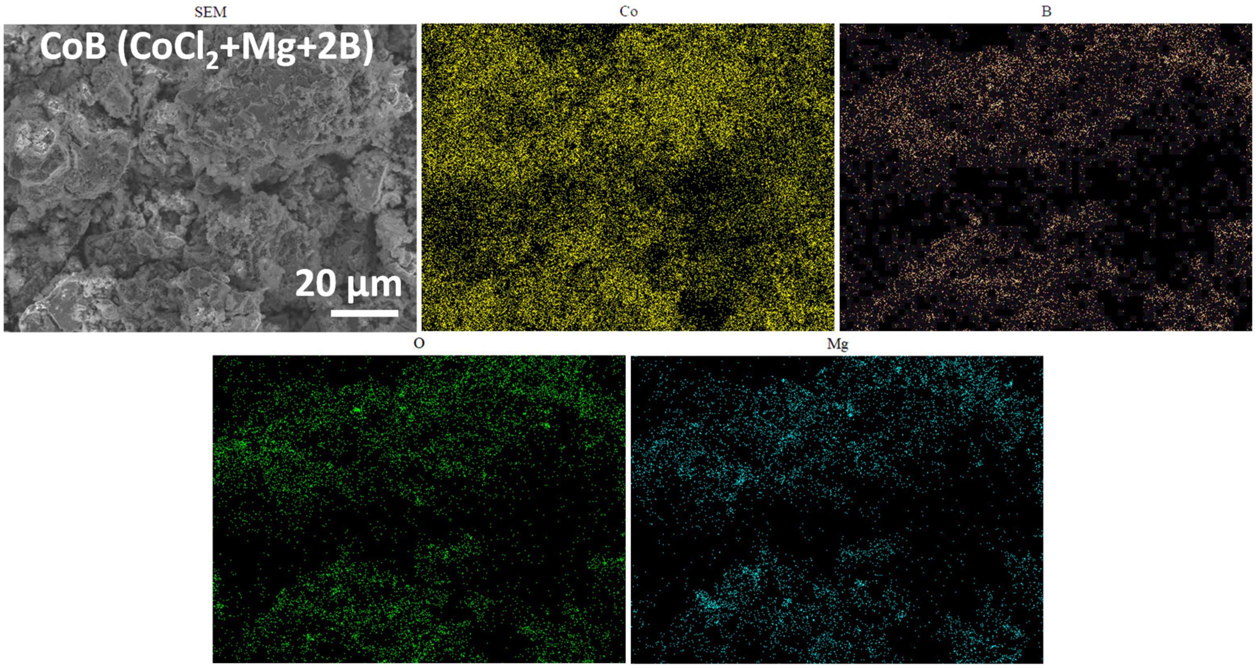

Figure S9. EDS maps of CoB formed from $CoCl_2$+Mg+2B reaction before electrochemistry measurements. A thin (~1 mm height) pelletized sample was used for EDS analysis.

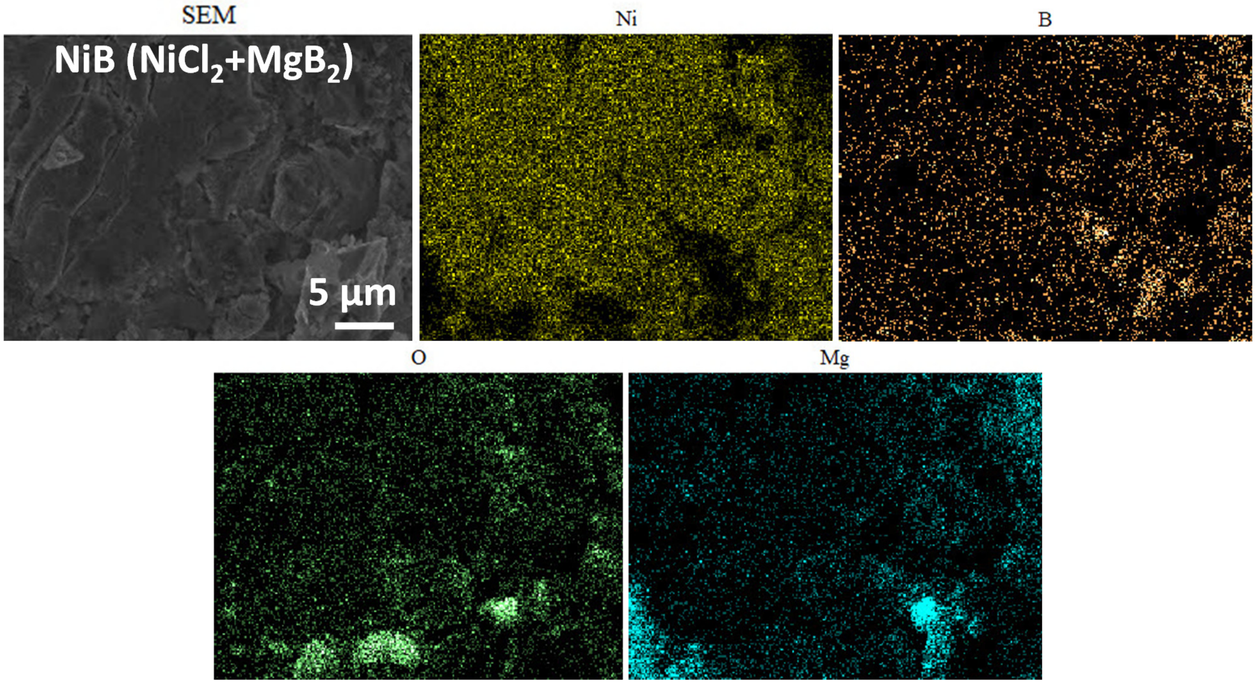

Figure S10. EDS maps of NiB formed from $NiCl_2$+MgB_2 reaction before electrochemistry measurements. A thin (~1 mm height) pelletized sample was used for EDS analysis.

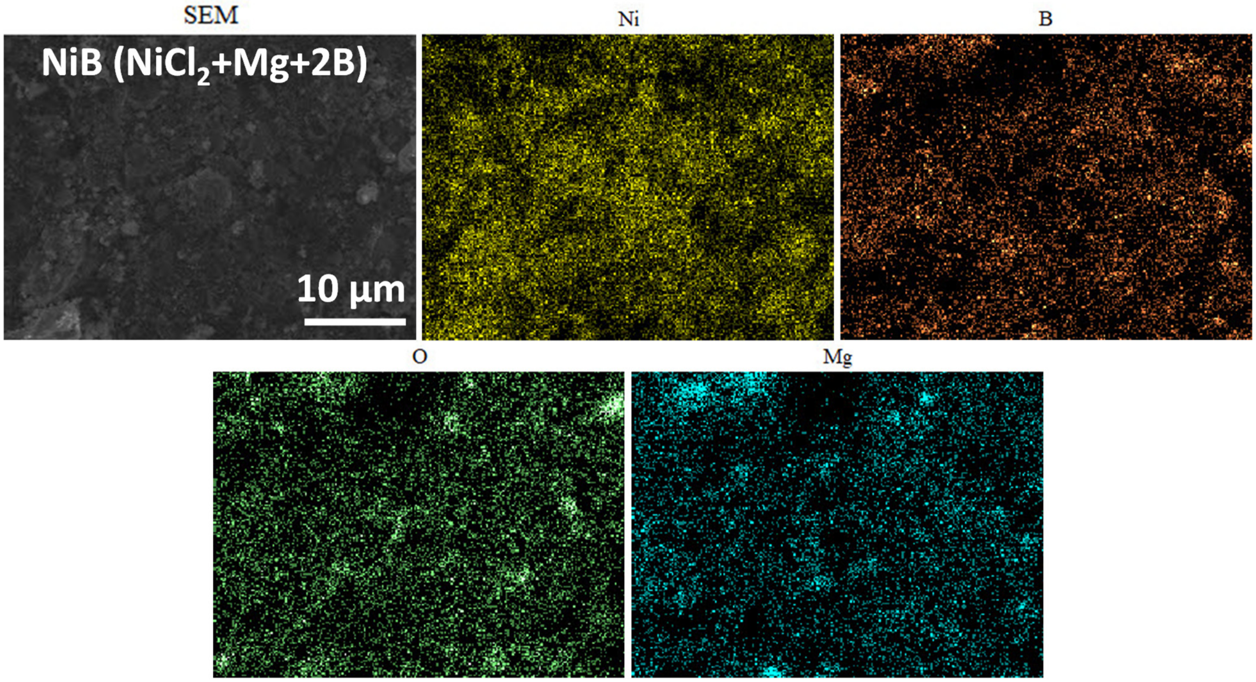

Figure S11. EDS maps of NiB formed from $NiCl_2$+Mg+2B reaction before electrochemistry measurements. A thin (~1 mm height) pelletized sample was used for EDS analysis.

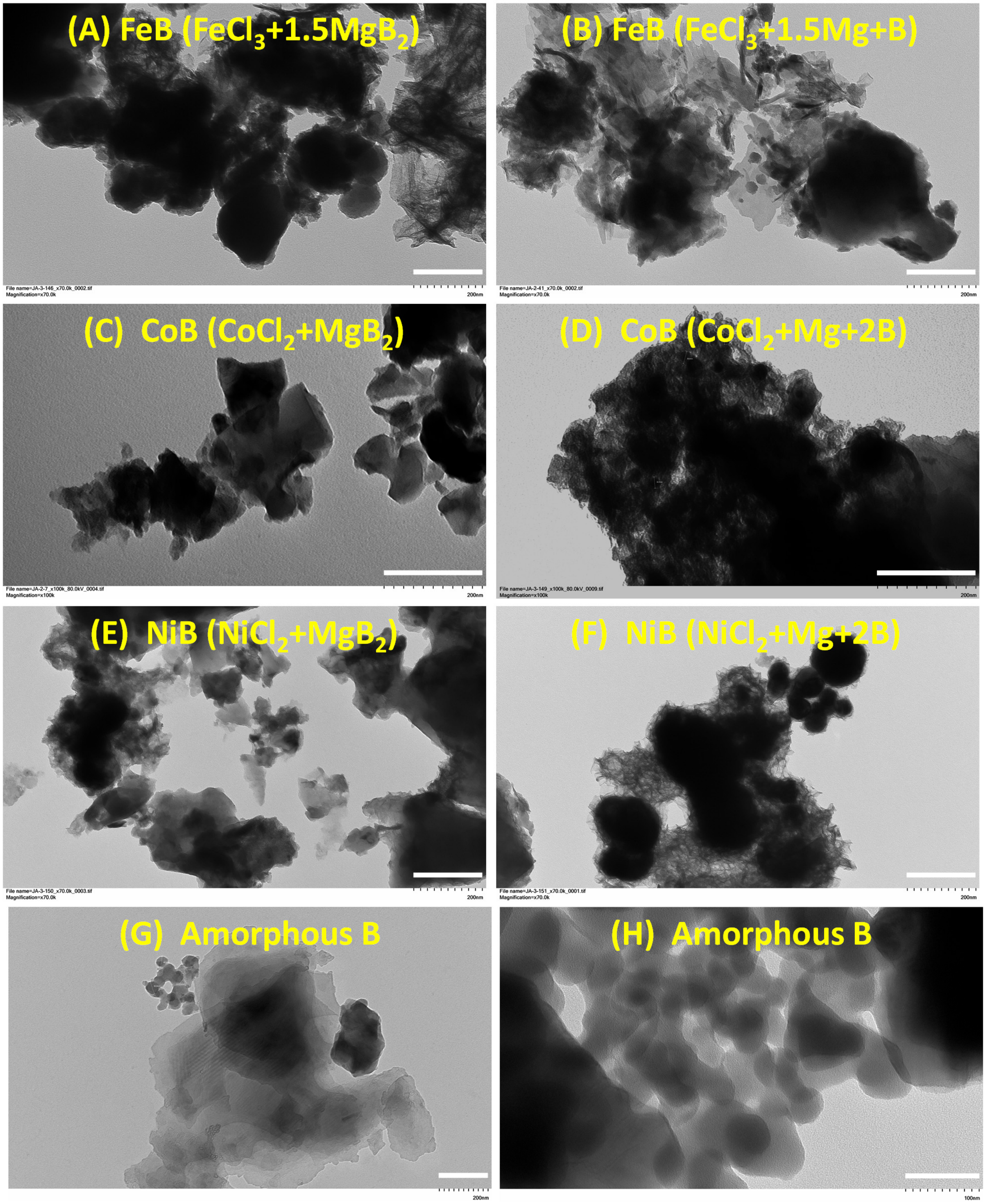

Figure S12. Additional TEM images of MBs and amorphous B. Scale bar lengths: (A), (B), (C), (D), (E), (F), and (G) are 200 nm, and (H) is 100 nm.

Table S5. Summary of XPS peak deconvolutions from **Figure S13** below.

Major peaks from deconvolution are listed below with literature reference values.[1-3] Energies are aligned in the row when possible. Metal shakeup satellite (ss) peaks noted.

MB	FeB (MgB_2)	FeB (Mg/B)	CoB (MgB_2)	NiB (MgB_2)	Literature (eV)
M $2p_{3/2}$ (eV)	711.6<br>716.6	714.4<br>715.8			FeB 707.4<br>Fe_2O_3 711.6
			782.5<br>786.5 (ss)		CoB 778.3<br>$Co(OH)_2$ 782.1
				853.0<br>857.2<br>862.7 (ss)	NiB 853.4<br>$Ni(OH)_2$ 856
B 1s (eV)			187.2<br>189.2	188.4<br>190.1	B(amorph) 187.3<br>FeB 187.9
	192.5<br>195.6	192.1<br>195.3<br>196.3	192.1<br>193.6	192.2<br>194.3	CoB 187.3<br>NiB 188.9<br>$FeBO_3$ 192
	197.2 (Cl?)			198.3<br>200.3	B_2O_3 193.7<br>$B(OH)_3$ 193.4<br>KCl (Cl 2p3/2) 197.8
O 1s (eV)					Fe_2O_3 530.1
		529.5			Co_3O_4 529.6
	531.8<br>535.9<br>537.4 (unk)	532.8<br>534.9	532.3<br>533.6	532.2<br>533.8	NiO 529.2<br>B_2O_3 533.2<br>$Na_2B_4O_7$ 533.7

FeB from MgB_2

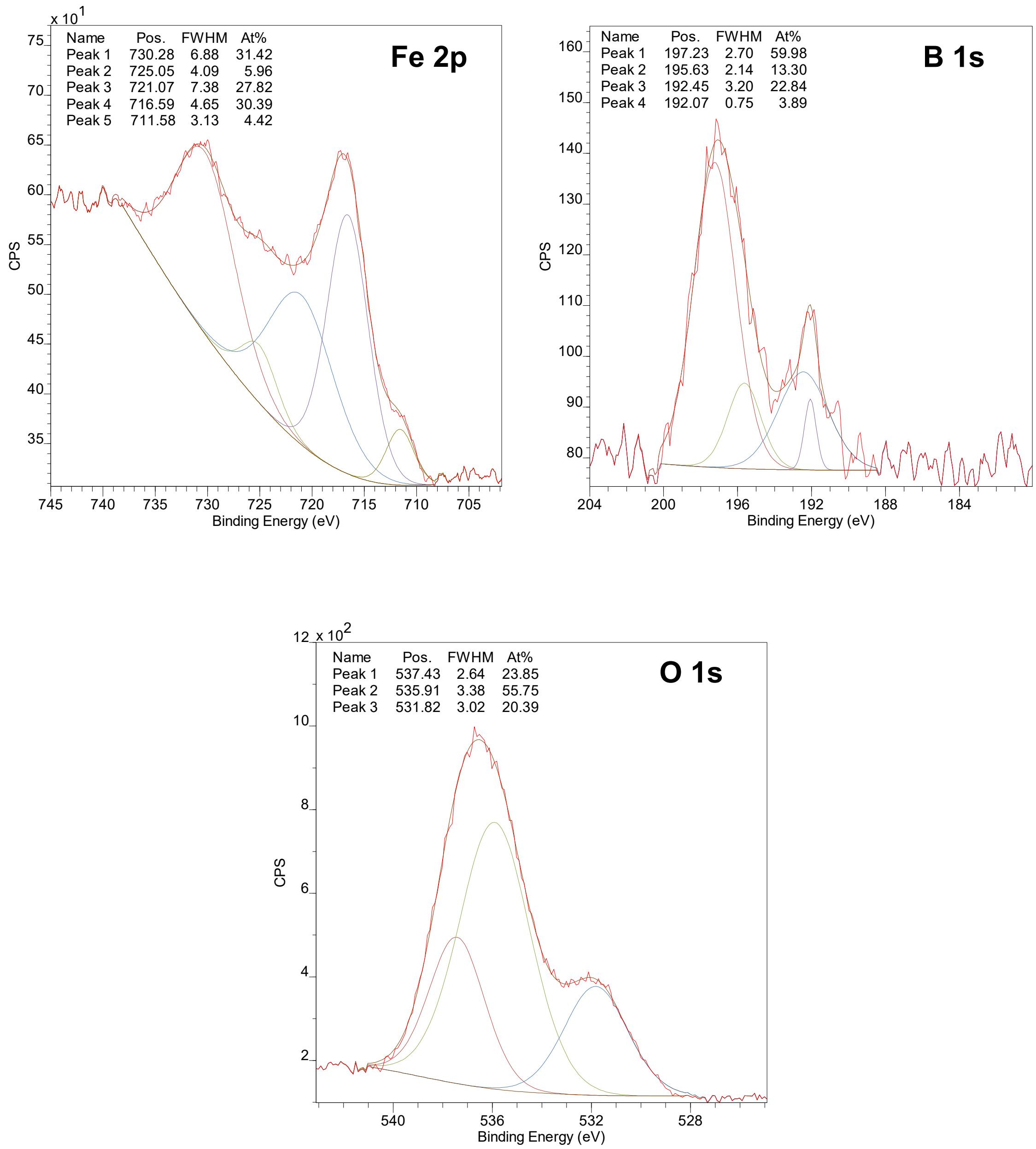

Figure S13A. X-ray photoelectron spectroscopy (XPS) regional scans for FeB powder produced from $FeCl_3$+1.5 MgB_2 SSM reactions. Ideal product is FeB+2B.

FeB from Mg/B

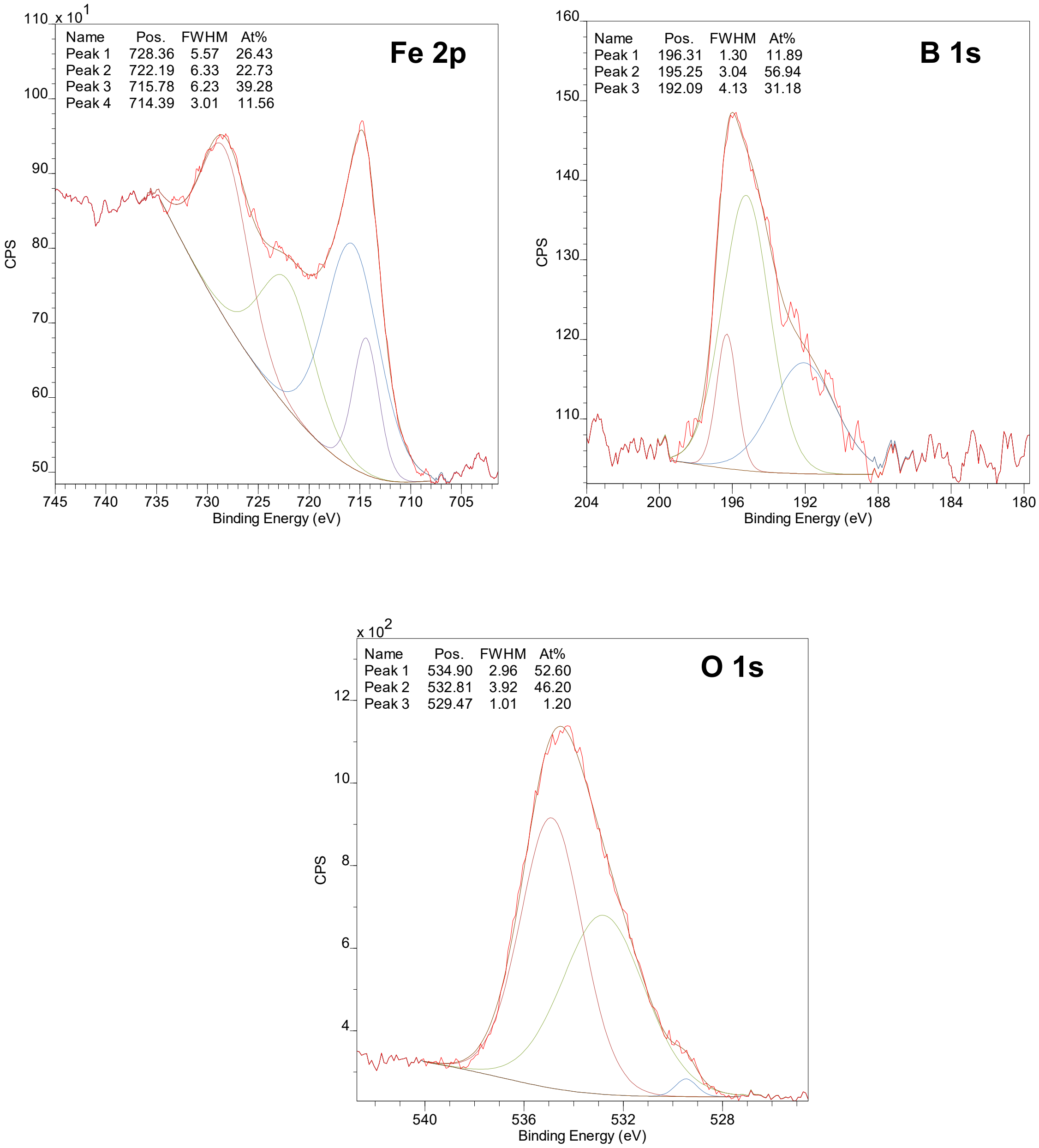

Figure S13B. X-ray photoelectron spectroscopy (XPS) regional scans for FeB powder produced from $FeCl_3$+1.5 Mg+B SSM reactions. Ideal product is FeB.

CoB from MgB_2

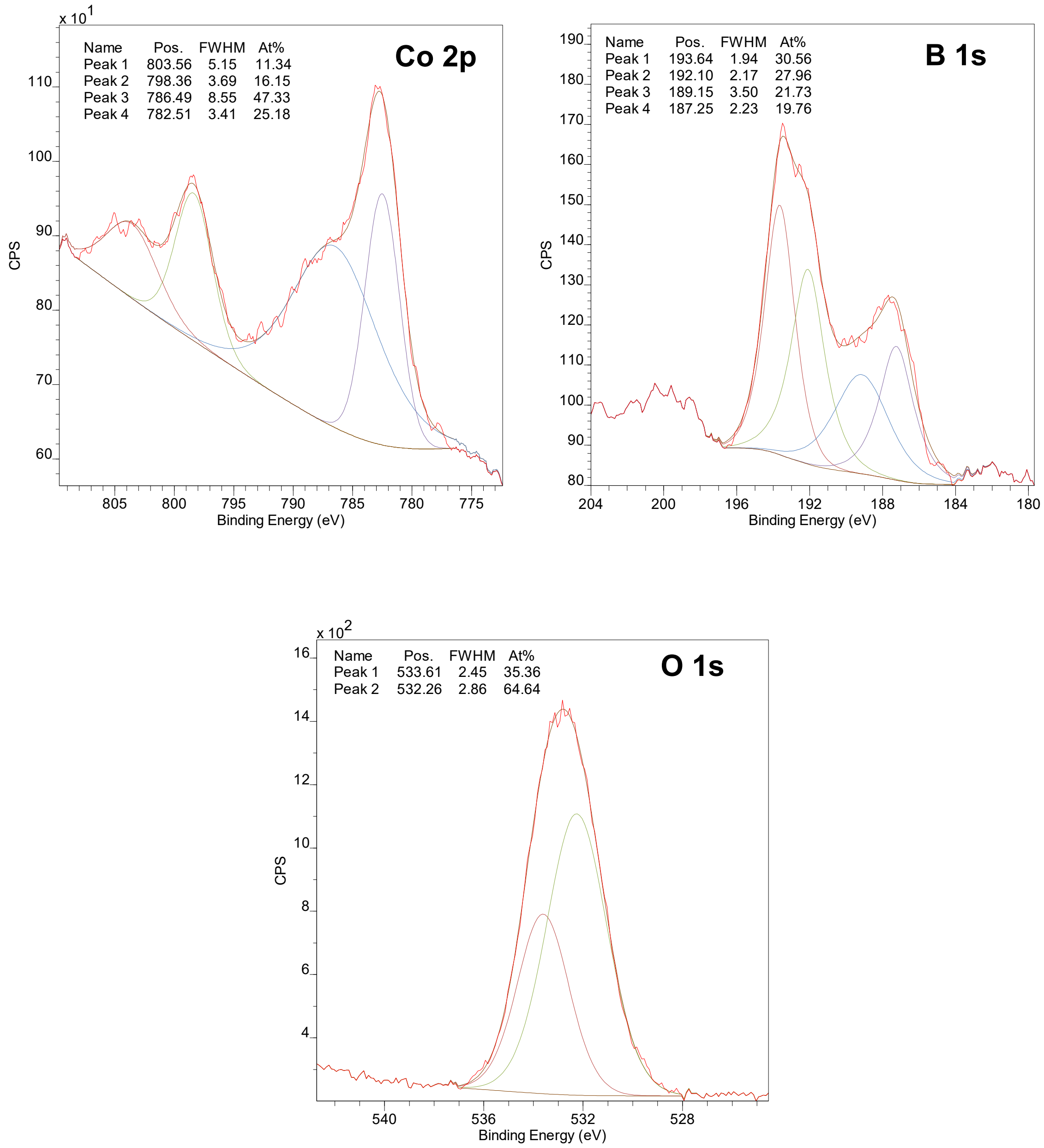

Figure S13C. X-ray photoelectron spectroscopy (XPS) regional scans for CoB powder produced from $CoCl_2$+MgB_2 SSM reactions. Ideal product is CoB+B.

NiB from MgB_2

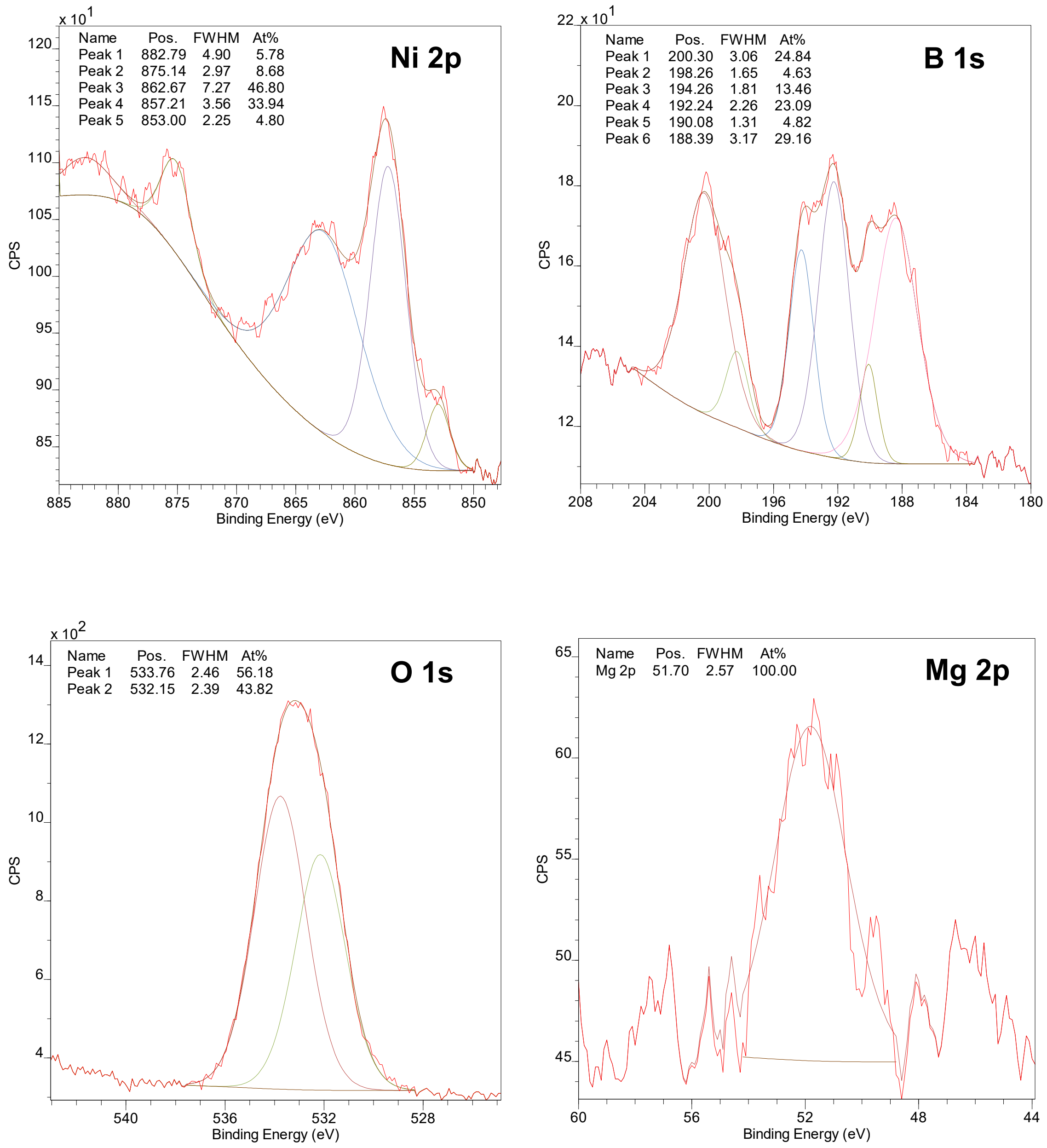

Figure S13D. X-ray photoelectron spectroscopy (XPS) regional scans for NiB powder produced from $NiCl_2$+MgB_2 SSM reactions. Ideal product is NiB+B. Mg^{2+} (Mg 2p 52 eV) and Cl^- (Cl 2p ~200 eV in B 1s regional scan) are observed on the surface of this sample, likely from $MgCl_2$ residues.

Sample calculations for reaction heat and adiabatic temperature
All thermochemical data of compounds were obtained from several thermochemical references sources.[4-7]

SSM reactions enthalpy calculations. An example of a thermochemical ΔH_{rxn} calculation that was done using Hess's Law for the $FeCl_3/MgB_2$ reaction.

	$FeCl_3$	+	1.5 MgB_2	→	FeB	+	1.5 $MgCl_2$	+	4 B
ΔH_f(kJ/mol)	-399.2		-60.7×1.5		-72.8		-644.2×1.5		0

ΔH_{rxn} (kJ/mol FeB) $= \Sigma\Delta H_f(\text{products}) - \Sigma\Delta H_f(\text{reactants}) = -548.9$

Table S6. Thermochemical data for MCl_2/MgB_2 and $MCl_2/Mg/2B$ reactions forming FeB, CoB, and NiB.

Precursor (MCl_x)	ΔH_f of MCl_x (kJ/mol)	Product (MB)	ΔH_f of MB (kJ/mol)	ΔH_{rxn} (kJ/mol MB) of MCl_x/MgB_2 reaction	ΔH_{rxn} (kJ/mol MB) of $MCl_x/Mg/2B$ reaction
$FeCl_3$	-399.2	FeB	-72.8	-548.9*	-639.9▫
$FeCl_2$	-341.6	FeB	-72.8	-314.7	-375.4
$CoCl_2$	-312.5	CoB	-94.1	-365.1	-425.8
$NiCl_2$	-305.3	NiB	-46.4	-324.6	-385.3

*ΔH_{rxn} (kJ/mol FeB) value of $FeCl_3$/1.5 MgB_2 reaction ▫ΔH_{rxn} (kJ/mol FeB) value of $FeCl_3$/1.5 Mg/B reaction.

Maximum adiabatic reaction temperature (T_{ad}) calculations. For the T_{ad} calculations, complete reaction progress and no heat loss of reactions were assumed. Even though SSM reactions are very rapid, some heat loss from the reactor to the surrounding environment is possible in practice. As a result, actual maximum reaction temperatures may be lower than calculated adiabatic reaction temperatures (T_{ad}). The standard molar heat capacities (298 K and 1 atm) were used for solid compounds and molar heat capacities at melting and boiling temperatures were selected as the heat capacity of compounds in liquid and gas phases at 298 K.[4] Molar heat capacities of materials (mostly solids) generally increase with temperature, so the T_{ad} values represent upper limits of likely temperatures reached in these SSM reactions.

The T_{ad} calculation of $FeCl_3$/1.5MgB_2 reaction.

T_{ad} calculation for the FeB formation from $FeCl_3/MgB_2$ reaction

	$FeCl_3$	+	1.5 MgB_2	→	FeB	+	1.5 $MgCl_2$	+	2B	ΔH°_{rxn}	-548.9 kJ/mol
ΔH°_f (kJ/mol)	-399.2		-60.7		-72.8		-644.2		0		
mp (°C)	304		800 dec		1590		707		2077		
bp (°C)	331						1412		3866		
Heat of fusion (kJ/mol)							40.0				
Heat of vaporization (kJ/mol)							209.1				
C_p (J/mol K) (solid)					41.0		71.3		10.71		
C_p (J/mol K) (liquid)							92.8		31.75		
C_p (J/mol K) (gas)							57.1		20.8		

		$\Delta H = C_p\Delta T$
25 °C -707 °C	Products heat up to $MgCl_2$ melting temp.	115
707 °C	$MgCl_2(s) \rightarrow MgCl_2(l)$	60
707 °C - 1412 °C	Products heat up to $MgCl_2$ boiling temp.	142
1412 °C	$MgCl_2(l) \rightarrow MgCl_2(g)$	231

Final T_{ad} = 1412 °C

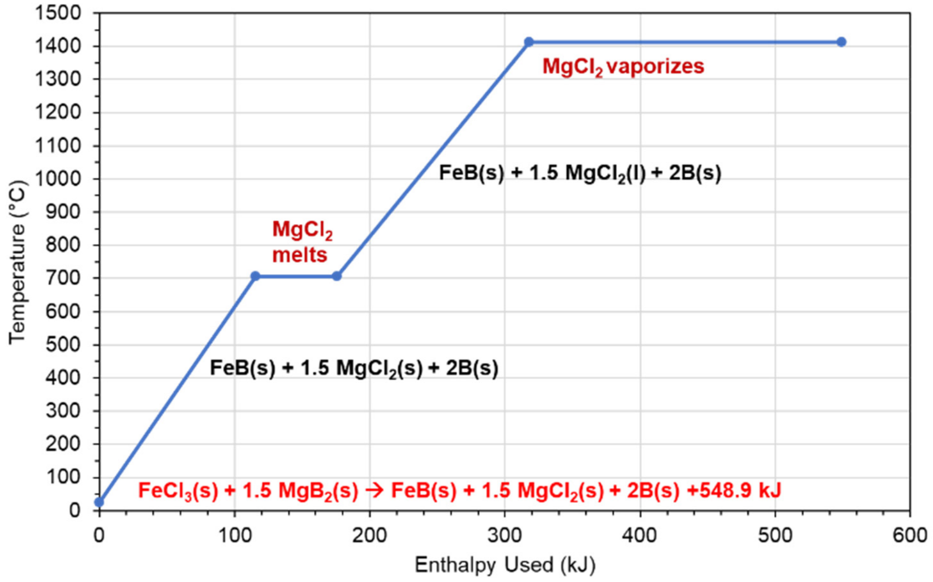

Figure S14. Graph showing theoretical enthalpy versus temperature changes based on an adiabatic system for FeB formation from an SSM reaction. The theoretical maximum temperature is represented by the point where all reaction enthalpy is expended.

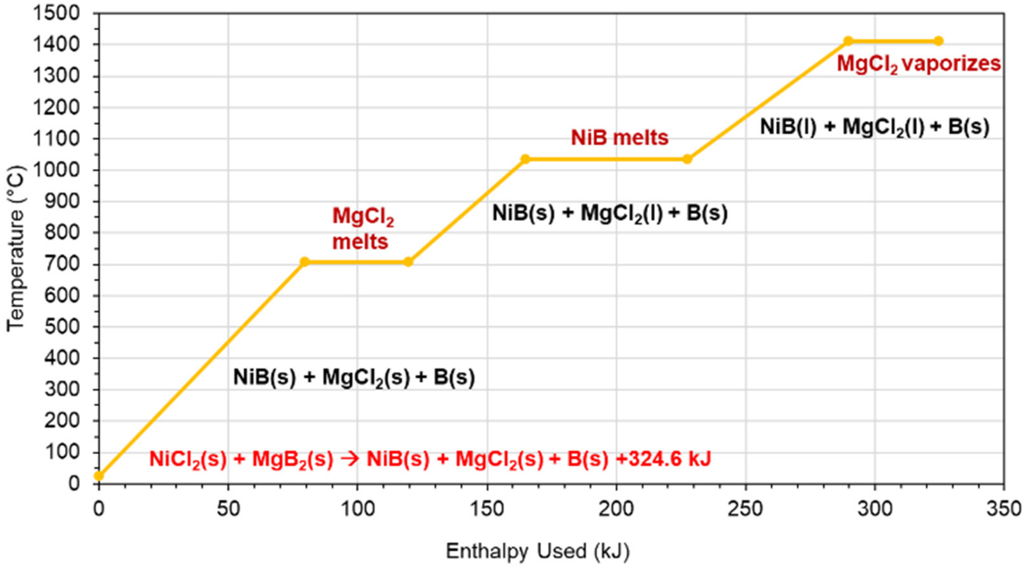

Figure S15. Graph showing theoretical enthalpy versus temperature changes based on an adiabatic system for NiB formation from an SSM reaction. The theoretical maximum temperature is represented by the point where all reaction enthalpy is expended.

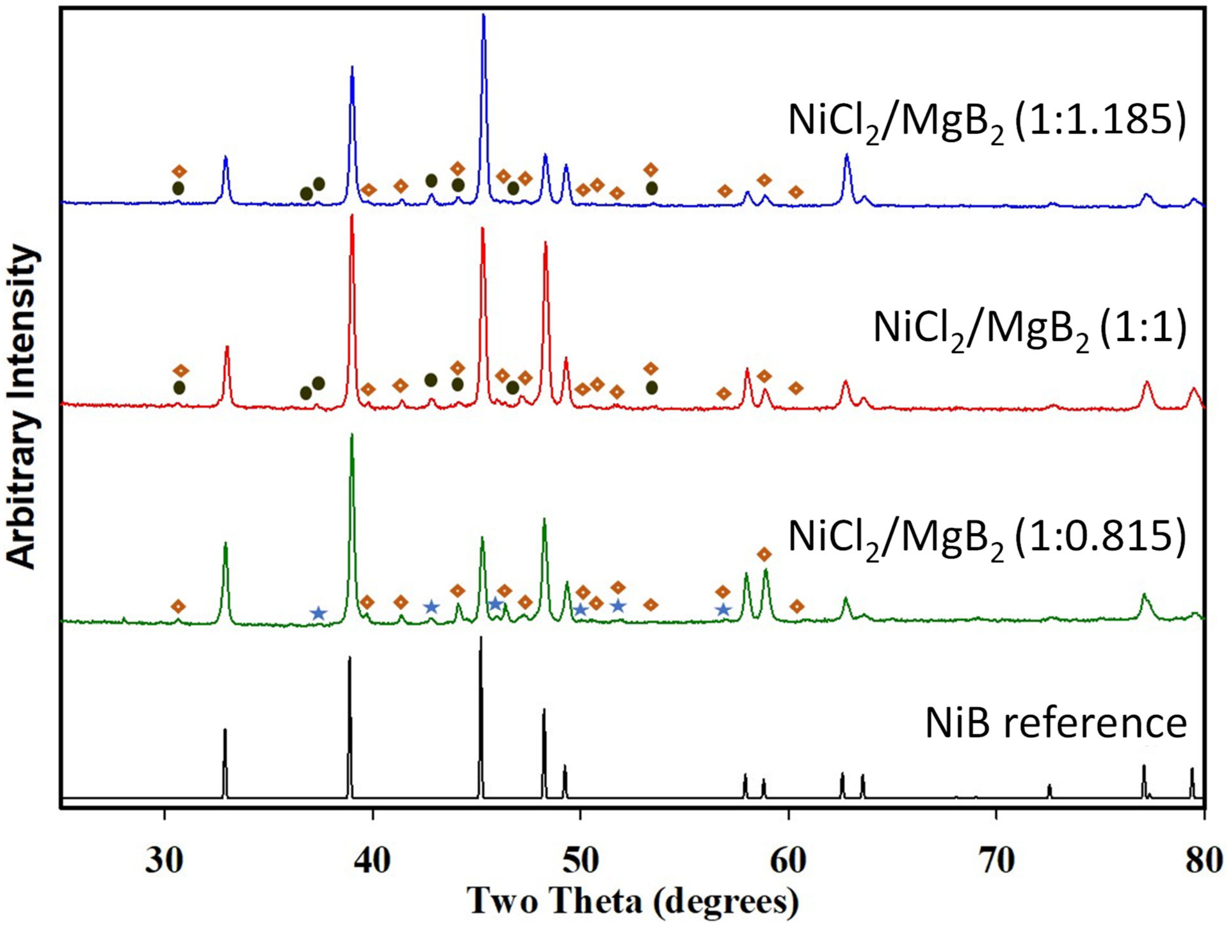

Figure S16. XRD results for $NiCl_2/MgB_2$ reactions run above and below salt balanced stoichiometries. SSM reactions were run with $NiCl_2:MgB_2$ ratios as 1:1.185, 1:1, and 1:0.815 from top to bottom, respectively. Secondary labeled phases are *m*-Ni_4B_3 (◊), $MgNi_3B_2$ (•), Ni_2B (blue stars). Some peaks (mostly $2\theta > 50$) of Ni_4B_3, $MgNi_3B_2$, and Ni_2B cannot be assigned precisely due to the low peak intensity.

Table S7. Salt dilution results of $NiCl_2$+MgB_2 and $NiCl_2$+Mg+2B reactions.

Reaction	Products	Yield %	SA (m^2/g)	ΔH_{rxn} (kJ/mol)	T_{ad} (°C)
$NiCl_2$+MgB_2→NiB+$MgCl_2$+B	**NiB**, Ni_4B_3, $MgNi_3B_2$	80.1	2	-324.6	1412
$NiCl_2$+MgB_2+0.5$MgCl_2$→NiB+1.5$MgCl_2$+B	**NiB**, Ni_2B, Ni_4B_3, Ni	77.1	3	-324.6	1213
$NiCl_2$+MgB_2+$MgCl_2$→NiB+2$MgCl_2$+B	**Ni_2B**, NiB, Ni_4B_3	53.0	5	-324.6	1035
$NiCl_2$+MgB_2+2$MgCl_2$→NiB+3$MgCl_2$+B	**Ni_2B**, Ni, Ni_3B, MgB_2	35.7	-	-324.6	793
$NiCl_2$+Mg+2B+→NiB+$MgCl_2$+B	**NiB**	86.3	3	-385.3	1412
$NiCl_2$+Mg+2B+0.5$MgCl_2$→NiB+1.5$MgCl_2$+B	**NiB**, Ni_2B, Ni_4B_3	89.3	3	-385.3	1412
$NiCl_2$+Mg+2B+$MgCl_2$→NiB+2$MgCl_2$+B	**NiB**, Ni_2B, Ni_4B_3	77.6	6	-385.3	1185
$NiCl_2$+Mg+2B+2$MgCl_2$→NiB+3$MgCl_2$+B	**Ni_2B**, NiB, Ni_4B_3, Ni	36.9	-	-385.3	980

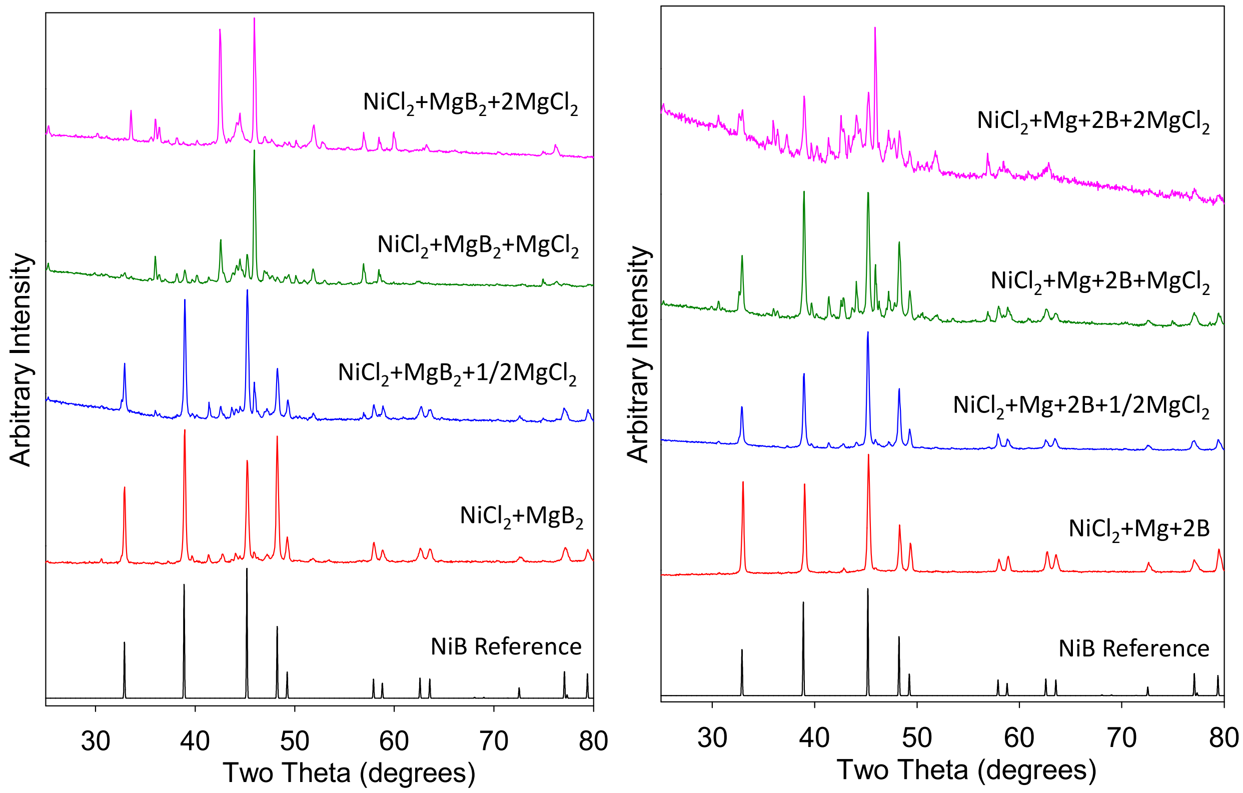

Figure S17. XRD results of $NiCl_2$/MgB_2/$MgCl_2$ and $NiCl_2$/Mg/2B/$MgCl_2$ salt dilution reactions.

Table S8. Salt dilution results of $CoCl_2$+MgB_2 and $CoCl_2$+Mg+2B reactions.

Reaction	Products	Yield %	SA (m^2/g)	ΔH_{rxn} (kJ/mol)	T_{ad} (°C)
$CoCl_2+MgB_2 \rightarrow CoB+MgCl_2+B$	**CoB**	80.1	3	-365.1	1412
$CoCl_2+MgB_2+0.5MgCl_2 \rightarrow CoB+1.5MgCl_2+B$	**CoB**	69.2	7	-365.1	1412
$CoCl_2+MgB_2+MgCl_2 \rightarrow CoB+2MgCl_2+B$	**CoB**, Co_2B, Co	79.5	13	-365.1	1386
$CoCl_2+MgB_2+2MgCl_2 \rightarrow CoB+3MgCl_2+B$	**MgB_2**, Co_2B, CoB, Co	27.0	-	-365.1	918
$CoCl_2+Mg+2B \rightarrow CoB+MgCl_2+B$	**CoB**	84.7	2	-425.8	1412
$CoCl_2+Mg+2B+0.5MgCl_2 \rightarrow CoB+1.5MgCl_2+B$	**CoB**	88.3	9	-425.8	1412
$CoCl_2+Mg+2B+MgCl_2 \rightarrow CoB+2MgCl_2+B$	**CoB**	88.8	7	-425.8	1412
$CoCl_2+Mg+2B+2MgCl_2 \rightarrow CoB+3MgCl_2+B$	**CoB**	49.3	18	-425.8	1105

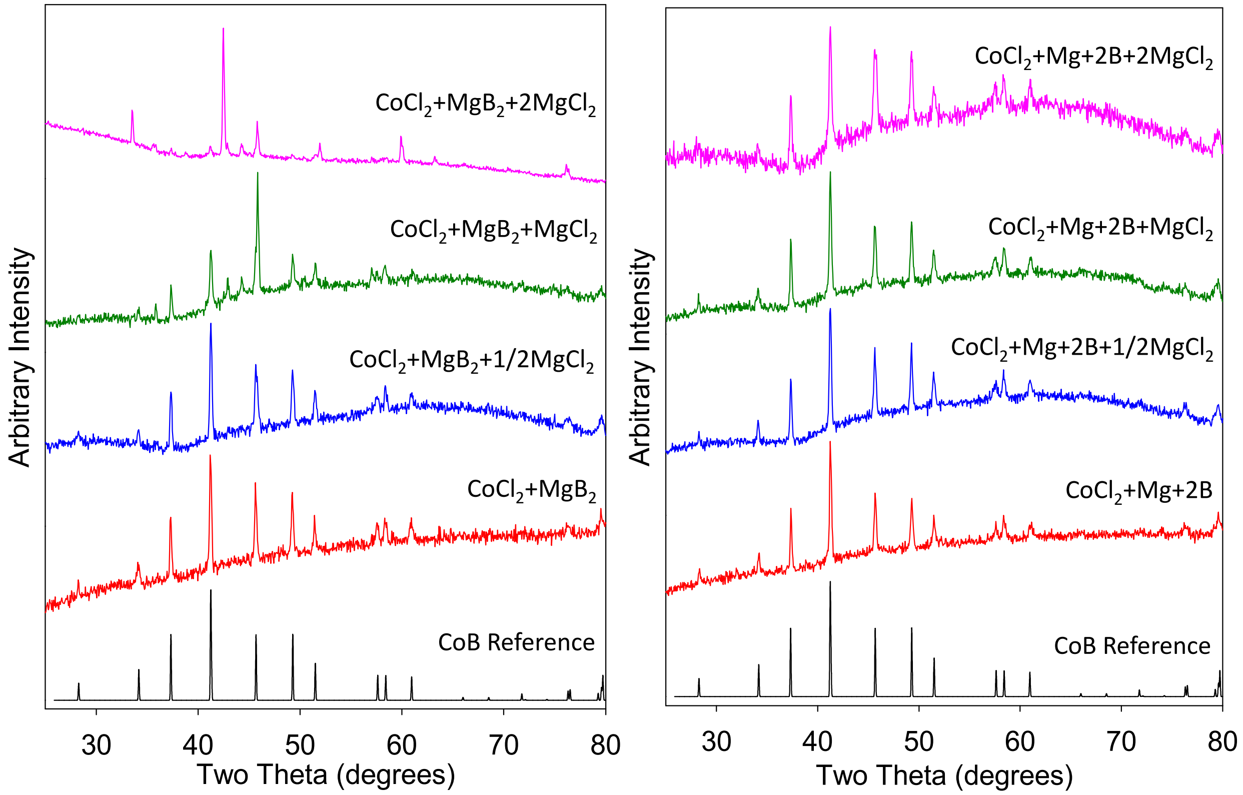

Figure S18. XRD results of $CoCl_2/MgB_2/MgCl_2$ and $CoCl_2$/Mg/2B/$MgCl_2$ salt dilution reactions.

Table S9. The sizes (in nm) of NiB and CoB crystallites as determined by X-ray line broadening analysis.

x	$CoCl_2$+MgB_2+x$MgCl_2$	$CoCl_2$+Mg+2B+x$MgCl_2$	$NiCl_2$+MgB_2+x$MgCl_2$	$NiCl_2$+Mg+2B+x$MgCl_2$
**0**	39	41	38	38
**0.5**	40	41	36	36
**1**	35	41	38	33
**2**	34	35	-	-

Table S10. Results for MCl_2/Mg rapid SSM reactions.

Reaction	% Yield based on target metal M	XRD structure	Magnetic attraction
**$CoCl_2$ / Mg**	62	Co	strong
**$FeCl_2$ / Mg**	56	Fe	strong
**$NiCl_2$ / 0.92 Mg**	55	Ni	strong

The Co and Fe were formed directly from MCl_2/Mg reaction using a stoichiometric molar amount of MCl_2 and Mg. However, the equivalent molar reaction of $NiCl_2$ and Mg resulted in Ni and trace amounts of a MgNi alloy (**Figure S19**). Crystalline Ni metal was obtained by using less than the stoichiometric amount of Mg required for a complete reaction. All three metals are ferromagnetic at room temperature, and they qualitatively show strong attraction to a permanent magnet.

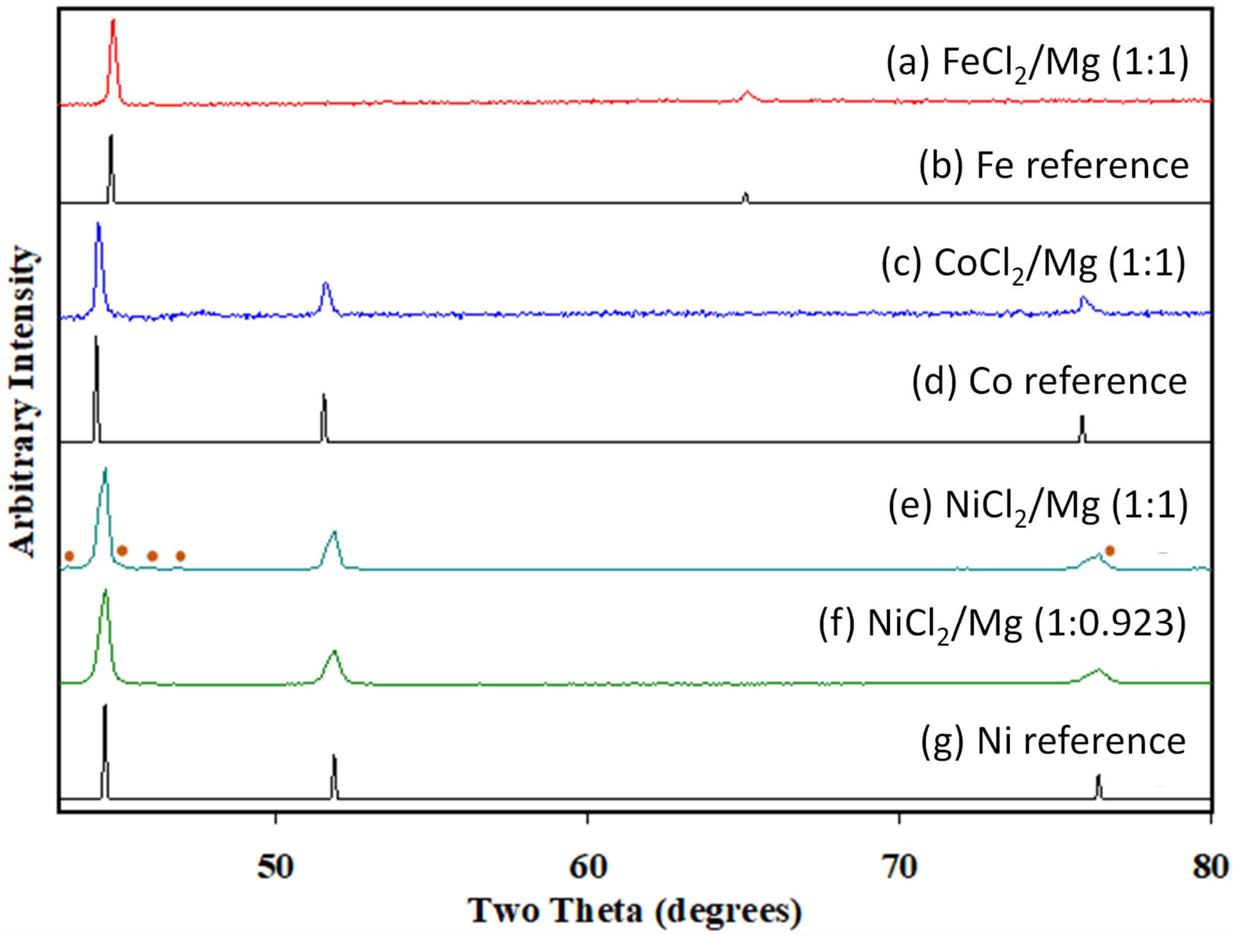

Figure S19. Powder XRD results of the MCl_2/Mg reaction targeting (a) Fe (c) Co, and (e, f) Ni. (e) is XRD obtained from 1:1 molar reaction of $NiCl_2$/Mg and (f) is XRD of 1:0.923 (6.5 mmol: 6.0 mmol) $NiCl_2$/Mg. (b), (d), and (g) are reference patterns of Fe, Co, and Ni elements, respectively. The red dots are MgNi alloy peaks.

Table S11. Results for metal and boron externally heated 500 °C ampoule reactions.

Reaction	Target	% yield based on target	BET	Magnetic attraction	XRD (major phase in bold)
Fe/B	FeB	92	2	strong	unidentified peaks
Co/B	CoB	94	4	strong	Co_2B, CoB
Ni/B	NiB	95	3	strong	**Ni**, Ni_3B, Ni_2B

M/B ampoule reactions for FeB, CoB, and NiB formation did not produce the desired single-phase MB products. These reaction products show multiple metal and metal boride phases and show strong attraction to a permanent magnet.

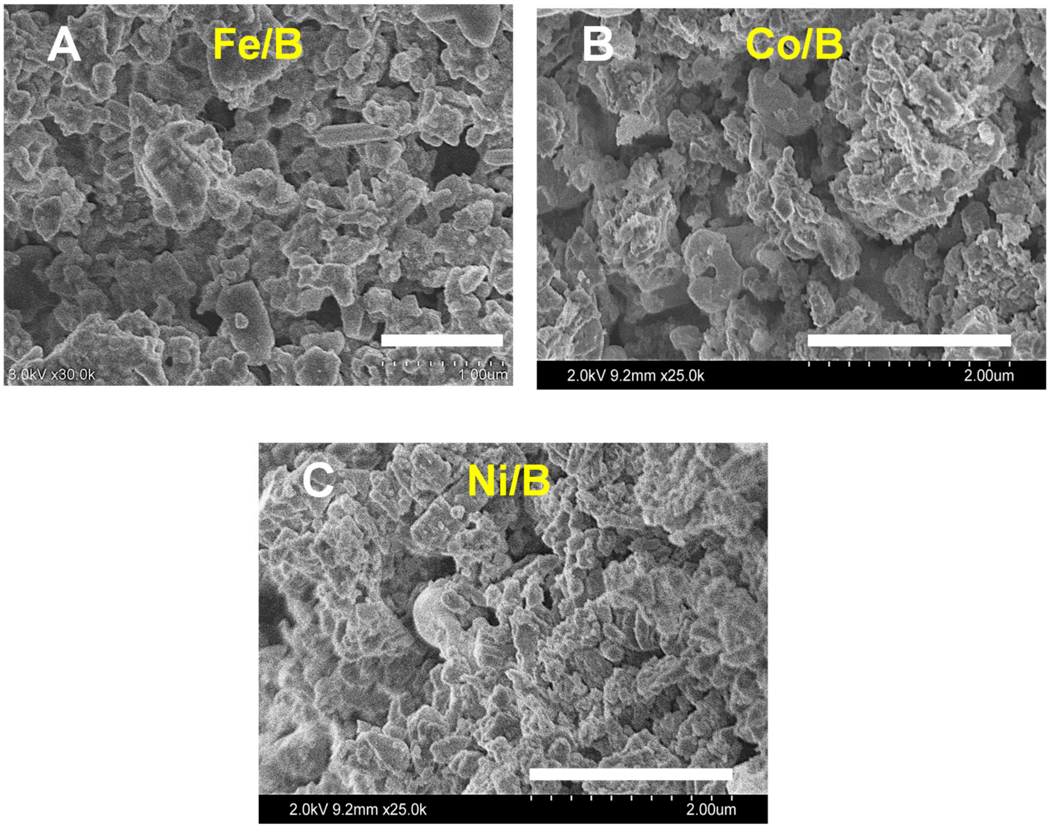

Figure S20. Comparison of particle morphologies obtained from M/B ampoule reactions at 500 °C for several days. The lengths of the scale bars are 1 μm for (A) and 2 μm for (B) and (C).

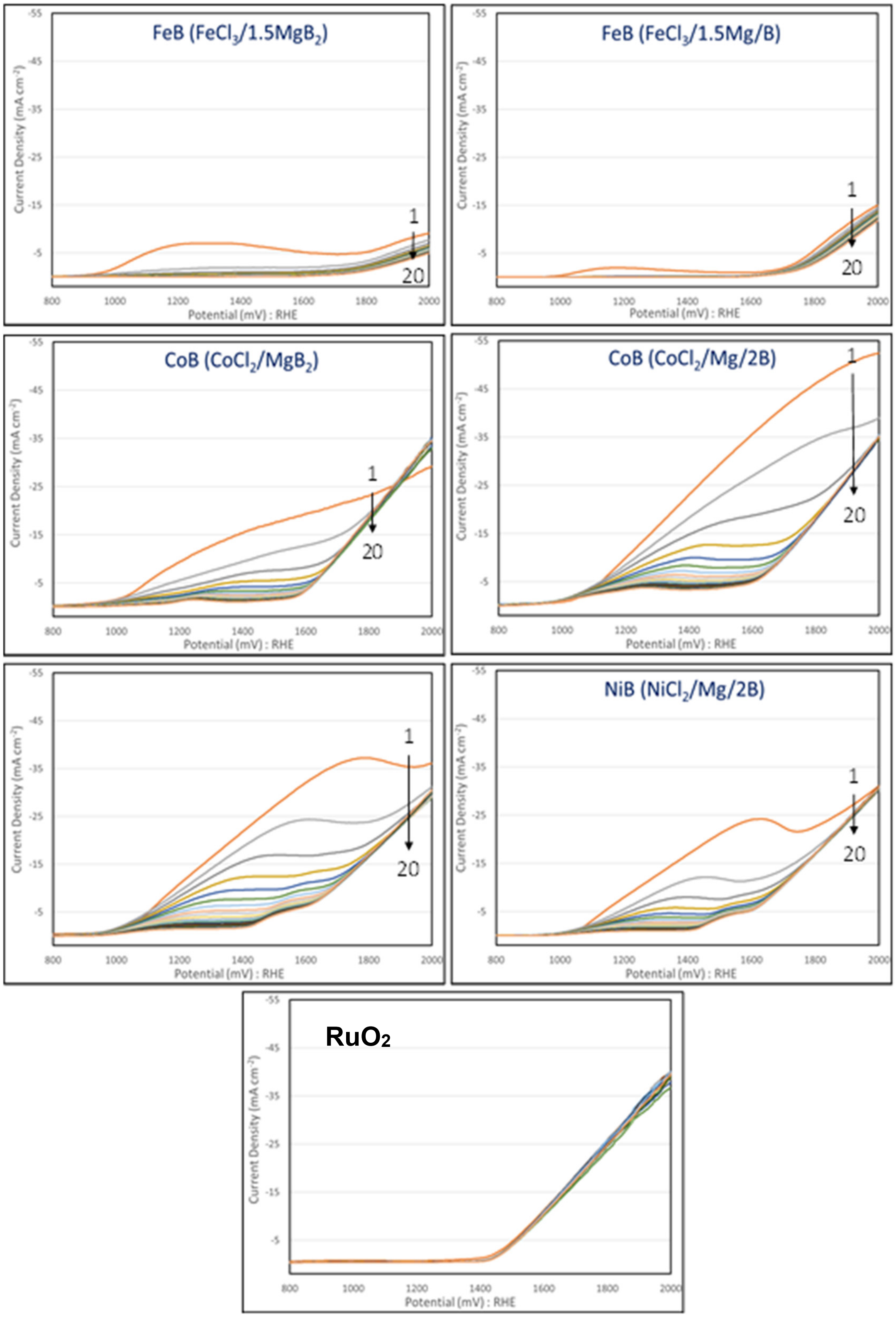

Figure S21. Overlays of first 20 conditioning LSV runs for OER experiments in 0.1 M KOH at 5 mV/s scan rate. Scans for a RuO_2 powder standard are also included. The working electrodes are metal boride powders embedded on C_{wax} tips. LSV curves moved from run 1 to run 20 as represented by the arrow.

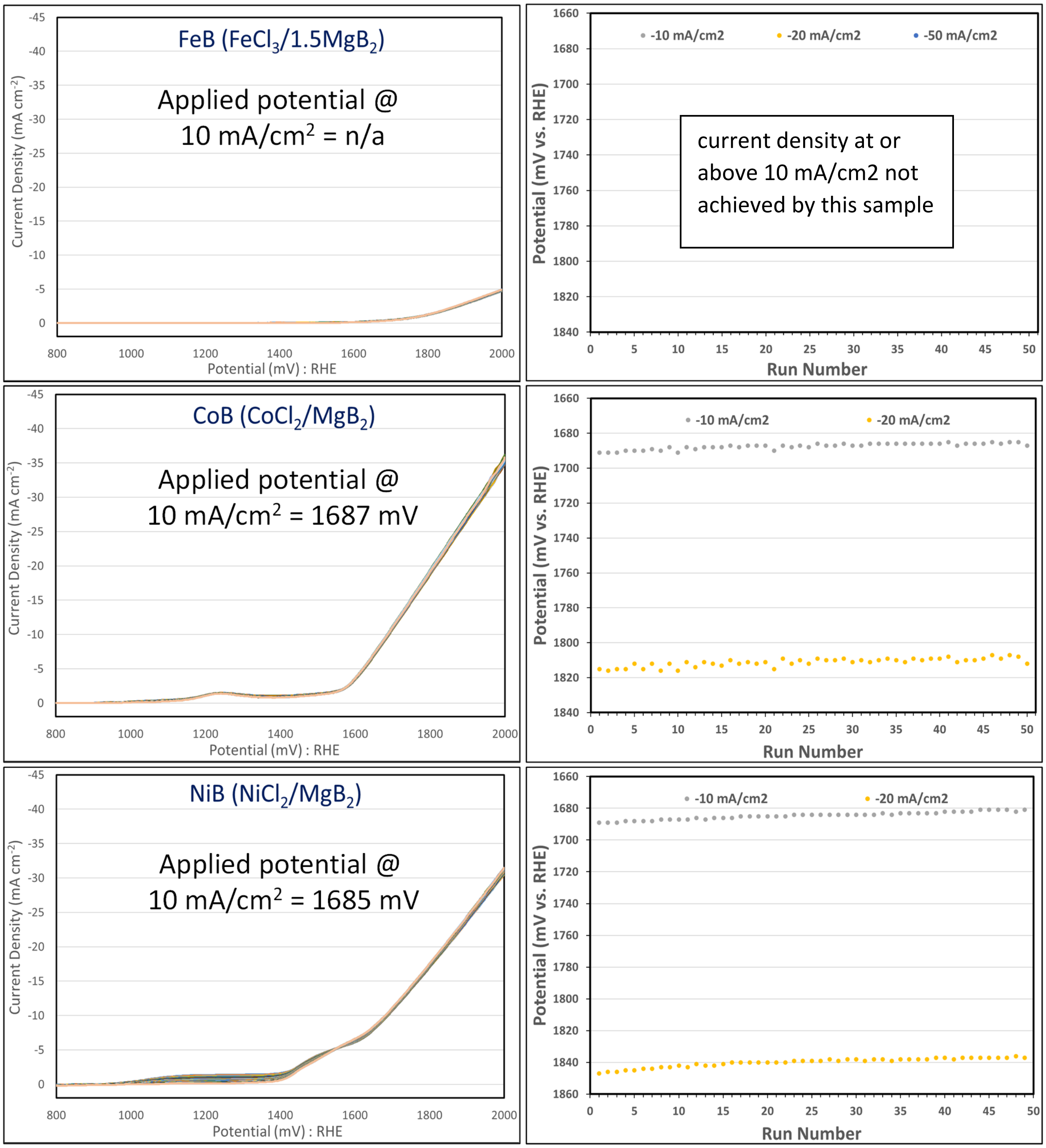

Figure S22A. Left column: Overlay plots of 50 LSV runs of MBs formed from MCl_x/MgB_2 reactions for OER experiments in 0.1 M KOH at 5 mV/s scan rate. Right column: Plots of run number versus potentials at 10 and 20 mA/cm^2 current densities extracted from left column overlay plots. The working electrodes are metal boride powders embedded on C_{wax} tips.

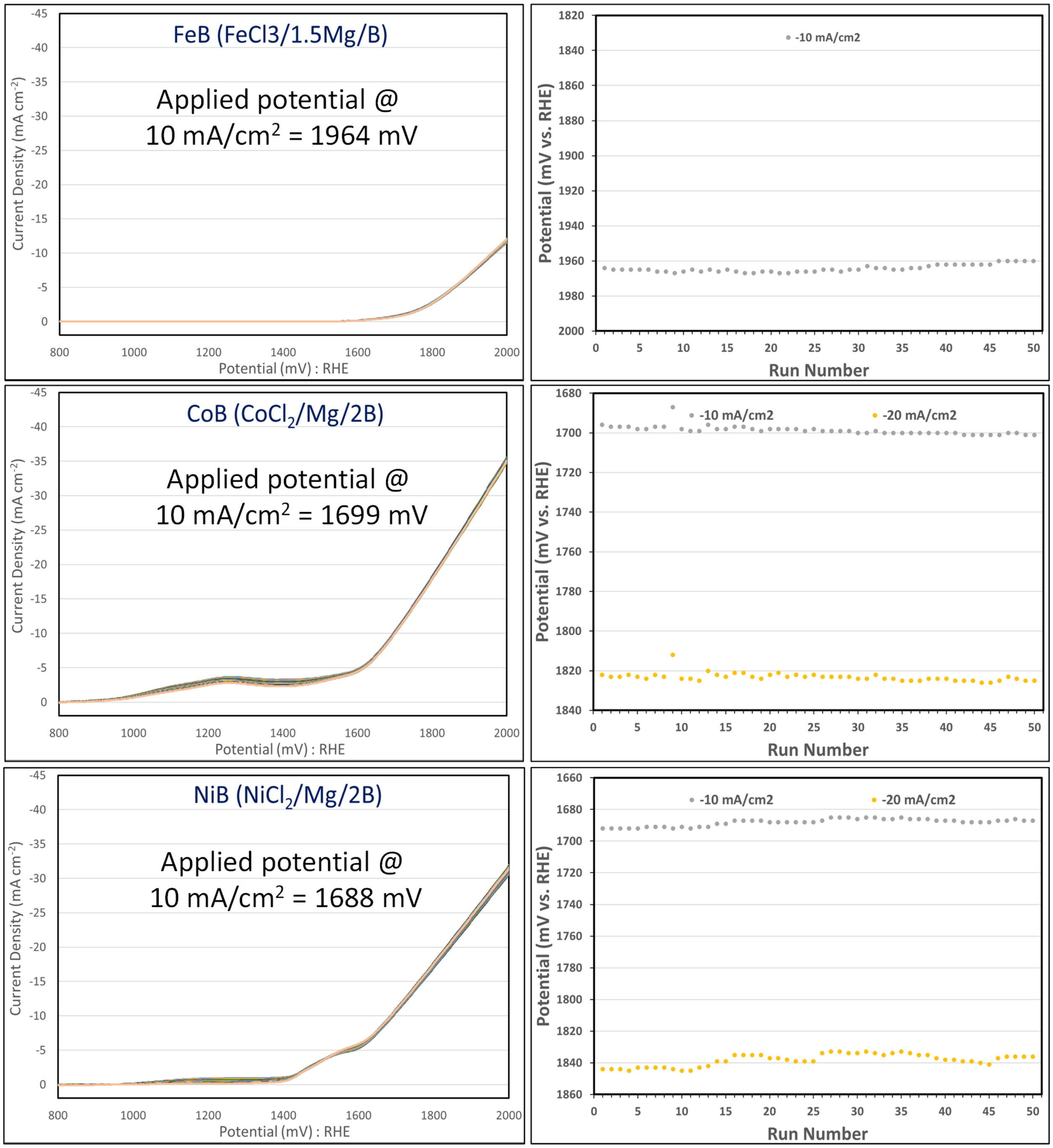

Figure S22B. Left column: Overlay plots of 50 LSV runs of MBs formed from MCl_x/Mg/B reactions for OER experiments in 0.1 M KOH at 5 mV/s scan rate. Right column: Plots of run number versus potentials at 10 and 20 mA/cm^2 current densities extracted from left column overlay plots. The working electrodes are metal boride powders embedded on C_{wax} tips.

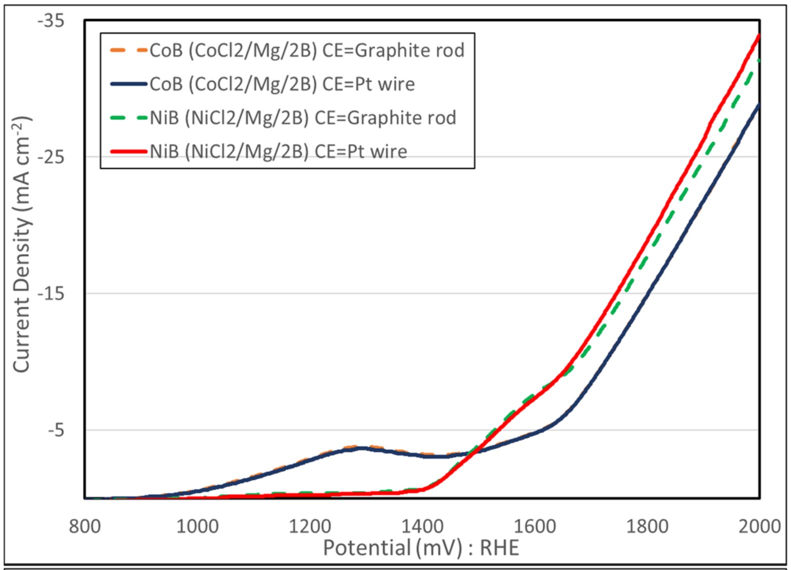

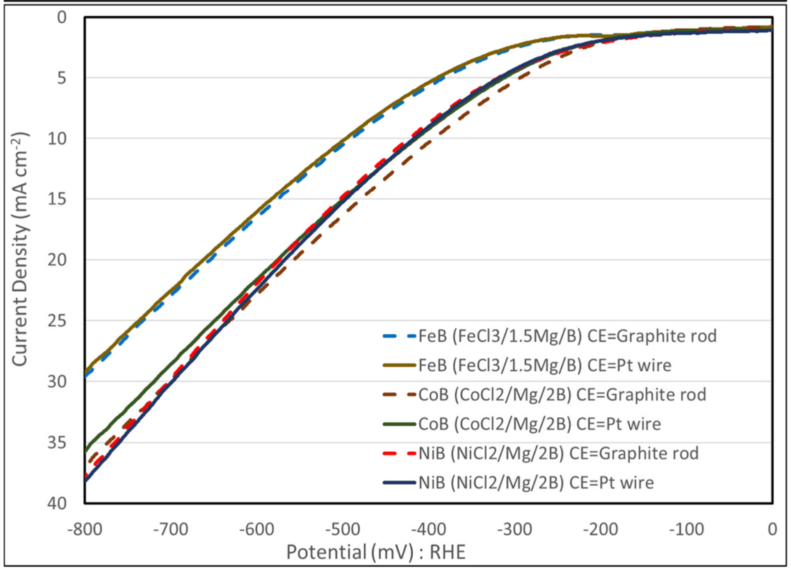

Figure S23. Comparison of CoB and NiB OER activity (top) and FeB, CoB, and NiB HER activity (bottom) in 0.1 M KOH using a Pt counter and a graphite counter electrode.

Table S12. Literature comparison table for **crystalline** metal boride OER and HER activity in different electrolytes. All current densities are normalized for the geometric electrode area. The η_{10} overpotentials are calculated versus the ideal equilibrium 0 V and 1.23 V potentials for HER and OER water splitting.

**MB (M= Fe, Co, Ni)**	**Electrode**	**OER (η_{10} mV)**	**HER (η_{10} mV)**	**Tafel slope (mV/dec) OER/HER**	**Electrolyte**	**Ref.**
FeB ($FeCl_3$/1.5MgB_2)	C_{wax}	418	-368	78/157	1.0 M KOH	this work
FeB ($FeCl_3$/1.5Mg/B)	C_{wax}	480	-361	116/155	1.0 M KOH	this work
CoB ($CoCl_2$/MgB_2)	C_{wax}	373	-293	54/109	1.0 M KOH	this work
CoB ($CoCl_2$/Mg/2B)	C_{wax}	382	-279	69/100	1.0 M KOH	this work
NiB ($NiCl_2$/MgB_2)	C_{wax}	380	-312	72*/112	1.0 M KOH	this work
NiB ($NiCl_2$/Mg/2B)	C_{wax}	346	-307	67*/111	1.0 M KOH	this work
FeB_2	GC	296	-61	52/88	1.0 M KOH	8
Co_3B	CP	312		53	1.0 M KOH	9
Co_2B	GC	410		63	1.0 M KOH	10
Co_2B		380		45	0.1 M KOH	11
Co_2B	GC		-328	136, 177	1.0 M KOH	11
Co_2B	CP	287		51	1.0 M KOH	9
CoB	CP	340		63	1.0 M KOH	9
Ni_3B			-79	85	0.5 M H_2SO_4	12
NiB	GC	350		60	1.0 M KOH	13
**Other metal borides**						
Nano VB_2	CS		-192	68	0.5 M H_2SO_4	14
Bulk VB_2	CS		-348	126	0.5 M H_2SO_4	14
TiB_2	GC		-1070	196	0.5 M H_2SO_4	15
TiB_2	FTO	560			1.0 M $HClO_4$	16
TiB_2	GC		~-1100		0.5 M H_2SO_4	17
TiB_2-NaNAFT	GC		~-1000	146	0.5 M H_2SO_4	17
TiB_2-BuLi	GC		~-1000	158	0.5 M H_2SO_4	17
ZrB_2	GC		-970	173	0.5 M H_2SO_4	15
MoB/g-C_3N_4	GC		-152 (η_{20})	46	1.0 M KOH	18
MoB_2	CS		-154	49	0.5 M H_2SO_4	19
Mo_2B_5	GC		-740	118	0.5 M H_2SO_4	20
Mo_2B_5-BP treated	GC		-540	101	0.5 M H_2SO_4	20
RuB_2	GC		-28	29	1.0 M KOH	21
RuB_2	GC		-18	39	0.5 M H_2SO_4	21
RuB_2	GC		-35	28	0.5 M H_2SO_4	22
HfB_2	GC		-1050	194	0.5 M H_2SO_4	15
WB_2	GC		-203	65	0.5 M H_2SO_4	22
W_2B_5	GC		-680	115	0.5 M H_2SO_4	20
W_2B_5-BP treated	GC		-210	62	0.5 M H_2SO_4	20

C_{wax} = 45% graphite/55% paraffin wax. GC = glassy carbon, CP = carbon paper, CS = carbon sheet, FTO = fluorinated tin oxide glass. *Approximate values due to pre-oxidation peaks having overlap with OER onset.

Table S13. Literature comparison table for **amorphous** MB OER and HER activity in different electrolytes. All current densities are normalized for the geometric electrode area. The η_{10} overpotentials are calculated versus the ideal equilibrium 0 V and 1.23 V potentials for HER and OER water splitting.

MB	Electrode	OER (η_{10} mV)	HER (η_{10} mV)	Tafel slope (mV/dec) OER/HER	Electrolyte	Ref.
FeB	GC	392 (η_{20})		72	1.0 M KOH	23
Co_3B	GC	350 (η_{20})			1.0 M KOH	24
CoB pellet	-		-251	75	0.5 M KPi	25
CoB	GC		-203	79	0.5 M KPi	26
CoB	GC	344 (η_{20})		72	1.0 M KOH	23
CoB/C	GC	320		75	1.0 M KOH	27
CoB nanosheets	NF	265 (η_{20})		56	1.0 M KOH	28
CoB/NCNT		370		-	0.1 M KOH	29
CoB	Ni	140	-70	89/68	1.0 M KOH	29
Co-B@Co-Bi	GC	291		105	1.0 M KOH	30
CoB	GC	348		111	1.0 M KOH	30
Ni_3B-rGO films	CFP	290		88	1.0 M KOH	31
Ni_3B	CFP	340		81	1.0 M KOH	31
Ni_2B	GC	350		58	1.0 M KOH	32
NiB	GC		-132 (η_{20})	53, 112	1.0 M $HClO_4$	33
NiB	GC		-194 (η_{20})		1.0 M KOH	33
NiB	GC		-309	186	0.5 M KPi	26
Ni_2B/g-C_3N_4	GC		-707	221	1.0 M KOH	34
Ni-B@Ni-Bi	GC	310		150	1.0 M KOH	30
NiB	GC	365		100	1.0 M KOH	30
NiB	GC	331 (η_{20})		52	1.0 M KOH	23

GC = glassy carbon, CFP = carbon fiber paper, NF = nickel foam

Table S14. Summary of OER electrocatalysis with SSM synthesized metal borides in 1.0 M KOH.

**Sample**	**Applied potential @ 10 mA/cm^2 (mV)**[1]	**Applied potential @ 20 mA/cm^2 (mV)**	**Tafel (mV/dec)**
RuO_2	1524 ± 5<br>(1512 ± 1)	1581 ± 7<br>(1538 ± 1)	65
FeB ($FeCl_3$/1.5MgB_2)	1648 ± 2	1706 ± 2	78
FeB ($FeCl_3$/1.5Mg/B)	1710 ± 4	1784 ± 5	116
CoB ($CoCl_2$/MgB_2)	1603 ± 2	1644 ± 3	54
CoB ($CoCl_2$/Mg/2B)	1612 ± 1<br>(1577 ± 3)	1656 ± 1<br>(1613 ± 4)	69
NiB ($NiCl_2$/MgB_2)	1610 ± 5	1672 ± 5	72
NiB ($NiCl_2$/Mg/2B)	1576 ± 5<br>(1521 ± 1)	1633 ± 2<br>(1597 ± 1)	67

1) Applied potentials reported versus RHE and current densities normalized to geometric electrode area of 0.08 cm^2 (85% iR compensation results in parentheses). Deviations are for 50 LSV runs or 10 LSV runs at 85% iR compensation.

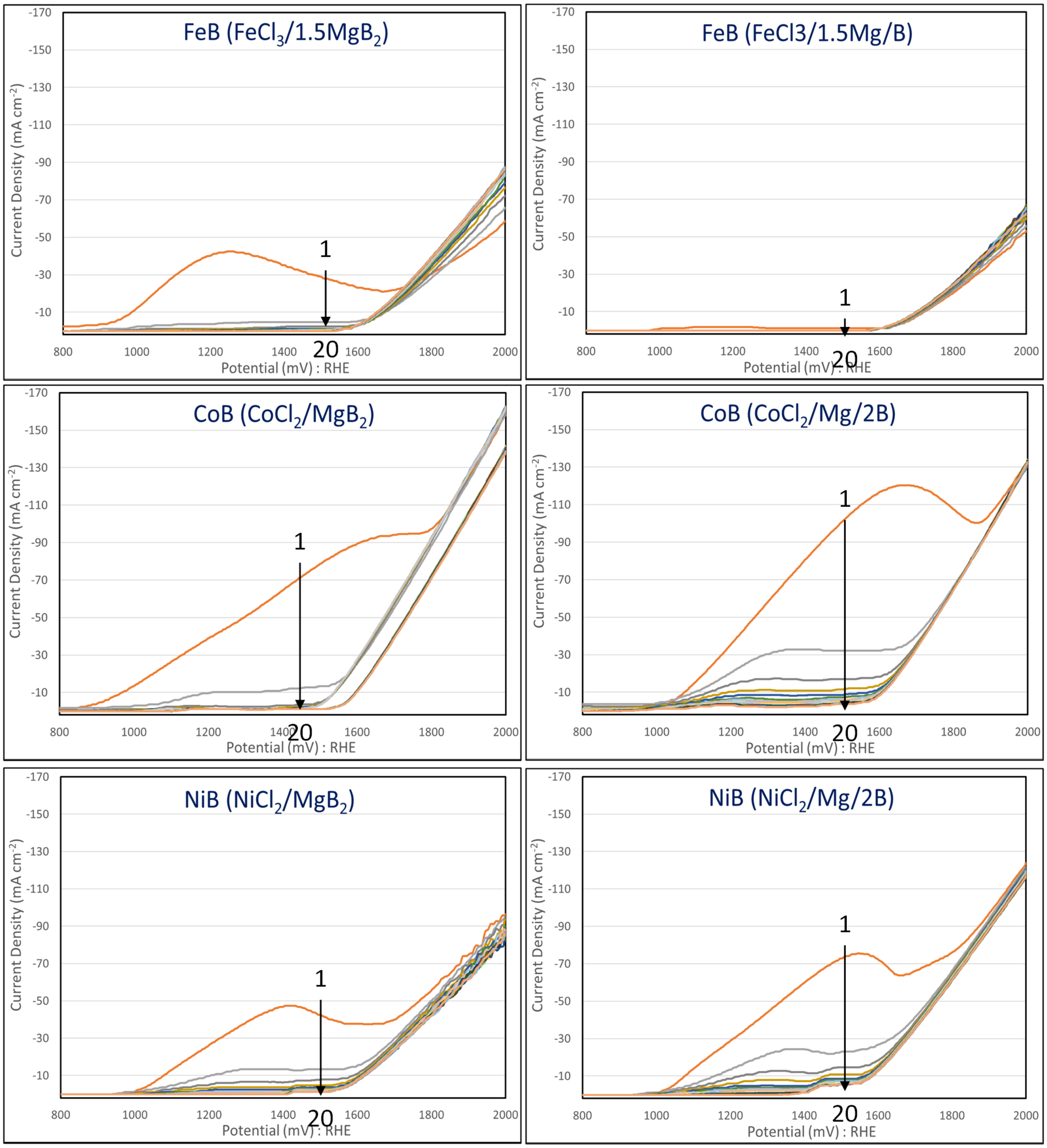

Figure S24. Overlays of first 20 conditioning LSV runs for OER experiments in 1.0 M KOH at 5 mV/s scan rate. The working electrodes are metal boride powders embedded on C_{wax} tips. LSV curves moved from run 1 to run 20 as represented by the arrow.

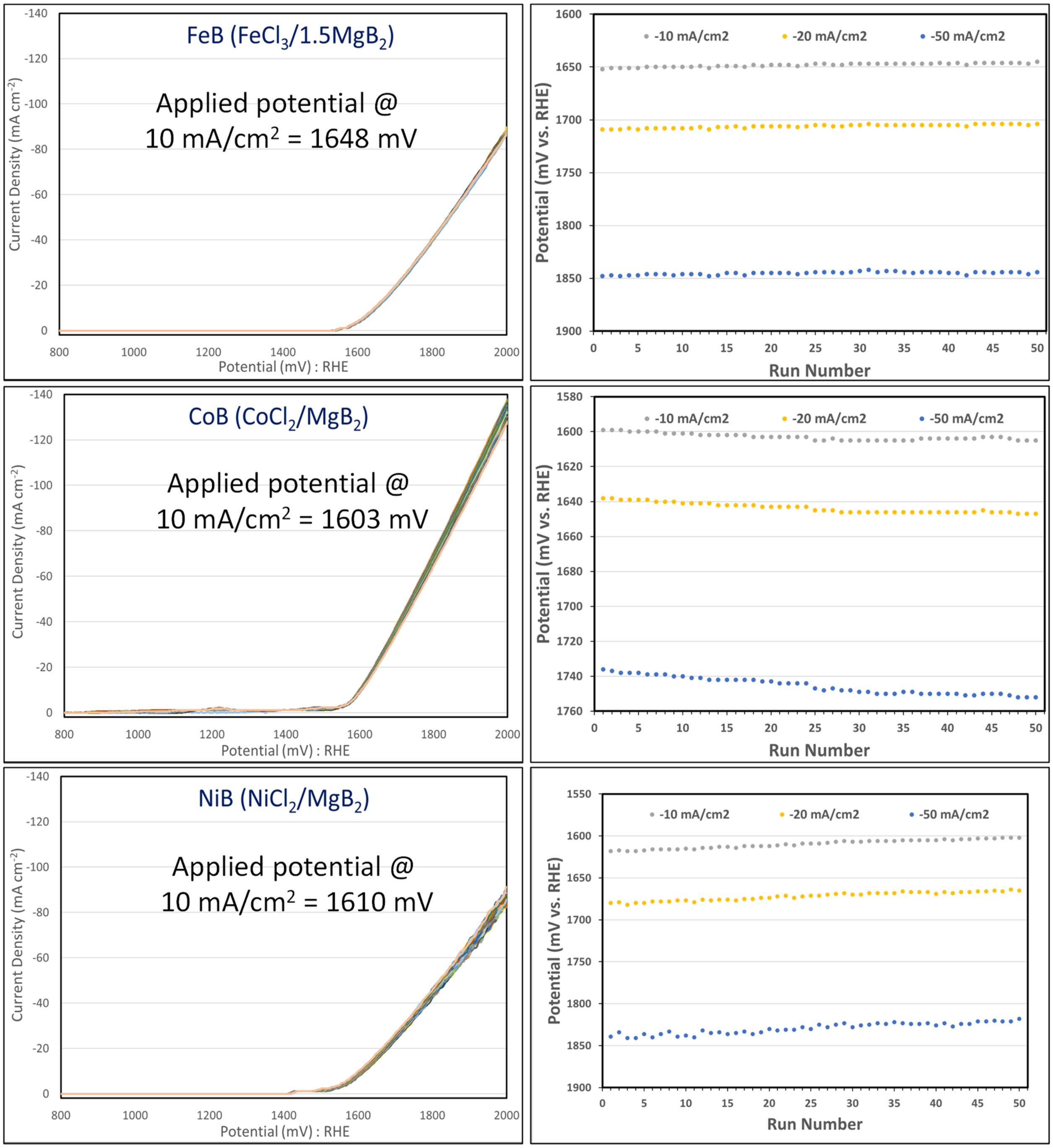

Figure S25A. Left column: Overlay plots of 50 LSV runs of MBs formed from MCl_x/MgB_2 reactions for OER experiments in 1.0 M KOH at 5 mV/s scan rate. Right column: Plots of run number versus potentials at 10, 20, and 50 mA/cm^2 current densities extracted from left column overlay plots. The working electrodes are metal boride powders embedded on C_{wax} tips.

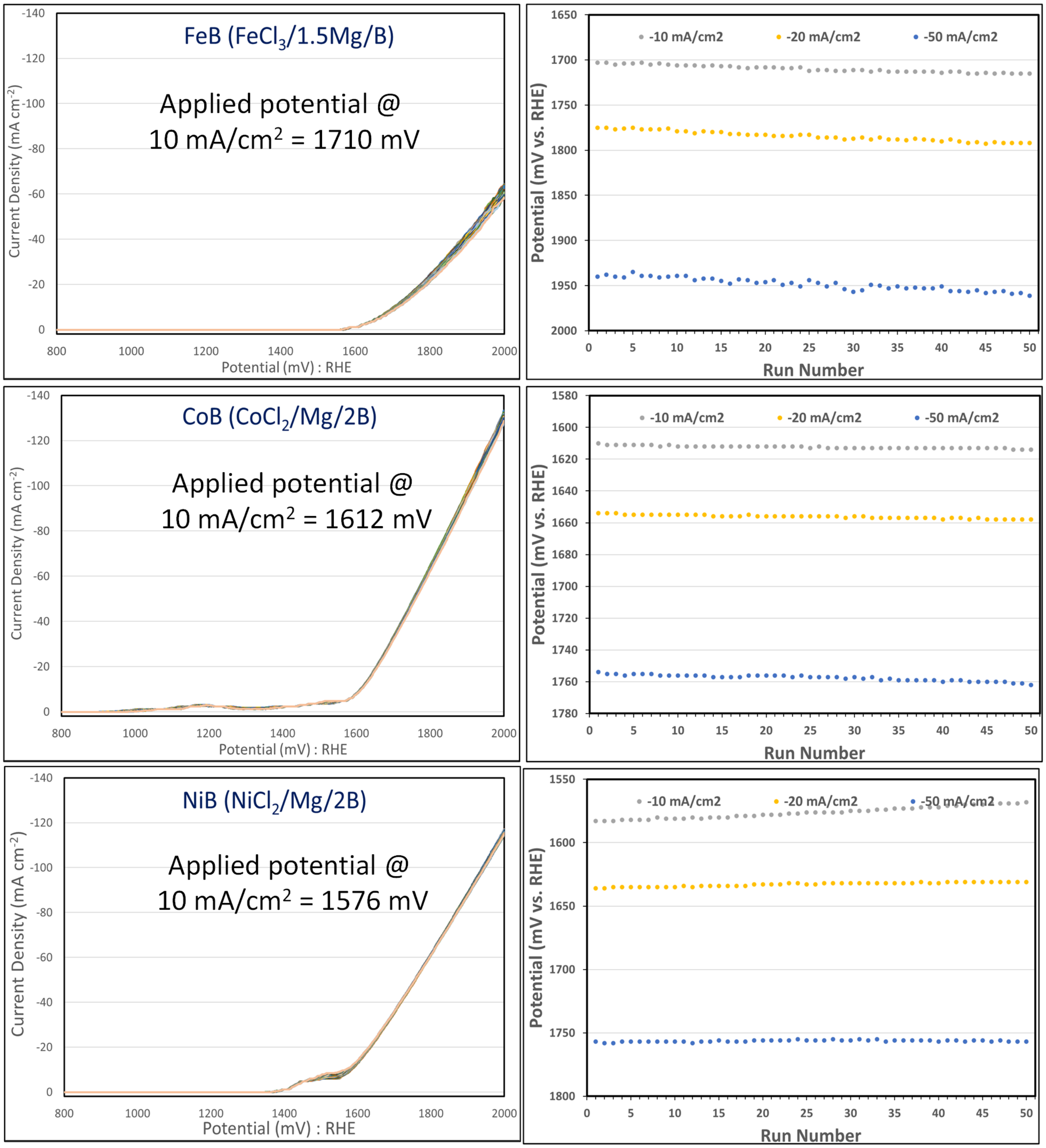

Figure S25B. Left column: Overlay plots of 50 LSV runs of MBs formed from MCl_x/Mg/B reactions for OER experiments in 1.0 M KOH at 5 mV/s scan rate. Right column: Plots of run number versus potentials at 10, 20, and 50 mA/cm^2 current densities extracted from left column overlay plots. The working electrodes are metal boride powders embedded on C_{wax} tips.

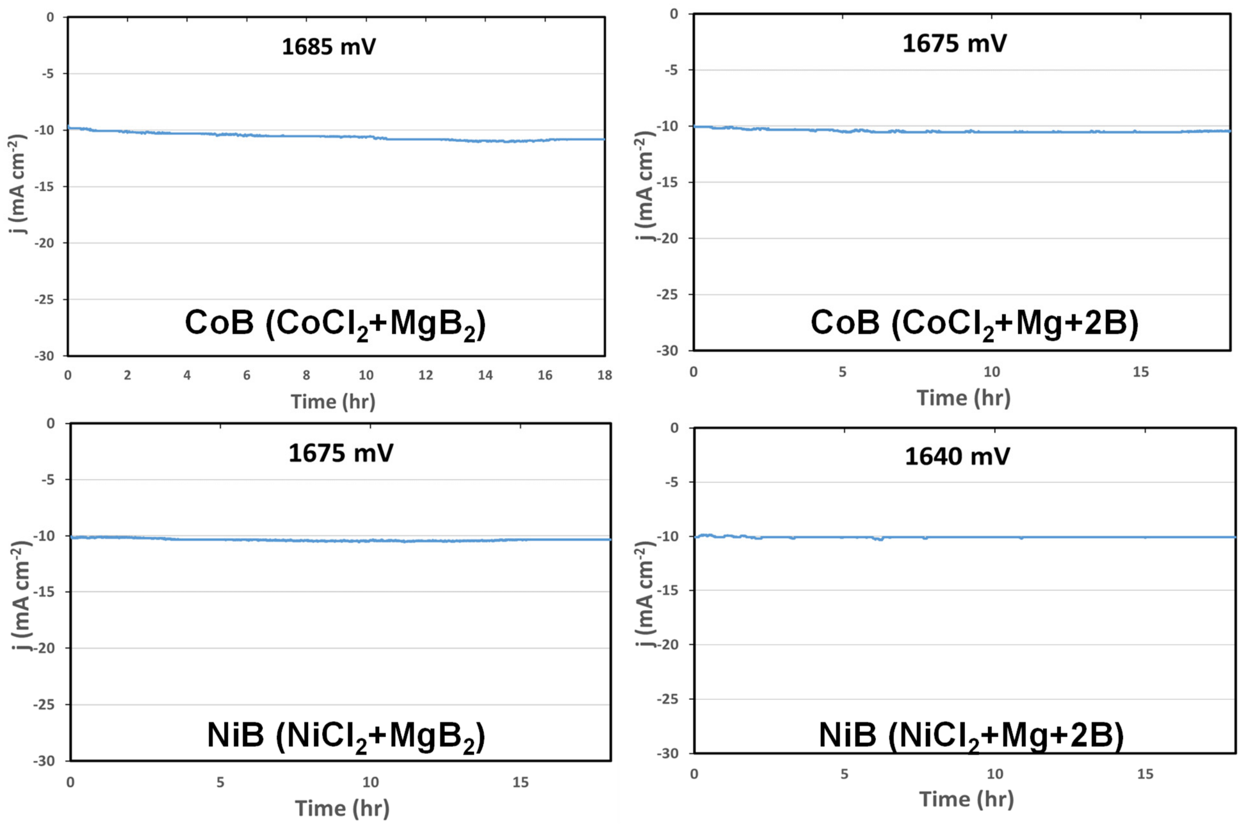

Figure S26. Applied positive potential OER chronoamperometry data (current versus time at constant potential) for metal boride synthesized from MgB_2 and Mg/B SSM reactions in 0.1 M KOH for 18 hrs. The potentials used are indicated and were chosen to ideally sustain 10 mA/cm^2. The working electrodes are metal boride powders embedded on C_{wax} tips.

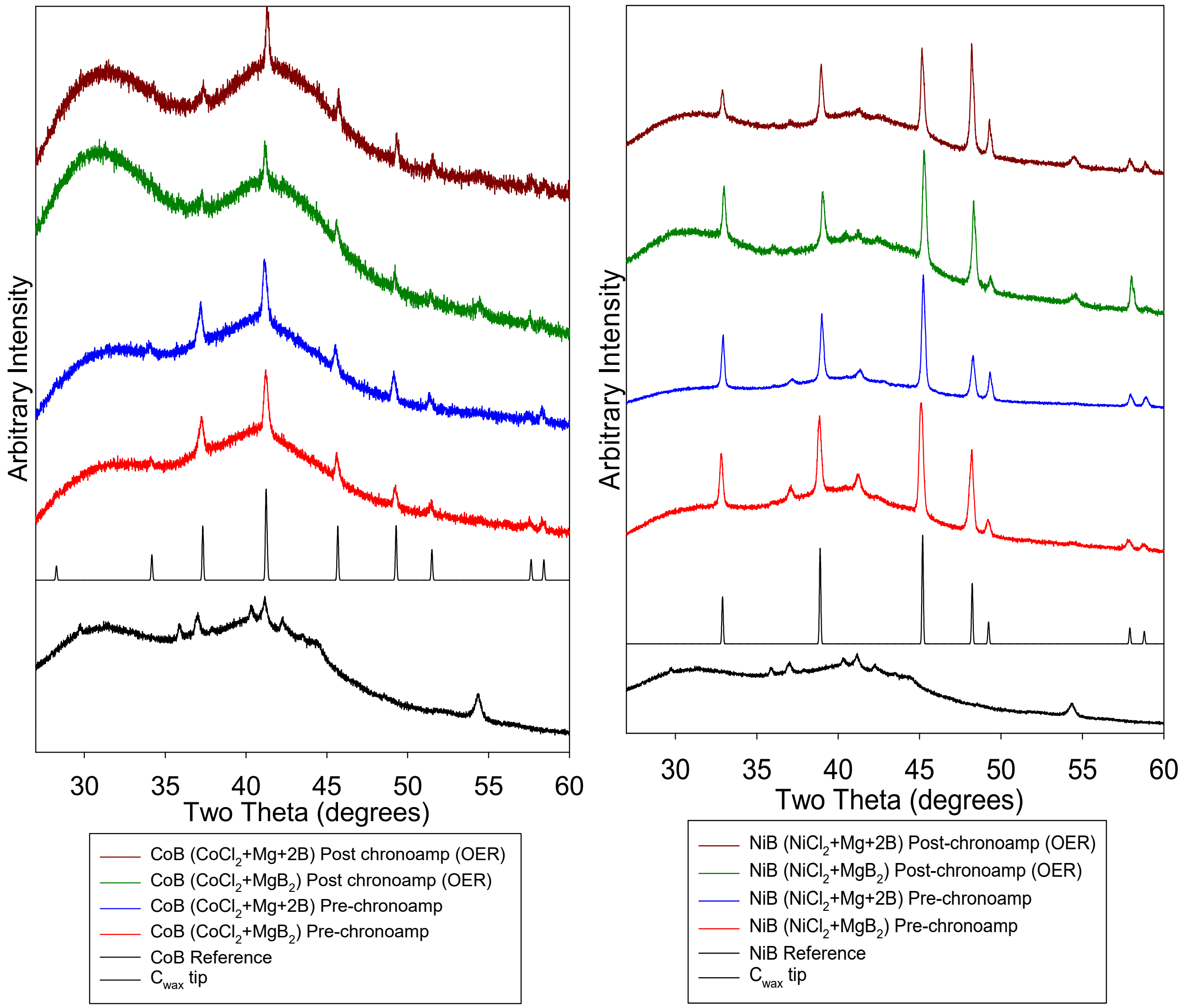

Figure S27. Powder XRD results of pre- and post-positive potential OER chronoamperometry (0.1 M KOH) of powders on C_{wax} tips for CoB and NiB samples formed by MCl_2+MgB_2 and MCl_2+Mg+2B reactions. Reference patterns for NiB, CoB, and diffractogram of C_{wax} are also shown. The C_{wax} electrode has several broad XRD peaks that are a combination of graphite and paraffin wax diffraction.

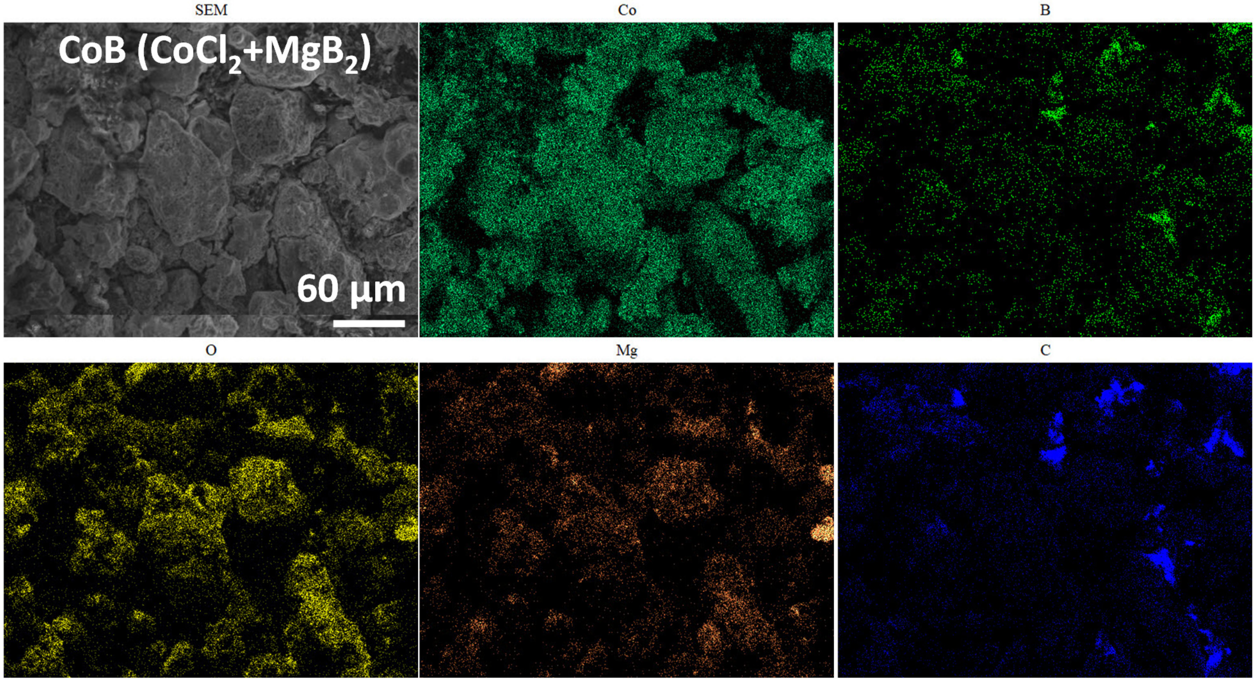

Figure S28. Post-positive potential OER chronoamperometry EDS maps of CoB formed from $CoCl_2$+MgB_2 reaction. Images are from powders embedded on C_{wax} tip.

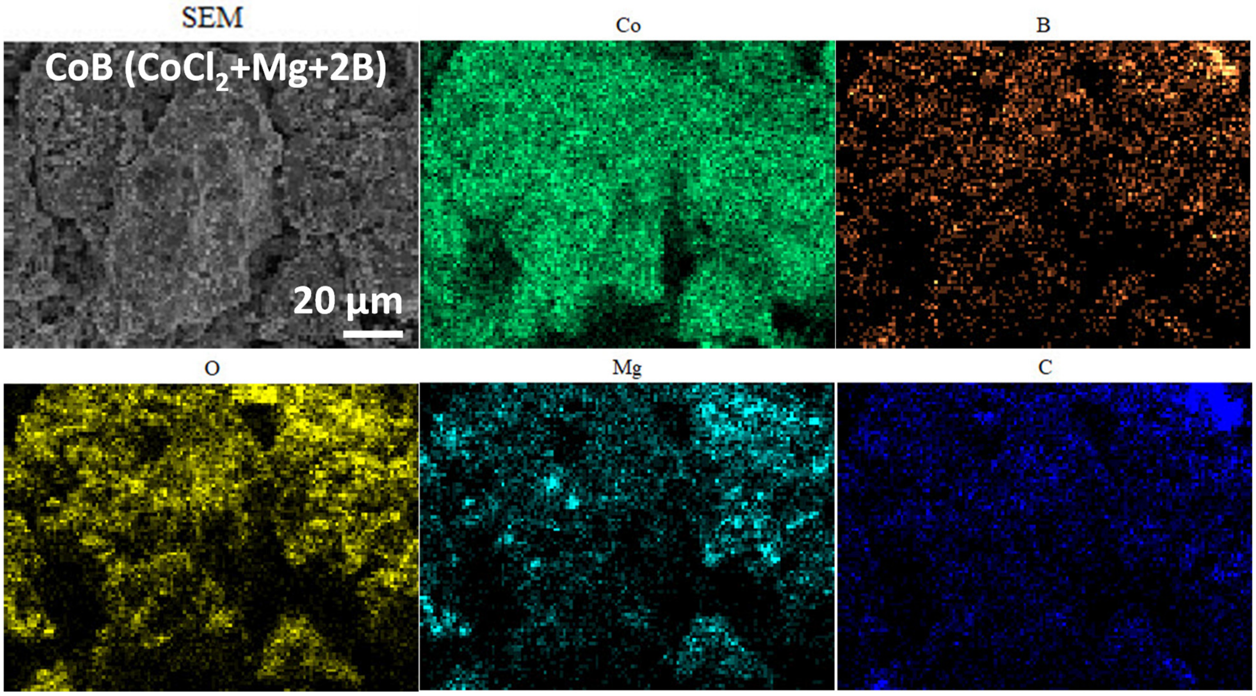

Figure S29. Post-positive potential OER chronoamperometry EDS maps of CoB formed from $CoCl_2$+Mg+2B reaction. Images are from powders embedded on C_{wax} tip.

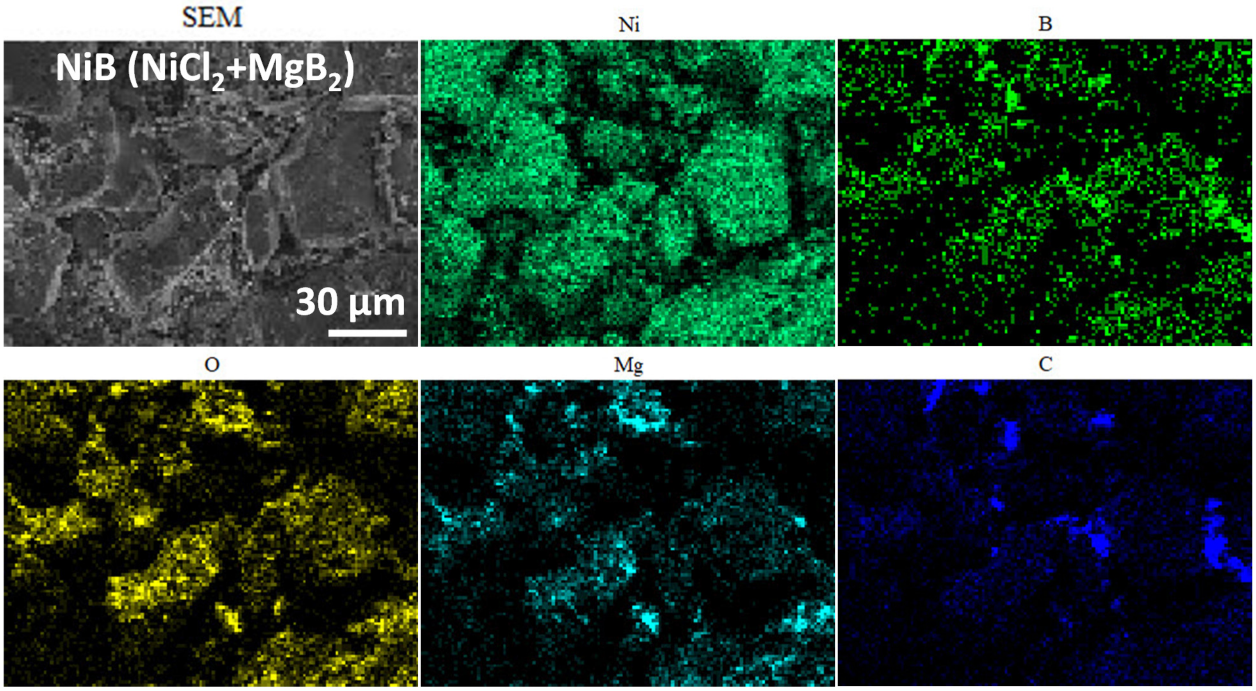

Figure S30. Post-positive potential OER chronoamperometry EDS maps of NiB formed from $NiCl_2$+MgB_2 reaction. Images are from powders embedded on C_{wax} tip.

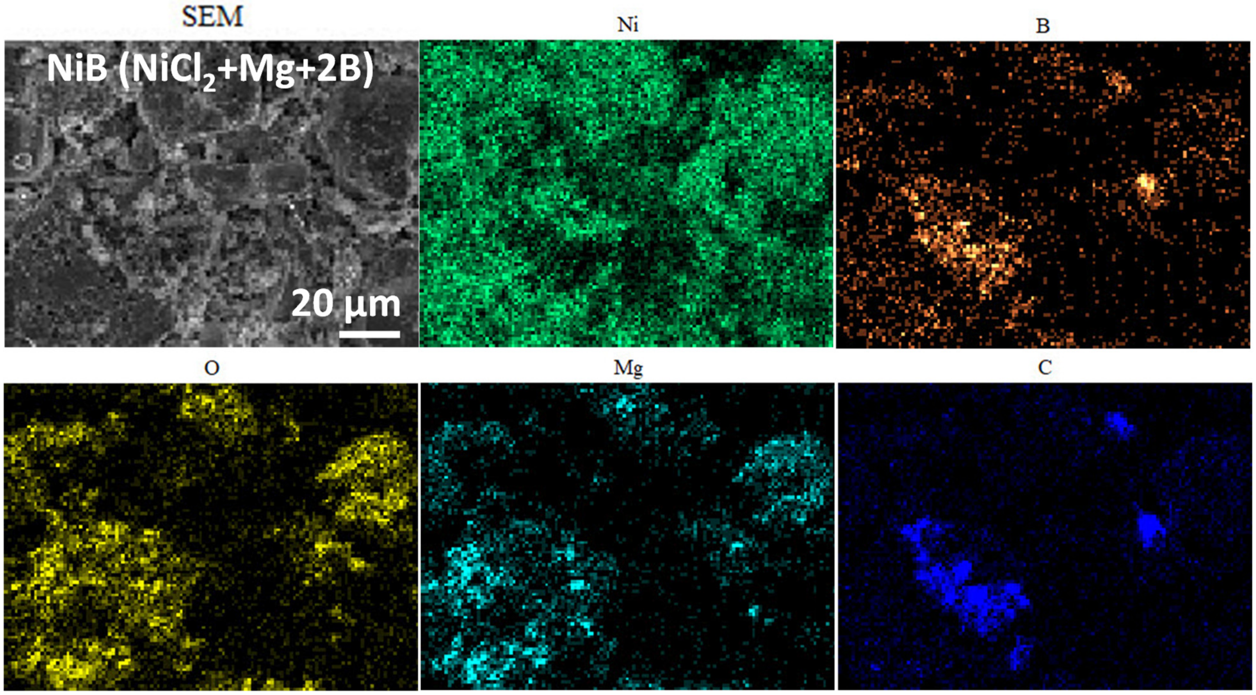

Figure S31. Post-positive potential OER chronoamperometry EDS maps of NiB formed from $NiCl_2$+Mg+2B reaction. Images are from powders embedded on C_{wax} tip.

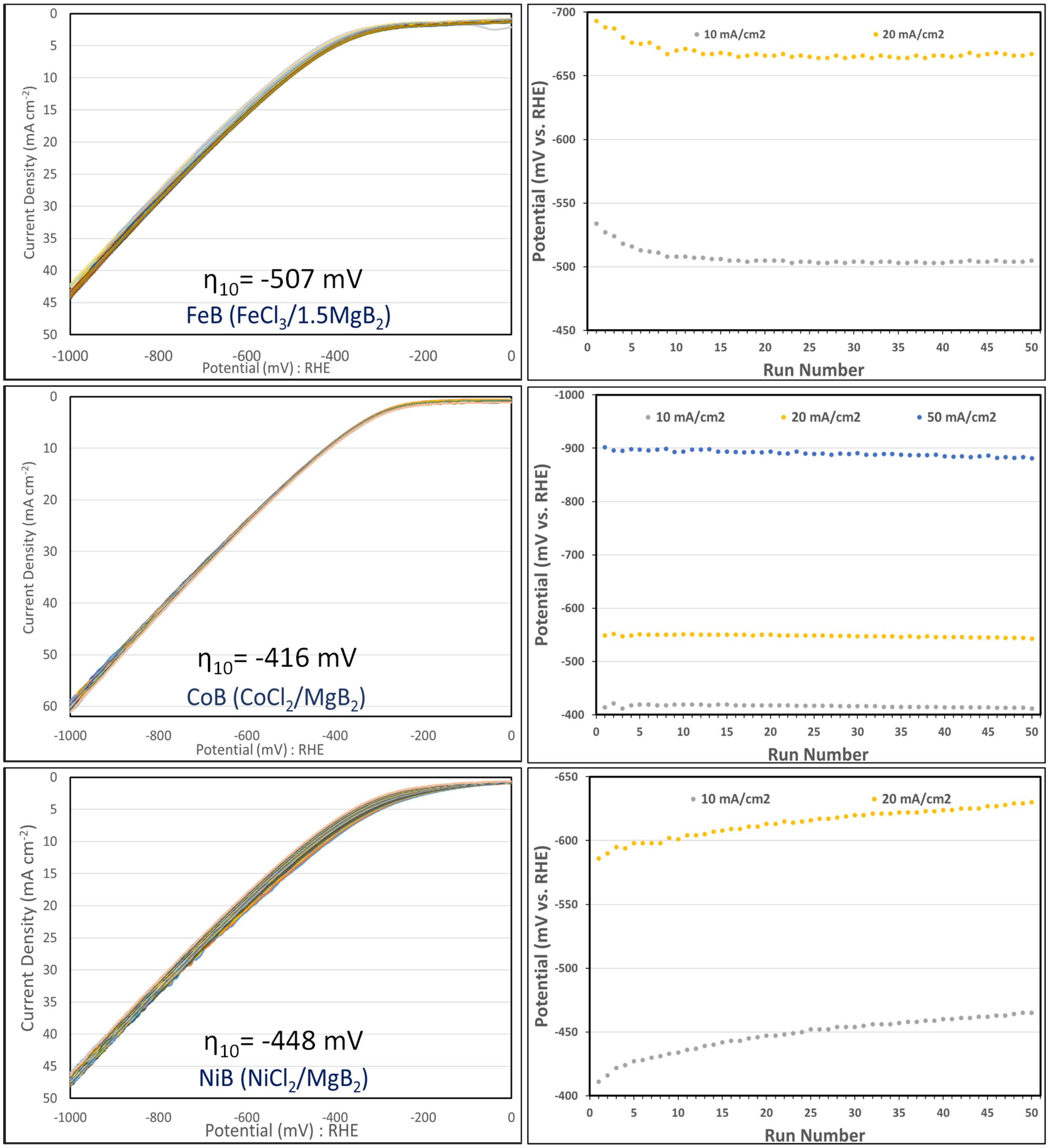

Figure S32A. Left column: Overlay plots of 50 LSV scans of MBs formed from MCl_x/MgB_2 reaction for HER in 0.1 M KOH at 5 mV/s scan rate. Right column: Plots of run number versus potentials at 10 and 20 mA/cm^2 current densities extracted from left column overlay plots. The working electrodes are metal boride powders embedded on C_{wax} tips.

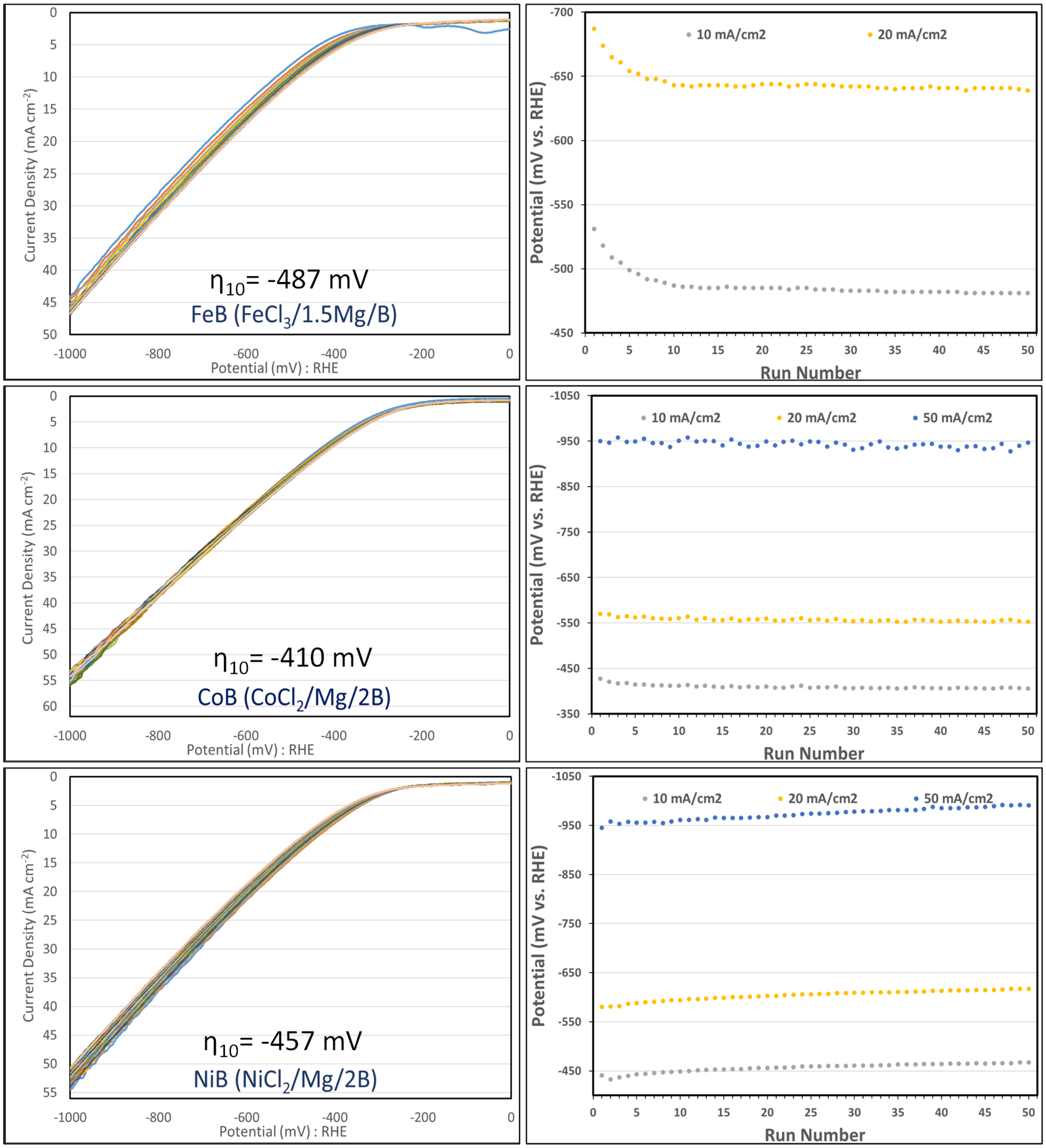

Figure S32B. Left column: Overlay plots of 50 LSV scans of MBs formed from MCl_x/Mg/B reaction for HER in 0.1 M KOH at 5 mV/s scan rate. Right column: Plots of run number versus potentials at 10 and 20 mA/cm^2 current densities extracted from left column overlay plots. The working electrodes are metal boride powders embedded on C_{wax} tips.

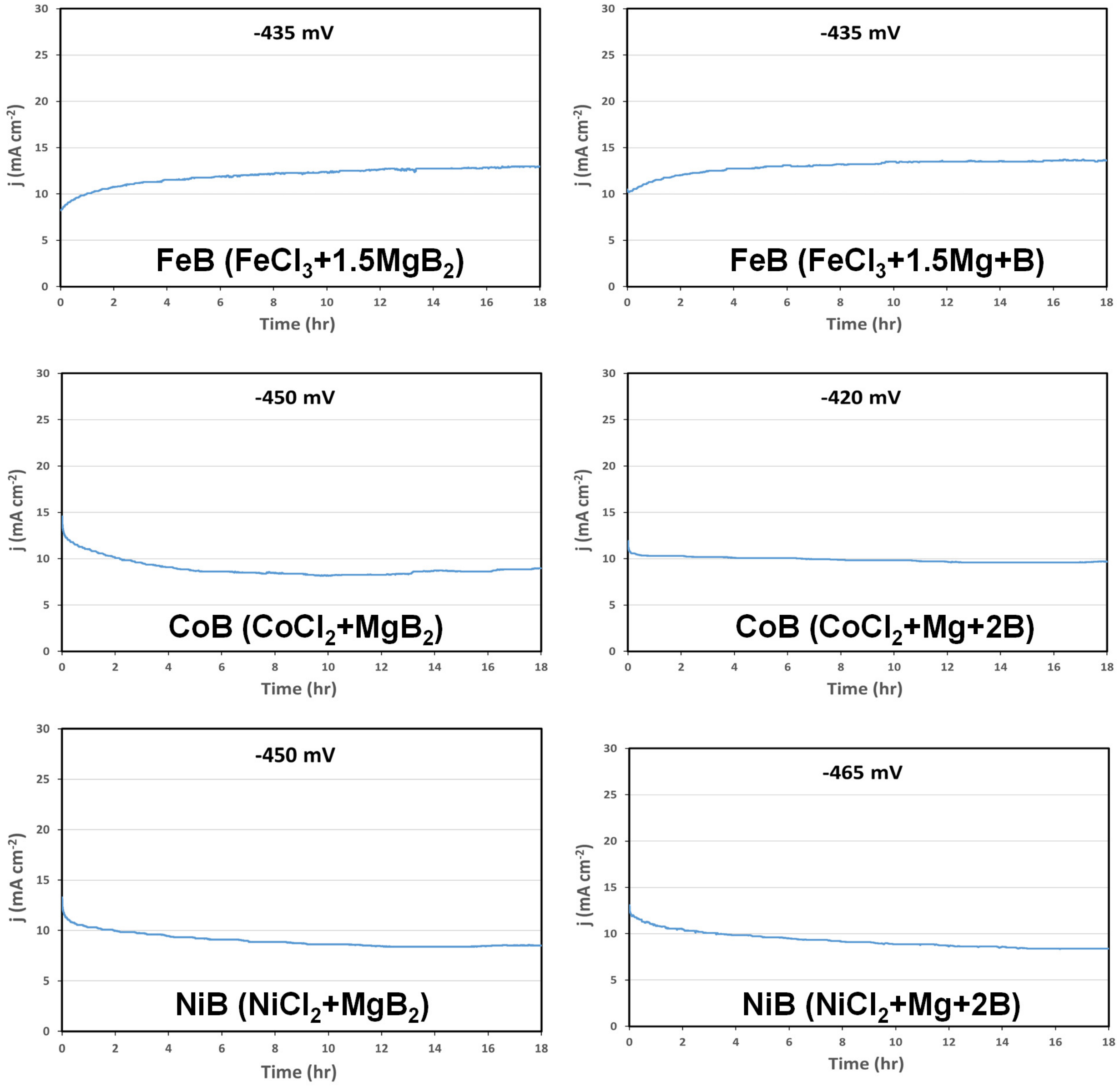

Figure S33. Negative potential HER chronoamperometry data (current versus time at constant potential) of metal borides synthesized from MgB_2 and Mg/B SSM reactions in 0.1 M KOH for 18 hrs. The potentials used are indicated in boxes and were chosen to ideally sustain 10 mA/cm^2. The working electrodes are metal boride powders embedded on C_{wax} tips.

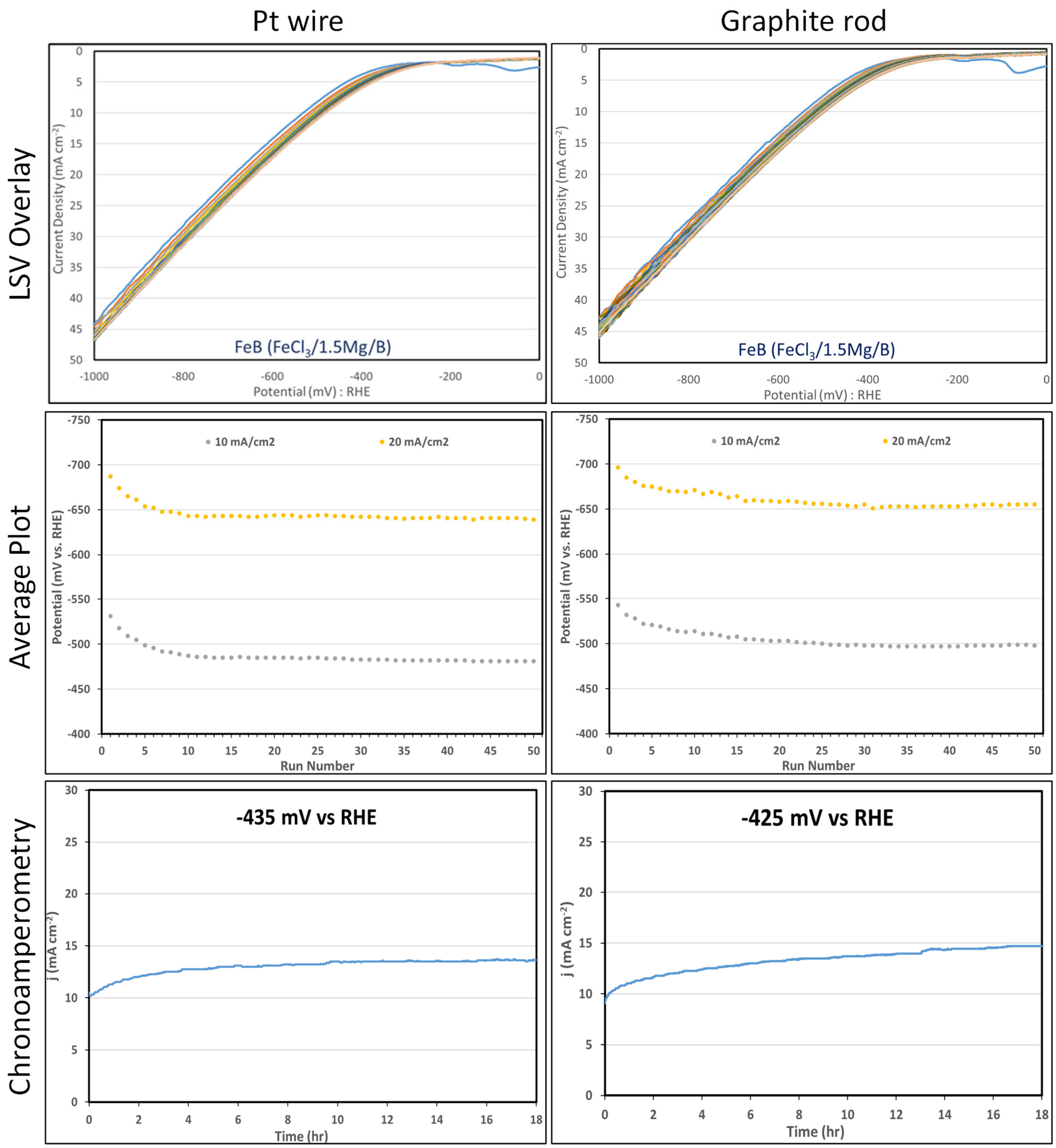

Figure S34. The comparison of HER activity and stability of FeB ($FeCl_3$/1.5Mg/B) in 0.1 M KOH with graphite versus platinum wire counter electrodes. Average plots represent the run number versus potentials at 10 and 20 mA/cm^2 current densities extracted from 50 HER overlay plots.

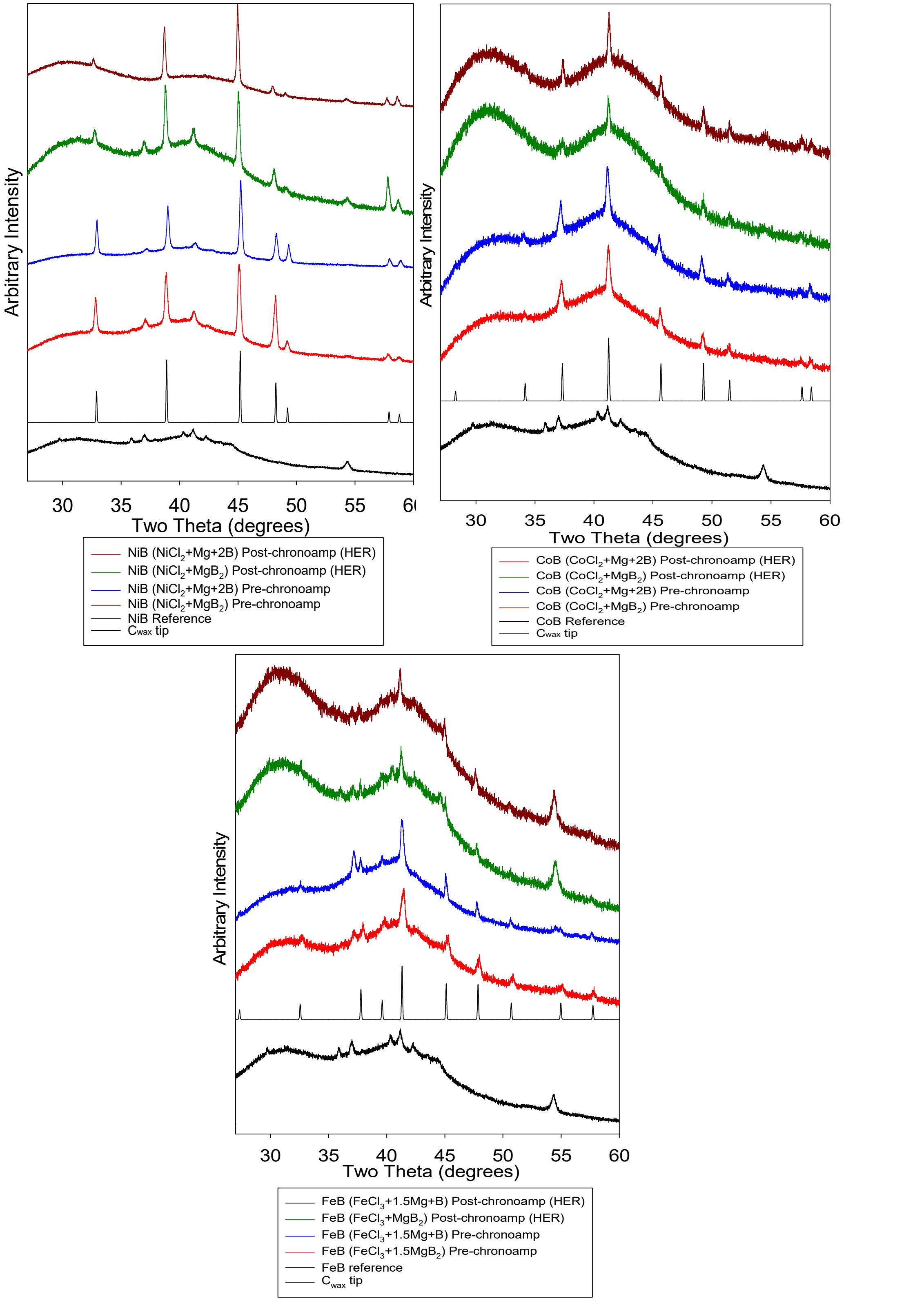

Figure S35. Powder XRD results of post-negative potential HER chronoamperometry of NiB, CoB, and FeB samples form from MCl_x/MgB_2 and MCl_x/Mg/B reactions. Reference patterns for NiB, CoB, and FeB and diffractogram of C_{wax} are also shown. Data are obtained from powders on C_{wax} tips.

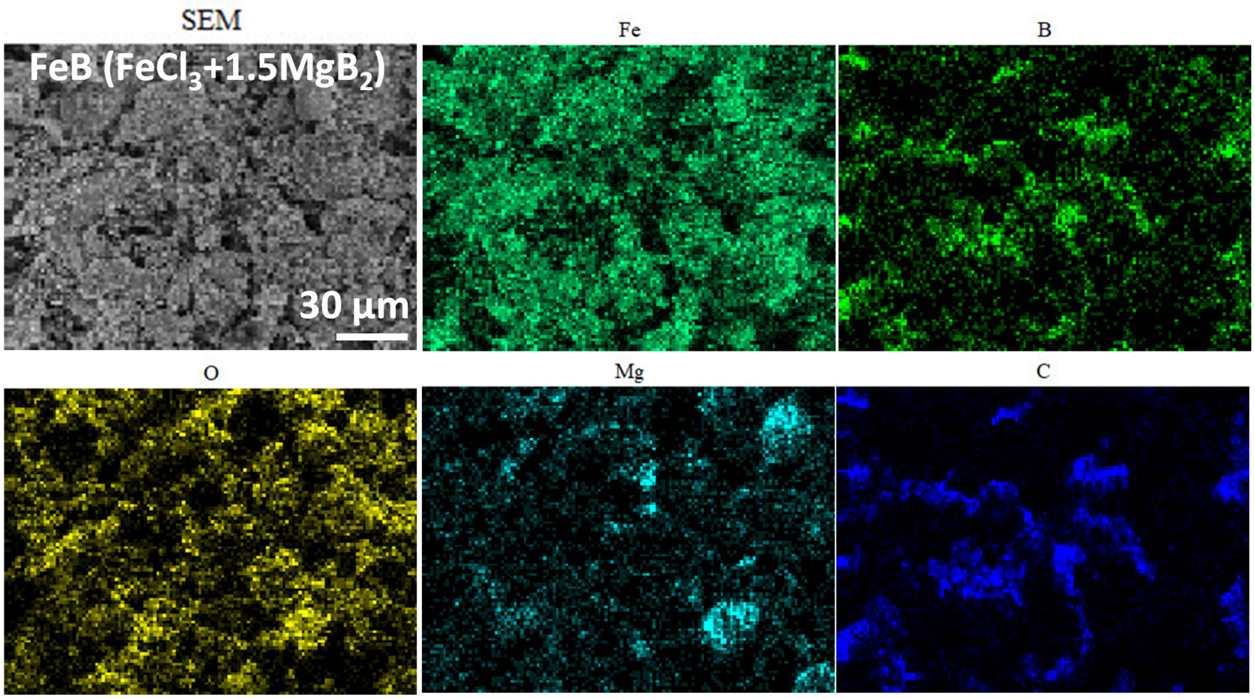

Figure S36. Post-negative potential HER chronoamperometry EDS maps of FeB formed from $FeCl_3$+1.5MgB_2 reaction. Images are from powders embedded on C_{wax} tip.

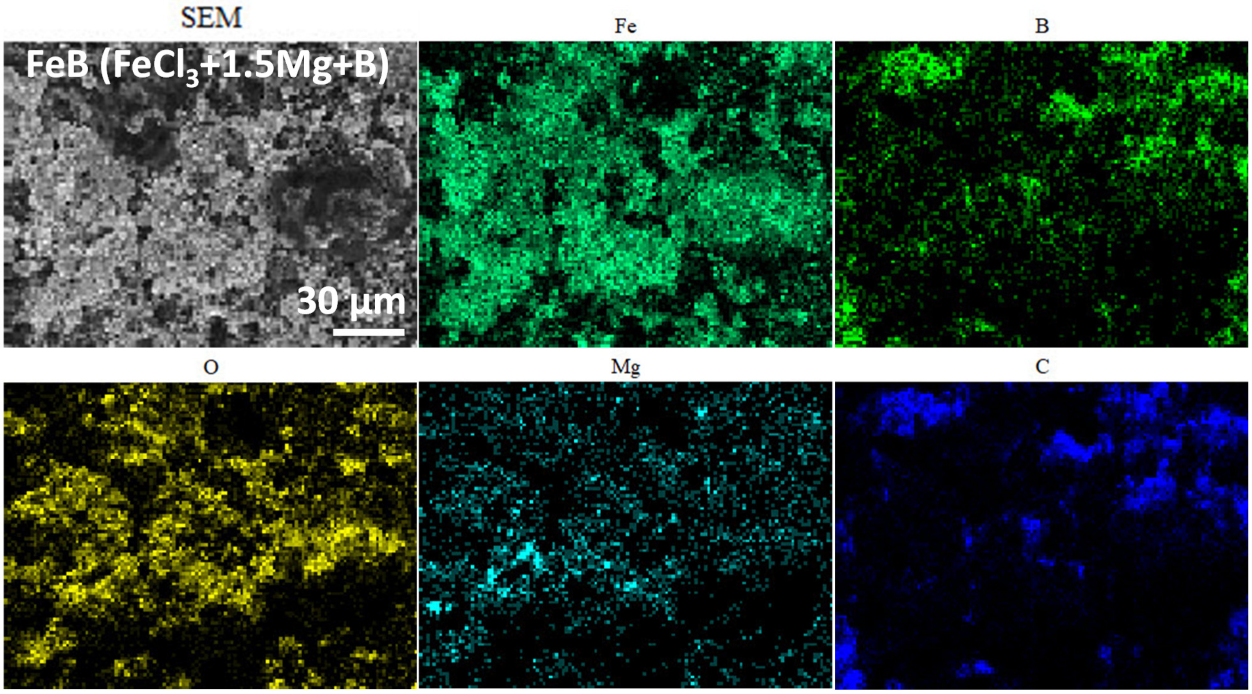

Figure S37. Post-negative potential HER chronoamperometry EDS maps of FeB formed from $FeCl_3$+1.5Mg+B reaction. Images are from powders embedded on C_{wax} tip.

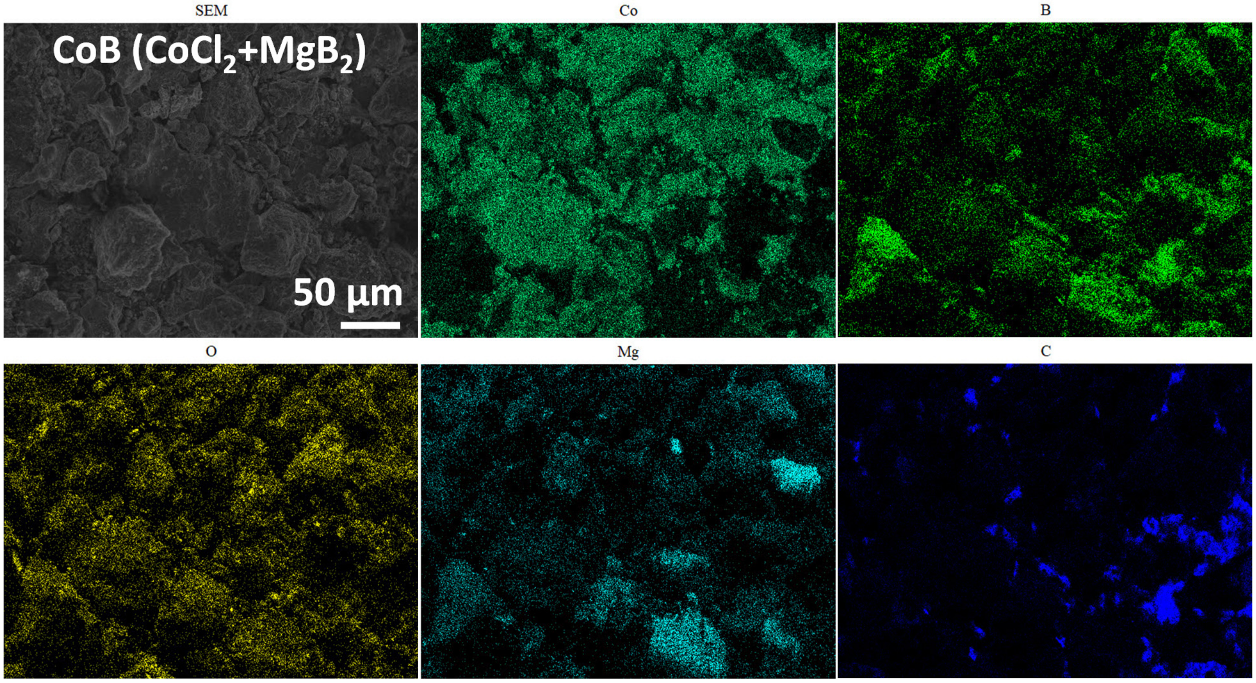

Figure S38. Post-negative potential HER chronoamperometry EDS maps of CoB formed from $CoCl_2$+MgB_2 reaction. Images are from powders embedded on C_{wax} tip.

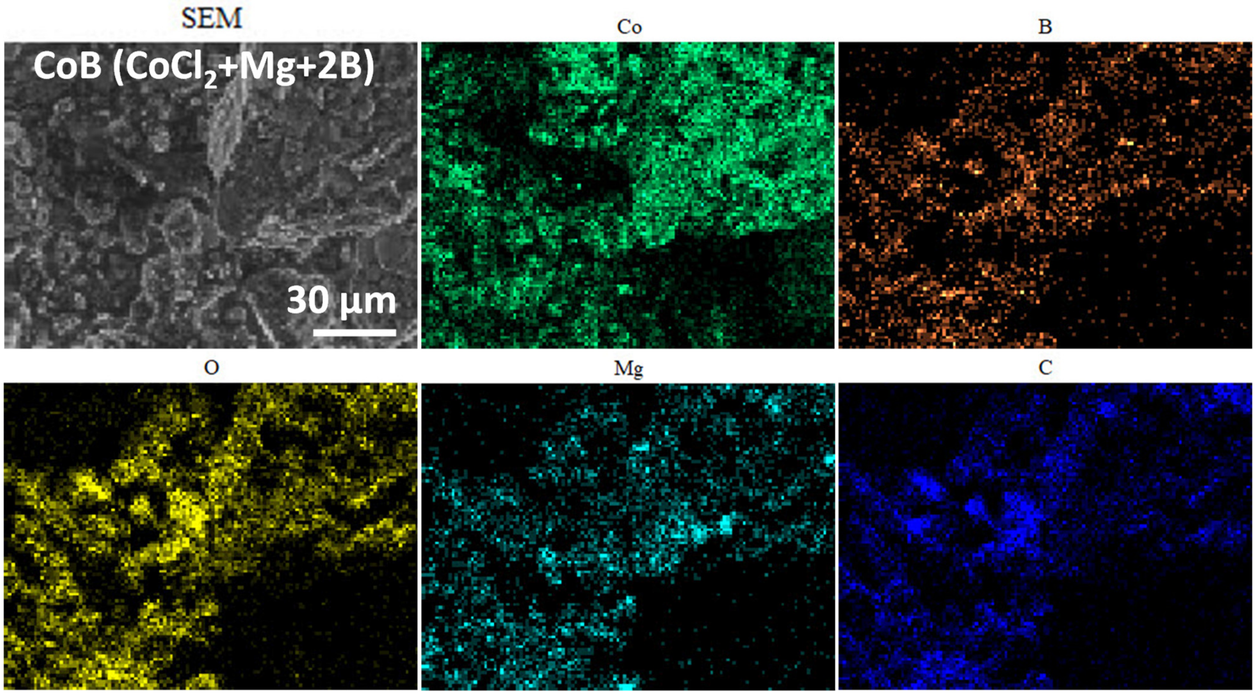

Figure S39. Post-negative potential HER chronoamperometry EDS maps of CoB formed from $CoCl_2$+Mg+2B reaction. Images are from powders embedded on C_{wax} tip.

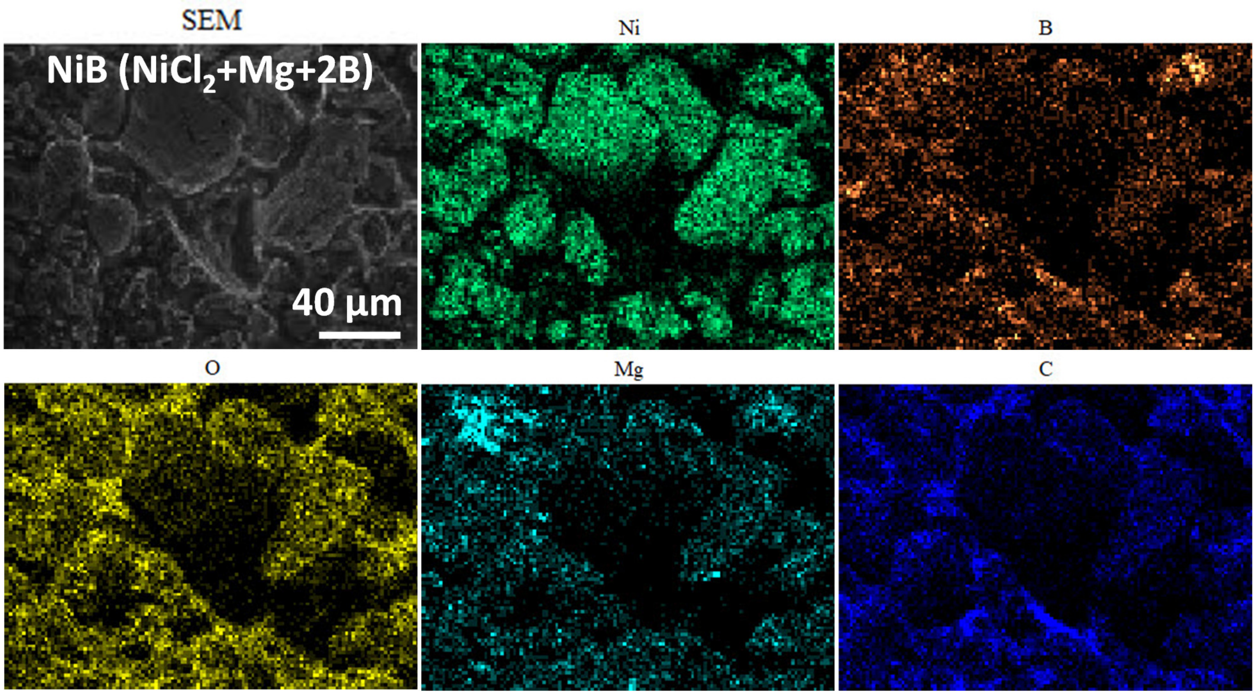

Figure S40. Post-negative potential HER chronoamperometry EDS maps of NiB formed from $NiCl_2$+Mg+2B reaction. Images are from powders embedded on C_{wax} tip.

Table S15. Summary of HER electrocatalysis with SSM synthesized metal borides in 1.0 M KOH.

**Sample**	**(η_{10}) (mV)[1]**	**(η_{20}) (mV)**	**Tafel (mV/dec)**
10%Pt/C	-33 ± 3	-95 ± 4	28
FeB ($FeCl_3$/1.5MgB_2)	-368 ± 25	-442 ± 22	157
FeB ($FeCl_3$/1.5Mg/B)	-361 ± 27<br>(-254 ± 9)	-467 ± 23<br>(-315 ± 9)	155
CoB ($CoCl_2$/MgB_2)	-293 ± 3	-364 ± 3	109
CoB ($CoCl_2$/Mg/2B)	-279 ± 2<br>(-244 ± 4)	-354 ± 3<br>(-288 ± 3)	100
NiB ($NiCl_2$/MgB_2)	-312 ± 6	-371 ± 6	112
NiB ($NiCl_2$/Mg/2B)	-307 ± 8<br>(-276 ± 3)	-367± 10<br>(-323 ± 4)	111

1) Overpotentials reported versus RHE and current densities normalized to geometric electrode area of 0.08 cm^2 (85% iR compensation results in parentheses). Deviations are for 50 LSV runs or 10 LSV runs at 85% iR compensation.

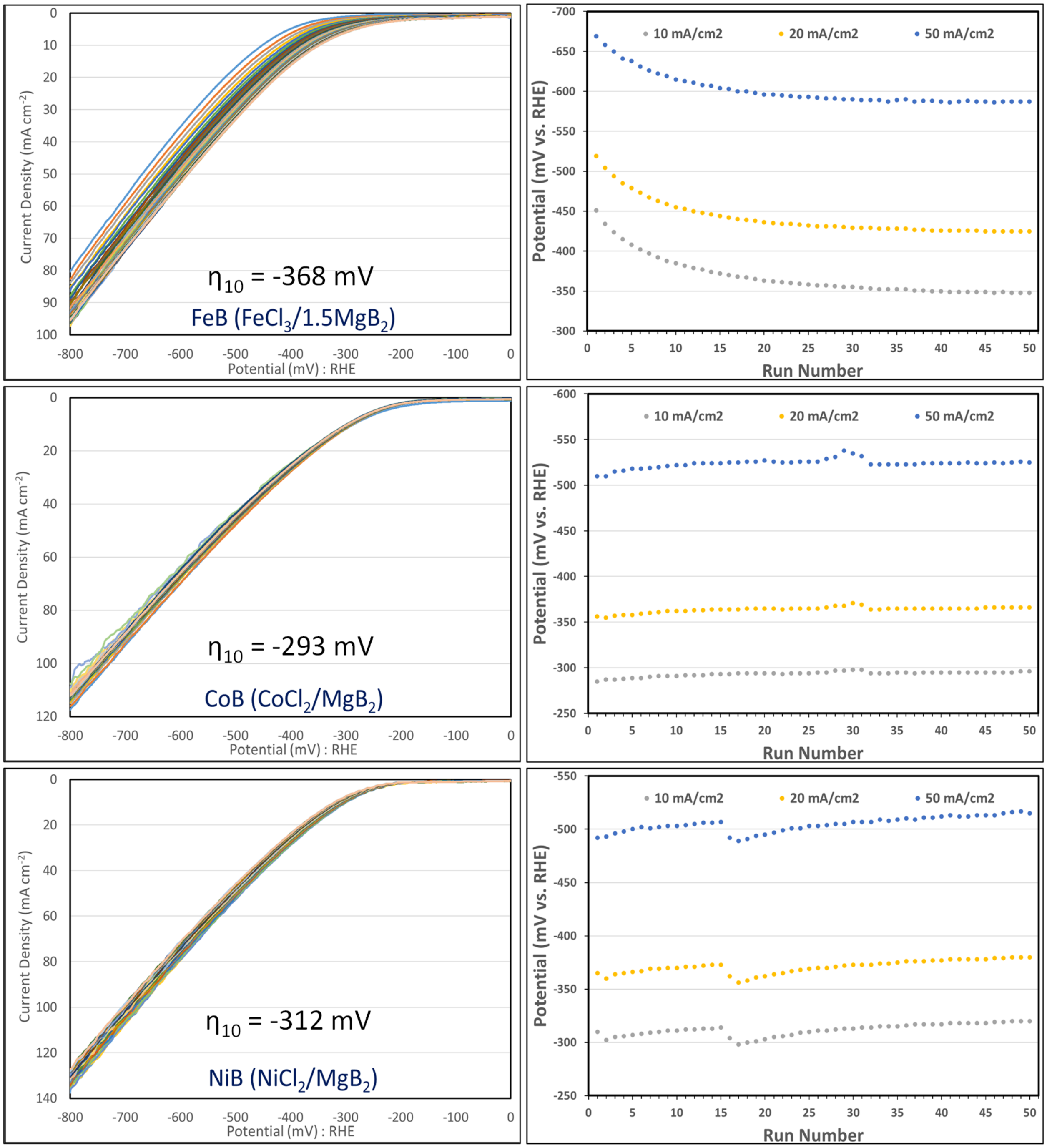

Figure S41A. Left column: Overlay plots of 50 LSV scans of MCl_x/MgB_2 reaction for HER in 1.0 M KOH at 5 mV/s scan rate. Right column: Plots of run number versus potentials at 10, 20, and 50 mA/cm^2 current densities extracted from left column overlay plots. The working electrodes are metal boride powders embedded on C_{wax} tips.

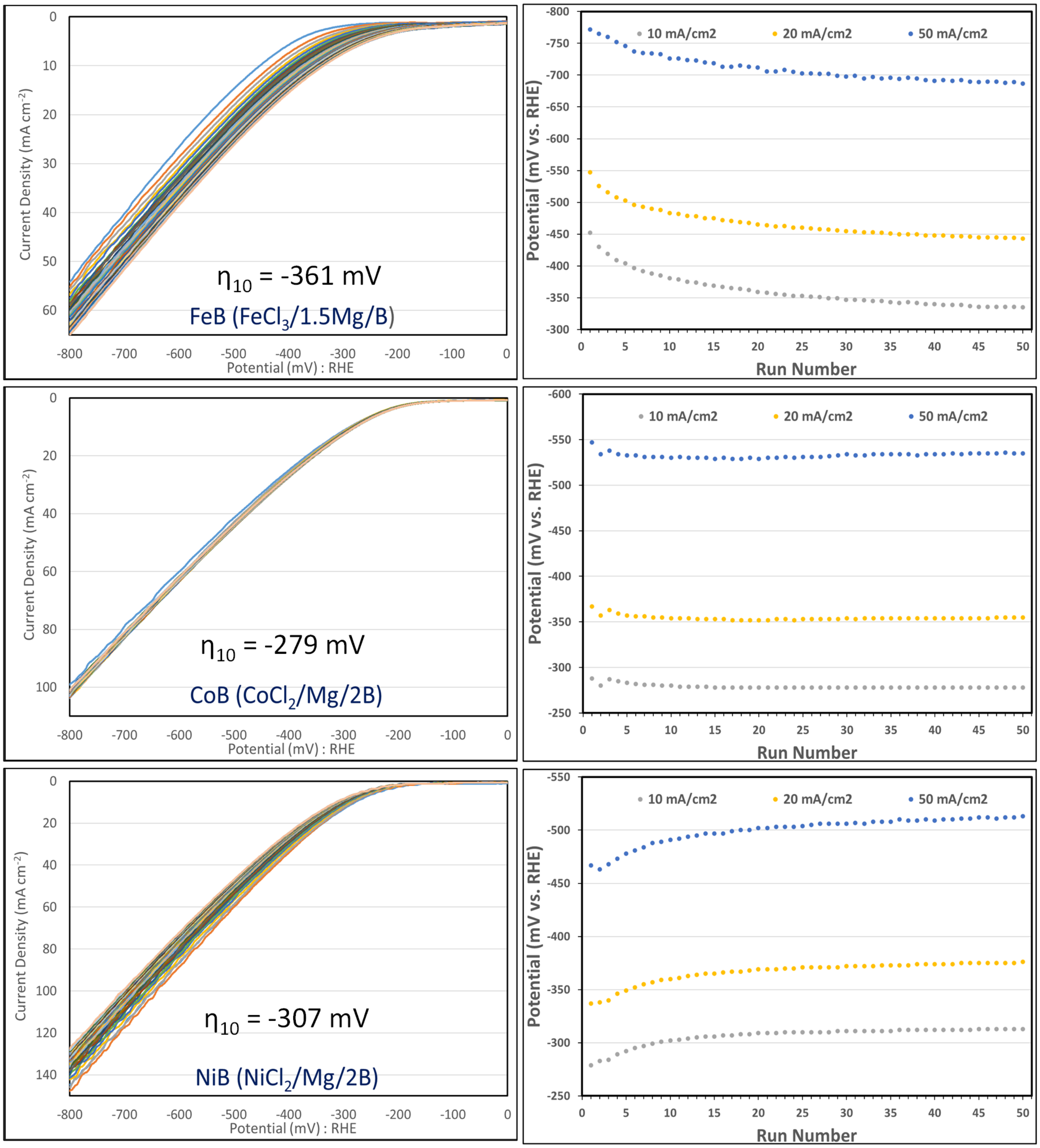

Figure S41B. Left column: Overlay plots of 50 LSV scans of MCl_x/Mg/B reaction for HER in 1.0 M KOH at 5 mV/s scan rate. Right column: Plots of run number versus potentials at 10, 20, and 50 mA/cm^2 current densities extracted from left column overlay plots. The working electrodes are metal boride powders embedded on C_{wax} tips.

Table S16. Summary of HER electrocatalysis with SSM synthesized metal borides in 0.5 M H_2SO_4.

**Sample**	**(η_{10}) (mV)[1]**	**(η_{20}) (mV)**	**Tafel (mV/dec)**	**ECSA (cm^2)**
10%Pt/C	-53 ± 8	-125 ± 4	48	28
FeB ($FeCl_3$/1.5MgB_2)	-357 ± 10	-428 ± 10	115	21
FeB ($FeCl_3$/1.5Mg/B)	-357 ± 5<br>(-323 ± 1)	-449 ± 4<br>(-370 ± 2)	142	--
CoB ($CoCl_2$/MgB_2)	-266 ± 7	-337 ± 7	109	1.0
CoB ($CoCl_2$/Mg/2B)	-288 ± 18<br>(-259 ± 1)	-378 ± 20<br>(-300 ± 1)	114	--
NiB ($NiCl_2$/MgB_2)	-294 ± 16	-375 ± 16	96	0.7
NiB ($NiCl_2$/Mg/2B)	-309 ± 10<br>(-297 ± 2)	-376 ± 11<br>(-346 ± 2)	123	--

1) Overpotentials reported versus RHE and current densities normalized to geometric electrode area of 0.08 cm^2 (85% iR compensation results in parentheses). Deviations are for 50 LSV runs or 10 LSV runs at 85% iR compensation.

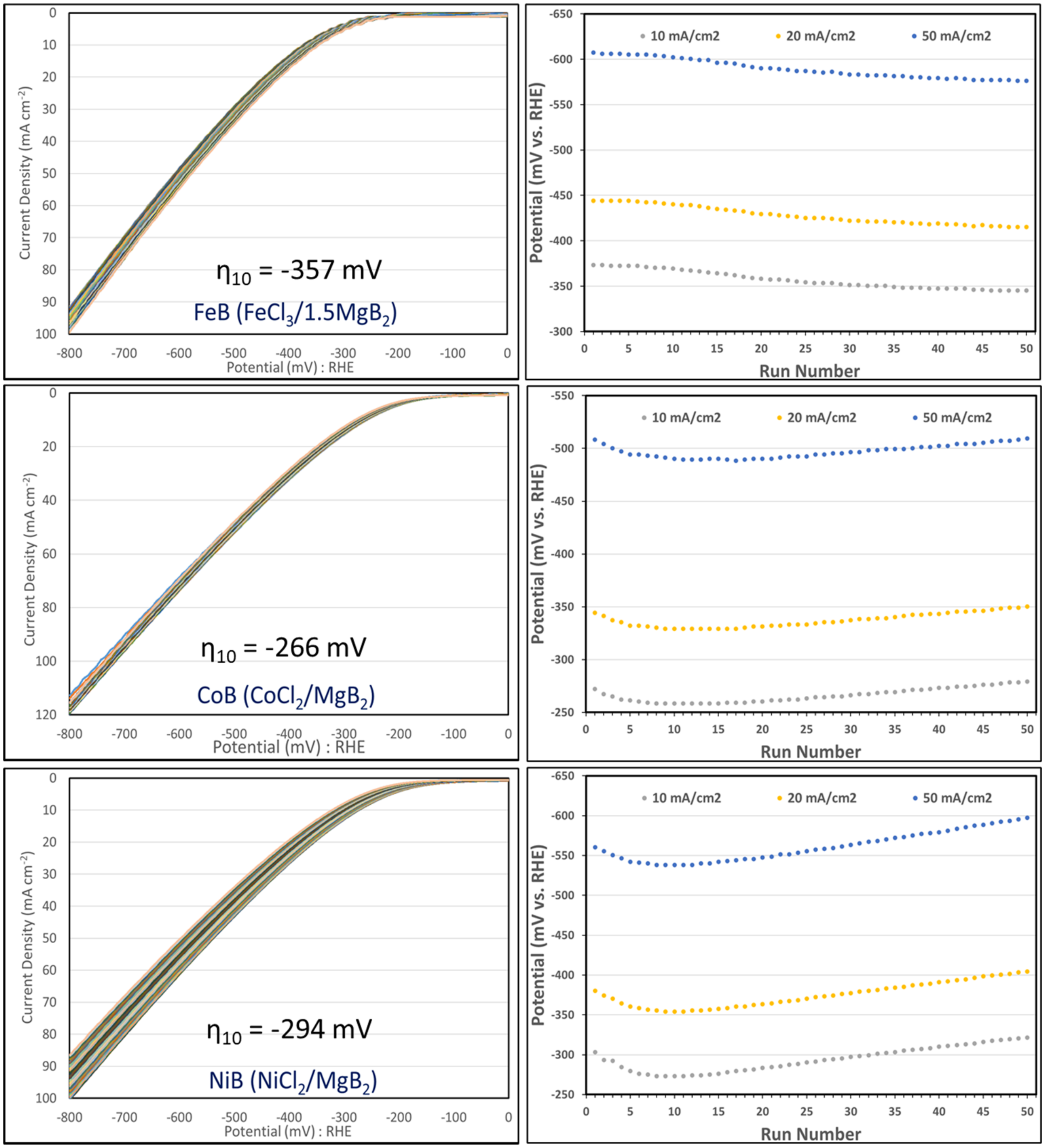

Figure S42A. Left column: Overlay plots of 50 LSV scans of MCl_x/MgB_2 reaction for HER in 0.5 M H_2SO_4 at 5 mV/s scan rate. Right column: Plots of run number versus potentials at 10, 20, and 50 mA/cm^2 current densities extracted from left column overlay plots. The working electrodes are metal boride powders embedded on C_{wax} tips.

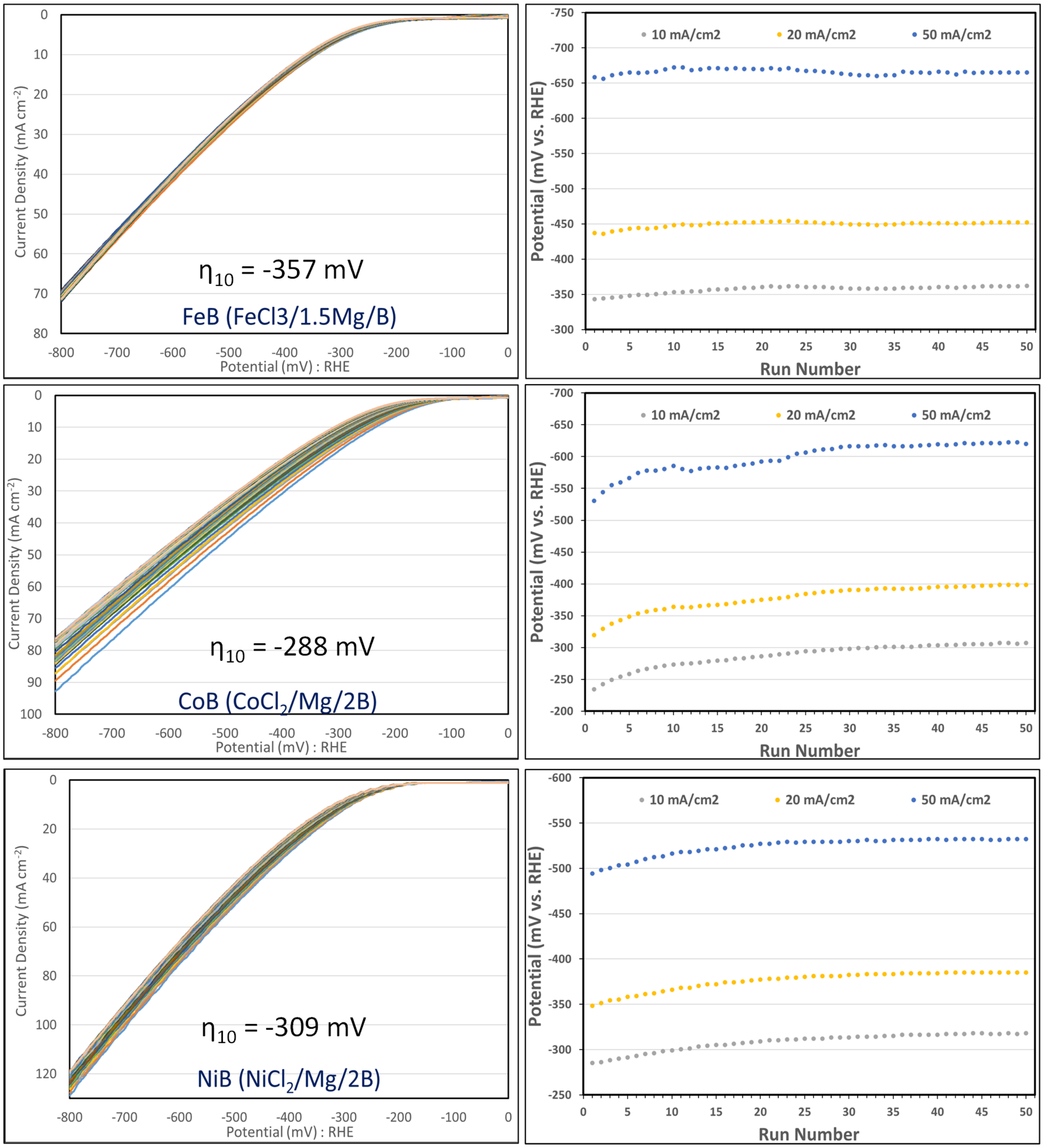

Figure S42B. Left column: Overlay plots of 50 LSV scans of MCl_x/Mg/B reaction for HER in 0.5 M H_2SO_4 at 5 mV/s scan rate. Right column: Plots of run number versus potentials at 10, 20, and 50 mA/cm^2 current densities extracted from left column overlay plots. The working electrodes are metal boride powders embedded on C_{wax} tips.

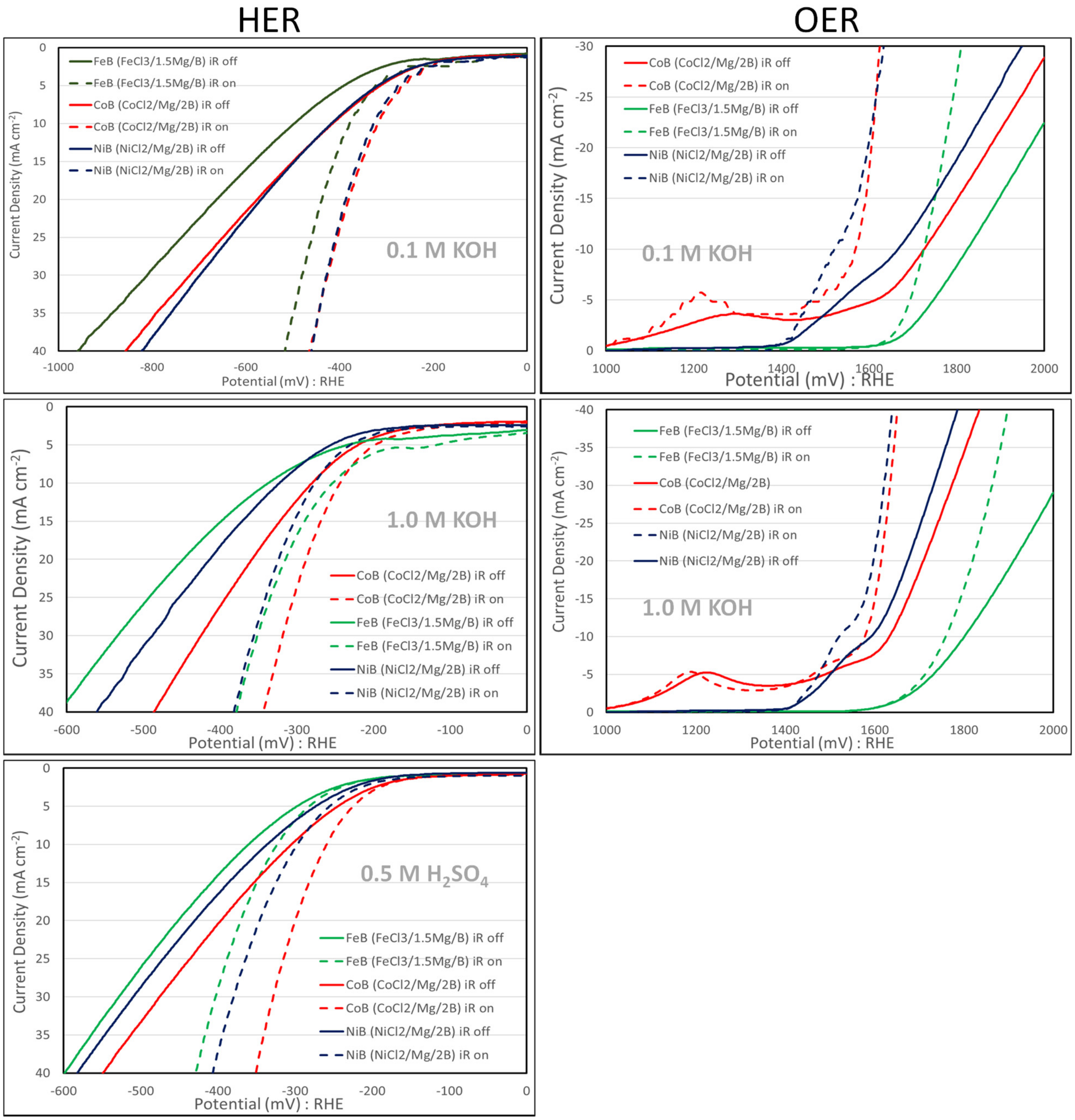

Figure S43. HER and OER LSV overlay plots for metal borides (MCl_x/Mg/B) without iR compensation (solid lines) and with 85% iR compensation (dash lines) at 5 mV/s scan rate. The left-side plots are HER LSVs in (top) 0.1 M KOH, (middle) 1.0 M KOH, and (bottom) 0.5 M H_2SO_4, while the right-side plots are OER LSVs in (top) 0.1 M KOH and (bottom) 1.0 M KOH, respectively. A representative LSV run from 10 LSVs with iR on and off is shown in each graph. The working electrodes are metal boride powders embedded on C_{wax} tips.

References

1. Mavel, G.; Escard, J.; Costa, P.; Castaing, J., ESCA surface study of metal borides. *Surface Science* **1973,** *35*, 109-116.
2. Schreifels, J.; Maybury, P. C.; Swartz Jr., W. E., X-Ray photoelectron spectroscopy of nickel boride catalysts: Correlation of surface states with reaction products in the hydrogenation of acrylonitrile. *J. Catal.* **1980,** *65* (1), 195-206.
3. Naumkin, A. V.; Kraut-Vass, A.; Gaarenstroom, S. W.; Powell, C. J., NIST X-ray Photoelectron Spectroscopy Database https://srdata.nist.gov/xps/Default.aspx (Date accessed: 12/13/2021), 2012; Vol. Volume 20, Version 4.1.
4. Binnewies, M.; Milke, E., *Thermochemical Data of Elements and Compounds*. Weinheim: New York, 1999; p 88-344.
5. Kubaschewski, O.; Alcock, C. B., *Metallurgical Thermochemistry*. 5th ed.; Pergamon Press Inc., Maxwell House: Elmsford, New York, 1979; Vol. International Series on Materials Science and Technology.
6. CRC Handbook of Chemistry and Physics, 102nd Edition (Internet Version 2021). Rumble, J. R., Ed. CRC Press/Taylor & Francis: Boca Raton, FL.
7. NIST Chemistry Webbook. Standard Reference Database. https://webbook.nist.gov/chemistry/ (Date accessed: 7/5/2021), 2018.
8. Li, H.; Wen, P.; Li, Q.; Dun, C.; Xing, J.; Lu, C.; Adhikari, S.; Jiang, L.; Carroll, D. L.; Geyer, S. M., Earth-Abundant Iron Diboride (FeB_2) Nanoparticles as Highly Active Bifunctional Electrocatalysts for Overall Water Splitting. *Adv. Energy Mater.* **2017,** *7* (17), 1700513.
9. Ma, X.; Wen, J.; Zhang, S.; Yuan, H.; Li, K.; Yan, F.; Zhang, X.; Chen, Y., Crystal Co_xB (x = 1-3) Synthesized by a Ball-Milling Method as High-Performance Electrocatalysts for the Oxygen Evolution Reaction. *ACS Sustainable Chem. Eng.* **2017,** *5* (11), 10266-10274.
10. Klemenz, S.; Schuch, J.; Hawel, S.; Zieschang, A.-M.; Kaiser, B.; Jaegermann, W.; Albert, B., Synthesis of a Highly Efficient Oxygen-Evolution Electrocatalyst by Incorporation of Iron into Nanoscale Cobalt Borides. *ChemSusChem* **2018,** *11* (18), 3150-3156.
11. Masa, J.; Weide, P.; Peeters, D.; Sinev, I.; Xia, W.; Sun, Z.; Somsen, C.; Muhler, M.; Schuhmann, W., Amorphous Cobalt Boride (Co_2B) as a Highly Efficient Nonprecious Catalyst for Electrochemical Water Splitting: Oxygen and Hydrogen Evolution. *Adv. Energy Mater.* **2016,** *6*, 1502313.
12. Xu, X.; Deng, Y.; Gu, M.; Sun, B.; Liang, Z.; Xue, Y.; Guo, Y.; Tian, J.; Cui, H., Large-scale synthesis of porous nickel boride for robust hydrogen evolution reaction electrocatalyst. *Appl. Surf. Sci.* **2019,** *470*, 591-595.
13. Masa, J.; Piontek, S.; Wilde, P.; Antoni, H.; Eckhard, T.; Chen, Y.-T.; Muhler, M.; Apfel, U.-P.; Schuhmann, W., Ni-Metalloid (B, Si, P, As, and Te) Alloys as Water Oxidation Electrocatalysts. *Adv. Energy Mater.* **2019,** *9*, 1900796.
14. Jothi, P. R.; Zhang, Y.; Yubuta, K.; Culver, D. B.; Conley, M.; Fokwa, B. P. T., Abundant Vanadium Diboride with Graphene-like Boron layers for Hydrogen Evolution. *ACS Appl. Energy Mater.* **2019,** *2* (1), 176-181.
15. Mazanek, V.; Nahdi, H.; Luxa, J.; Sofer, Z.; Pumera, M., Electrochemistry of layered metal diborides. *Nanoscale* **2018,** *10* (24), 11544-11552.
16. Kirshenbaum, M. J.; Richter, M. H.; Dasog, M., Electrochemical Water Oxidation in Acidic Solution Using Titanium Diboride (TiB_2) Catalyst. *ChemCatChem* **2019,** *11* (16), 3877-3881.
17. Lim, C. S.; Sofer, Z.; Mazanek, V.; Pumera, M., Layered titanium diboride: towards exfoliation and electrochemical applications. *Nanoscale* **2015,** *7* (29), 12527-12534.
18. Zhuang, Z.; Li, Y.; Li, Z.; Lv, F.; Lang, Z.; Zhao, K.; Zhou, L.; Moskaleva, L.; Guo, S.; Mai, L., MoB/g-C_3N_4 Interface Materials as a Schottky Catalyst to Boost Hydrogen Evolution. *Angew. Chem. Int. Ed.* **2018,** *57* (2), 496-500.

19. Jothi, P. R.; Zhang, Y.; Scheifers, J. P.; Park, H.; Fokwa, B. P. T., Molybdenum diboride nanoparticles as a highly efficient electrocatalyst for the hydrogen evolution reaction. *Sustainable Energy Fuels* **2017,** *1*, 1928-1934.
20. Wang, Y.; Mayorga-Martinez, C. C.; Chia, X. Y.; Sofer, Z.; Latiff, N. M.; Pumera, M., Bipolar Electrochemistry as a Simple Synthetic Route toward Nanoscale Transition of Mo_2B_5 and W_2B_5 for Enhanced Hydrogen Evolution Reaction. *ACS Sustainable Chem. Eng.* **2019,** *7* (14), 12148-12159.
21. Li, Q.; Zou, X.; Ai, X.; Chen, H.; Sun, L.; Zou, X., Revealing Activity Trends of Metal Diborides Toward pH-Universal Hydrogen Evolution Electrocatalysts with Pt-Like Activity. *Adv. Energy Mater.* **2019,** *9*, 1803369.
22. Guo, F.; Wu, Y.; Ai, X.; Chen, H.; Li, G.-D.; Chen, W.; Zou, X., A class of metal diboride electrocatalysts synthesized by a molten salt-assisted reaction for the hydrogen evolution reaction. *Chem. Commun.* **2019,** *55* (59), 8627-8630.
23. Nsanzimana, J. M. V.; Peng, Y.; Xu, Y. Y.; Thia, L.; Wang, C.; Xia, B. Y.; Wang, X., An Efficient and Earth-Abundant Oxygen-Evolving Electrocatalyst Based on Amorphous Metal Borides. *Adv. Energy Mater.* **2018,** *8*, 1701475.
24. Zieschang, A.-M.; Bocarsly, J. D.; Schuch, J.; Reichel, C. V.; Kaiser, B.; Jaegermann, W.; Seshadri, R.; Albert, B., Magnetic and Electrocatalytic Properties of Nanoscale Cobalt Boride, Co_3B. *Inorg. Chem.* **2019,** *58*, 16609-16617.
25. Gupta, S.; Patel, N.; Miotello, A.; Kothari, D. C., Cobalt-Boride: An efficient and robust electrocatalyst for Hydrogen Evolution Reaction. *J. Power Sources* **2015,** *279*, 620-625.
26. Gupta, S.; Patel, N.; Fernandes, R.; Kadrekar, R.; Dashora, A.; Yadav, A. K.; Bhattacharyya, D.; Jha, S. N.; Miotello, A.; Kothari, D. C., Co–Ni–B nanocatalyst for efficient hydrogen evolution reaction in wide pH range. *Appl. Catal. B* **2016,** *192*, 126-133.
27. Li, Y.; Xu, H.; Huang, H.; Gao, L.; Zhao, Y.; Ma, T., Synthesis of Co–B in porous carbon using a metal-organic framework (MOF) precursor: A highly efficient catalyst for the oxygen evolution reaction. *Electrochem. Commun.* **2018,** *86*, 140-144.
28. Jiang, Y.; Fang, Y.; Chen, C.; Ni, P.; Kong, B.; Song, Z.; Lu, Y.; Niu, L., Amorphous Cobalt Boride Nanosheets Directly Grown on Nickel Foam: Controllable Alternately Dipping Deposition for Efficient Oxygen Evolution. *ChemElectroChem* **2019,** *6* (14), 3684-3689.
29. Hao, W.; Wu, R.; Zhang, R.; Ha, Y.; Chen, Z.; Wang, L.; Yang, Y.; Ma, X.; Sun, D.; Fang, F.; Guo, Y., Electroless Plating of Highly Efficient Bifunctional Boride-Based Electrodes toward Practical Overall Water Splitting. *Adv. Energy Mater.* **2018,** *8* (26), 1801372.
30. Tan, T.; Han, P.; Cong, H.; Cheng, G.; Luo, W., An Amorphous Cobalt Borate Nanosheet-Coated Cobalt Boride Hybrid for Highly Efficient Alkaline Water Oxidation Reaction. *ACS Sustainable Chem. Eng.* **2019,** *7*, 5620-5625.
31. Arivu, M.; Masud, J.; Umapathi, S.; Nath, M., Facile synthesis of Ni_3B/rGO nanocomposite as an efficient electrocatalyst for the oxygen evolution reaction in alkaline media. *Electrochem. Commun.* **2018,** *86*, 121-125.
32. Masa, J.; Andronescu, C.; Antoni, H.; Sinev, I.; Seisel, S.; Elumeeva, K.; Barwe, S.; Marti-Sanchez, S.; Arbiol, J.; Cuenya, B. R.; Muhler, M.; Schuhmann, W., Role of Boron and Phosphorus in Enhanced Electrocatalytic Oxygen Evolution by Nickel Borides and Nickel Phosphides. *ChemElectroChem* **2019,** *6*, 235-240.
33. Zeng, M.; Wang, H.; Zhao, C.; Wei, J.; Qi, K.; Wang, W.; Bai, X., Nanostructured Amorphous Nickel Boride for High-Efficiency Electrocatalytic Hydrogen Evolution over a Broad pH Range. *ChemCatChem* **2016,** *8* (4), 708-712.
34. Cao, M.; Zhang, X.; Qin, J.; Liu, R., Enhancement of Hydrogen Evolution Reaction Performance of Graphitic Carbon Nitride with Incorporated Nickel Boride. *ACS Sustainable Chem. Eng.* **2018,** *6* (12), 16198-16204.
